# Connective tissue growth factor contributes to resistance to anti-angiogenic therapies in renal cancer

**DOI:** 10.7150/thno.125269

**Published:** 2026-02-11

**Authors:** Manon Teisseire, Arthur Karaulic, Julien Parola, Maëva Totobesola, Delphine Borchiellini, Tanguy Pace-Loscos, Renaud Schiappa, Emmanuel Chamorey, Jérôme Durivault, Maëva Dufies, Damien Ambrosetti, Frédéric Luciano, Juan Gao, Yihai Cao, Gilles Pagès, Sandy Giuliano

**Affiliations:** 1 Université Côte d'Azur, Institute for research on cancer and aging of Nice, CNRS UMR 7284; INSERM U1081, Centre Antoine Lacassagne, France.; 2 Department of medical oncology, Centre Antoine Lacassagne, Nice, France.; 3 Department of statistics, Centre Antoine Lacassagne, Nice, France.; 4 Centre Scientifique de Monaco, Biomedical Department, 8 quai Antoine Premier, 98000, Monaco, Monaco.; 5 Department of Pathology, CHU Nice, Université Côte d'Azur, Nice, France.; 6 Department of Microbiology, Tumor and Cell Biology, Karolinska Institutet, 171 77 Stockholm, Sweden.; 7 LIA ROPSE, Laboratoire International Associé Université Côte d'Azur—Centre Scientifique de Monaco.

## Abstract

**Background:**

Clear cell renal cell carcinoma (ccRCC) is predominantly treated with anti-angiogenic therapies (AATs), such as sunitinib and axitinib. While these therapies initially improve outcomes, resistance frequently emerges, limiting long-term efficacy. Understanding the molecular mechanisms underlying AAT resistance is essential to optimize treatment strategies.

**Methods:**

To identify factors involved in AAT resistance, we performed integrated transcriptomic and proteomic analyses on ccRCC cell lines subjected to either transient AAT treatment or with established acquired resistance. Functional validation was performed using *in vitro* assays (proliferation, migration, invasion) and *in vivo* zebrafish models. Plasma levels of candidate proteins were also measured in ccRCC patients and correlated with clinical outcomes.

**Results:**

Connective Tissue Growth Factor (CTGF) was consistently upregulated following treatment and in resistant cell lines. CTGF, a secreted protein regulated by Yes-associated protein (YAP) in the Hippo pathway, is known to promote angiogenesis, fibrosis, and tumor progression. Functionally, CTGF enhanced tumor cell aggressiveness *in vitro* and *in vivo*. Patient-derived samples also exhibited elevated CTGF levels in resistant tumors. Crucially, higher plasma CTGF levels were associated with shorter progression-free survival in ccRCC patients receiving AATs.

**Conclusion:**

CTGF is a key mediator of resistance to AATs in ccRCC, by promoting tumor progression and remodeling the tumor microenvironment. CTGF may thus serve as both a predictive biomarker and a therapeutic target. These findings support further investigation of CTGF inhibition as a strategy to overcome AAT resistance and improve treatment outcomes in ccRCC patients.

## Introduction

Clear cell renal cell carcinoma (ccRCC) presents a significant therapeutic challenge in oncology, and its incidence is steadily increasing. Although anti-angiogenic therapies (AATs) have significantly improved the management of metastatic ccRCC, the development of resistance limits their long-term effectiveness. Understanding the molecular mechanisms underlying this resistance is essential for optimizing current treatments and developing more durable and effective therapeutic strategies.

AATs, such as VEGFR tyrosine kinase inhibitors (TKIs), including sorafenib (Nexavar) 1, sunitinib (Sutent) 2, axitinib (Inlyta) 3 and pazopanib (Votrient) 4, effectively inhibit tumor angiogenesis and have become a cornerstone of ccRCC treatment. These agents have demonstrated clinical benefit, in particular by improving progression-free survival. Despite their initial efficacy, however, clinical observations show that AATs are invariably followed by relapse, with resistance emerging at different times among patients. This pattern of transient response highlights the urgent need to better understand the adaptive mechanisms by which tumors evade AATs.

A key area of investigation in ccRCC is the role of the tumor microenvironment (TME) in mediating resistance, through its dynamic remodeling. The TME, which includes the extracellular matrix (ECM), stromal cells, and immune components, plays a critical role in tumor progression and therapeutic response. Of particular interest are secreted factors that drive TME remodeling, especially those promoting fibrosis. These factors contribute to the formation of a protective niche that shelters tumor cells, impedes drug penetration, and activates compensatory pro-tumorigenic and pro-angiogenic pathways 5.

Intratumor fibrosis (ITF) is commonly observed in ccRCC, with studies reporting its presence in up to 81.7% of cases, correlating with higher Fuhrman grade and lymphatic invasion 6.

Resistance to AATs arises through multiple mechanisms, including activation of alternative pro-angiogenic pathways (FGF2, SDF-1) and recruitment of bone marrow-derived cells 7-10.

Beyond these mechanisms, accumulating evidence suggests that anti-angiogenic therapies can actively promote fibrotic remodeling of the tumor microenvironment, potentially reinforcing adaptive resistance pathways.

One of the most important factors mediating fibrosis is transforming growth factor beta (TGFβ) 11 and a key downstream effector of TGFβ-induced fibrosis is connective tissue growth factor, also known as cellular communication network factor 2 (CTGF/CCN2; hereafter referred to as CTGF) 12,13. CTGF is a matricellular protein involved in extracellular matrix remodeling and stromal activation, and its expression has been associated with fibrotic tumor microenvironments and aggressive tumor features in ccRCC. However, its specific contribution to resistance to anti-angiogenic therapies has not been directly established.

CTGF is a critical transcriptional target of Yes-associated protein (YAP), a major effector of the Hippo signaling pathway. The YAP-CTGF axis is crucial for regulating both physiological and pathological processes 14-16. YAP enhances CTGF transcription in response to extracellular stimuli such as growth factors and mechanical stress, with YAP's nuclear localization being essential for activating target genes like CTGF 17,18. Whether activation of this pathway contributes to adaptive responses to anti-angiogenic therapies in ccRCC remains unknown.

To further elucidate the signaling pathways involved in resistance to AATs, we conducted integrated transcriptomic and proteomic analyses on ccRCC cells treated with sunitinib, as well as on cells that had acquired resistance to the drug 19. Proteomic profiling was performed using conditioned media, based on the hypothesis that resistance may be driven by secreted factors that remodel the tumor microenvironment following prolonged drug exposure 20. The results identified CTGF as one of the most significantly upregulated secreted proteins in the resistant state.

The analyses confirmed CTGF as a key player in AAT resistance. The objective of our study was to investigate the role of CTGF in various hallmarks of tumor aggressiveness and to assess its potential as a therapeutic target. Additionally, we aimed to evaluate CTGF as a prognostic marker and/or a predictive factor for sensitivity to AATs.

## Materials and Methods

### Reagents and antibodies

Recombinant CTGF was obtained from Peprotech, diluted in PBS-0.1%BSA and stored at -80 °C. Antibodies used including anti-CTGF (#86641), anti-YAP (#14074), anti-PYAP (Ser127) (#4911), anti-HSP60 (#12165) and anti-HSP90 (#4874) were purchased from Cell Signaling Technology. Sunitinib came from unconsumed medications given to patients (Centre Antoine Lacassagne, Nice, France), prepared as a 5 mM stock solution in DMSO and stored at -20 °C. Axitinib (TargetMol, AG-013736) was prepared in DMSO at 5 mM and stored at -20 °C. Verteporfin (MedChem Express, HY-B0146) was dissolved in DMSO at 1 mM and stored at -80 °C. QV-D-OPh (MedChem Express, HY-12305) was dissolved in DMSO at 10 mM and stored at -20 °C. For labeling cells in zebrafish experiments, Vybrant™ CM-DiI Cell-Labeling Solution (Thermo Fisher Scientific, V22888) was used.

### Cell culture

The 786-O (CRL-1932) and A498 (HTB-44) cell lines were obtained from the American Type Culture Collection (ATCC), while RCC10 cells were generously provided by W.H. Kaelin (Dana-Farber Cancer Institute, Boston, MA) 21. RCC cells were cultured in high-glucose DMEM supplemented with GlutaMAX™, pyruvate, and 7% FBS. Sunitinib-resistant cells have been previously described [20], and axitinib-resistant cells were generated through chronic exposure to increasing concentrations of axitinib, up to 8 μM (**Figure S1**). Primary human RCC cells were isolated by enzymatic dissociation from surgical specimens provided by Dr. D. Ambrosetti (CHU Nice, Department of Pathology) and cultured in PromoCell Renal Epithelial Cell Growth Medium 2.

#### Cell viability

15, 000 786-O, sunitinib (SUNR) or axitinib (AXIR) resistant cells were first treated with 10 μM of Q-VD-OPh, an irreversible pan-caspase inhibitor and then transfected for 96 h with siRNA control (siCT) or CTGF (siCTGF) and treated with an AAT (sunitinib or axitinib). Then, both the supernatant and the cells were collected and incubated with propidium iodide (PI) at a concentration of 2.5 µg/mL (BioLegend) for 5 min. The proportion of PI-positive cells was then determined by flow cytometry using a CytoFLEX instrument (Beckman Coulter).

### Immunoblotting

786-O cells were either transfected with siRNA (with or without sunitinib or axitinib) or treated with Verteporfin (VP) alone or in combination with sunitinib or axitinib and lysed in ice-cold lysis buffer (40 mM HEPES (pH 7.4), 120 mM NaCl, 1 mM EDTA, 1% Triton X-100, 10 mM glycerol 2-phosphate, 10 mM sodium pyrophosphate, 0.5 mM sodium orthovanadate, and 50 mM NaF). Prior to cell lysis, 1 μM Microcystin-LR and a protease inhibitor cocktail were added to the buffer. Protein samples (30 μg) were separated by 10% SDS-PAGE, transferred onto nitrocellulose membranes, and probed with the appropriate primary antibodies. Detection of proteins was performed using the ECL system with horseradish peroxidase-conjugated anti-rabbit or anti-mouse antibodies.

### Quantitative real-time PCR (qPCR)

One microgram of total RNA was used for reverse transcription using the QuantiTect Reverse Transcription kit (QIAGEN, Hilden, Germany) with a mix of oligo (dT) and random primers to prime first-strand synthesis. SYBR master mix plus (Eurogentec, Liege, Belgium) was used for qPCR. The CTGF oligo are as follows: Forward: 5'-GCCTCCTGCAGGCTAGAGAA-3'; Reverse: 5'-GGCCGTCGGTACATACTCCA-3' (size of the amplicon 195 bp). The mRNA level was normalized to *36B4* mRNA.

### Immunofluorescence

786-O RCC cells were seeded on glass coverslips (60,000 cells per well) in 6-well plates and treated with the relevant treatment for 48 h. After treatment, cells were washed and fixed with 3% paraformaldehyde at room temperature for 20 min. Cells were then permeabilized with phosphate-buffered saline (PBS) containing 0.2% Triton X-100 (Amresco, 0694-1L) for 2 min. Next, the cells were incubated with anti-YAP primary antibody for 1 hour at room temperature. Following three washes with PBS, the cells were incubated with Alexa Fluor 594-labeled anti-rabbit secondary antibody (1:1000 dilution, Invitrogen, Life Technologies, A21203) for 1 h at room temperature. Finally, the coverslips were mounted using Fluoroshield^TM^ with DAPI (Sigma Aldrich F6057). Fluorescence images were acquired using an EVOS M5000 imaging system (Invitrogen, Thermo Scientific). Quantifications were obtained using ImageJ and normalized by cell area.

### Quantification of CTGF in conditioned media by ELISA

Conditioned media of cells treated or not with sunitinib were recovered for the measurement of CTGF using the Human DuoSet ELISA kit (Catalog #: DY9190 R&D Systems, MN, USA). Results were normalized to the cell count.

### Transient transfection of small interfering RNA

ccRCC cells were seeded in a 6-well plate in 1.5 mL of DMEM medium with 7% FBS. The following day, at 30% confluence, cells were transfected with 25 nM of either siCTGF (SMARTpool, Dharmacon), siYAP (SMARTpool, Dharmacon) or siControl (SMARTpool Dharmacon), using 5 µL Lipofectamine RNAiMAX (Invitrogen) in 600 µL of Opti-MEM for 48 h or 96 h.

### Colony formation assay

786-O RCC cells were seeded at 1000 cells per condition in dishes of diameter 60 mm and cultured for 10 days to allow colony formation. Cells were washed with phosphate-buffered saline (PBS) and fixed and stained at room temperature for 20 min with crystal violet (Sigma, C3886). Then the colony area was measured by ImageJ.

### Enzymatic dissociation of primary RCC cells

Fresh human ccRCC specimens were obtained from the University Hospital of Nice. To establish single-cell cultures, tissue samples were mechanically dissected into 1 mm³ fragments and enzymatically digested with a cocktail of DNase I (Sigma-Aldrich - #11284932001, 0.1 mg/mL), collagenase (Sigma-Aldrich - # C2674), and dispase type II (Gibco - #17105041, 0.4 mg/mL) at 37 °C for 30 min. The resulting suspension was filtered through 100 µm and then 40 µm cell strainers to remove debris. Isolated cells were maintained in PromoCell Renal Epithelial Cell Growth Medium 2 (PromoCell, Heidelberg, Germany), supplemented with its specific SupplementMix. To ensure standardized experimental conditions and allow for complete cellular attachment and recovery from dissociation stress, all experiments were initiated after two trypsinization and seeding rounds. For studies, cells were monitored at 48 h for gene expression and the invasion assay was performed at 4 days.

### Migration assay

#### Scratch assay

786-O CTGF-KO cells were seeded in 12-well plates at a density of 180,000 cells per well. After 24 h, a linear scratch was introduced into the cell monolayer using a 20 µL plastic pipette tip to simulate a wound. 16 h after scratch induction, images of the wound areas were captured, and wound closure was quantified using ImageJ software.

#### Transwell assay

For the transwell migration assay, 50,000 ccRCC cells (786-O) were plated in serum-free medium in the upper compartment of pre-wetted inserts (24-well plate-compatible, translucent PET membrane, 8.0 μm pore size, Falcon, Thermo Fisher). The lower chamber was filled with medium containing 7% FBS or CTGF recombinant protein in 0% FBS medium. After 16 h of incubation at 37 °C in a humidified atmosphere with 5% CO2, the migrated cells were fixed with 3% paraformaldehyde for 20 min and stained with crystal violet at room temperature. The number of migrated cells was counted using ImageJ.

### Spheroid formation and invasion assay

First, the 786-O RCC cells or primary RCC cells were transfected with siRNA (25 nM siCt or siCTGF) in 6-well plates. For spheroid generation, 500 µL of 10,000 cells/well were seeded into 1.5% agarose-coated 24-well plates. After 48 h of spheroid initiation, spheroids were embedded in 1 mg/mL Matrigel (Corning Matrigel Matrix, 356237), with 500 µL DMEM + 7% FBS added on top. Sunitinib (1 µM) and axitinib (1 µM) treatments began at Matrigel inclusion, added to both the Matrigel and medium. Spheroid invasion was assessed by measuring the invasion area at 0, 3, and 5 days with ImageJ. Results are presented at day 5.

### Zebrafish tumor model

Animal experiments received approval by the Northern Stockholm Experimental Animal Ethical Committee. Zebrafish embryos of the transgenic strain expressing enhanced GFP under the fli1 promoter (Fli1: EGFP) were cultivated at a temperature of 28 °C under standard experimental conditions. At 24 h post fertilization (hpf), zebrafish embryos were exposed to an aquarium solution containing 0.2 mM of 1-phenyl-2-thiourea (PTU, Sigma). Upon reaching 48 hpf, Fli1: EGFP zebrafish embryos were dechorionated using sharp-tip forceps and were anesthetized with 0.04 mg/mL of tricaine (MS-222, Sigma) prior to microinjection. *In vitro*, 786-O naïve or sunitinib-resistant (SUNR) cells were labelled with 2 μg/mL Vybrant CM-Dill cell-labeling solution. The labeled cells were suspended in DMEM containing 2 mM EDTA. Subsequently, 5 nL of the cell solution was injected into the perivitelline space (PVS) of each embryo using an Eppendorf microinjector (Femto-Jet 5247, Eppendorf) and a MM33-Right Manipulator (Märzhäuser Wetzlar). Non-filamentous borosilicate glass capillary needles were employed for the injection procedure. The injected zebrafish embryos were promptly transferred to PTU-enriched aquarium water. Over the course of 48 h, fluorescent microscopy (Nikon Eclipse C1) was used to monitor the embryos, investigating tumor growth and tumor invasion and metastasis.

### CRISPR/Cas9 generation

The selected gRNA target regions were designed using the ChopChop web tool 22. The gRNA sequences used in this study are as follows:

CrCCN2_ex3 5' **G**AAGACTCGACTCACCCGCG 3' (#1)

CrCCN2_ex3 5' **G**GTGGTACGGTGTACCGCAG 3' (#5)

CrCCN2_ex3 5' **G**CGAACGTCCATGCTGCACAG 3' (#17)

A bold 'G' was added at the 5' end of each sequence to meet the transcription initiation requirement of the human U6 promoter. 786-O WT cells were transfected with pSpCas9(BB)-2A-GFP (PX458) plasmids using PEI (Polyplus Transfection). The PX458 plasmid, containing CRISPR-Cas9 and the guide RNA sequences, was a gift from Feng Zhang (Addgene plasmid #48138, Watertown, MA, USA).

The pSpCas9(BB)-2A-GFP (pX458) vector expresses GFP, enabling single-cell sorting 24 h post-transfection on a BD FACS Melody (BD Biosciences). Individual GFP-positive cells were expanded in DMEM with 7% FBS, and CTGF expression in each clone was assessed by immunoblotting.

### Measurement of plasma CTGF in patients

Blood samples were obtained during the inclusion visit (baseline) of patients recruited in the TORAVA (NCT00619268) 23 and the SUVEGIL (NCT00943839) 24 clinical trials as already described , and the plasma concentration of CTGF was assessed in correlation with both overall survival (OS) and progression-free survival (PFS). OS was defined as the interval from trial inclusion to death from any cause, while PFS referred to the duration from trial inclusion to disease progression, treatment discontinuation, or death. Data were censored at the time of the last follow-up for patients who were still alive or had not yet experienced disease progression. Blood samples were collected and immediately processed by centrifugation at 10,000 × *g* for 10 min. The resulting plasma was carefully harvested and stored at -80 °C until further analysis. CTGF plasma concentrations were measured using enzyme-linked immunosorbent assay (ELISA) kits (R&D Systems, MN, USA), following the manufacturer's instructions. Prior to analysis, plasma samples were diluted 1:100 to ensure accurate quantification within the assay's dynamic range.

### Proteomic and mass spectrometry analysis

786-O parental cells and their counterparts, 786-O SUNR and 786-O AXIR cells, were used for the analysis. Each condition was prepared in three biological replicates. On day 0, 200,000 786-O parental cells and 350,000 786-O SUNR or AXIR cells were seeded in 10 cm culture dishes, to reach approximately 60-70% confluence. On day 1, cells were treated with sunitinib (5 µM) for 786-O SUNR and axitinib (10 µM) for 786-O AXIR. On day 2, all cells were washed multiple times with serum-free DMEM medium. Cells were then incubated in serum-free DMEM medium for 24 h. On day 3, culture supernatants were collected and centrifuged at 1200 × g for 20 min at 4 °C. The resulting cleared supernatants were further dialyzed and concentrated against 50 mM Ammonium bicarbonate using Amicon4 Ultra, 3 Kd MWCO, (Millipore). Protein extracts were dried using a vacuum concentrator and solubilized in LDS-PAGE loading buffer. 15 µg of each protein extract was loaded on NuPAGE™ 4-12% bis-tris acrylamide gel and stacked as a single band. Bands containing the whole secretome were digested with trypsin sequencing grade (Promega) before mass spectrometry analysis using an Orbitrap Fusion Lumos Tribrid (ThermoFisher Scientific, San Jose, CA) in data-independent acquisition mode (DIA). Protein identification and quantification were performed using the DIA-NN 1.8 algorithm 25 and DIAgui package (https://github.com/marseille-proteomique/DIAgui 26. The statistical analysis was done with the Perseus program (version 1.6.15.0) 27. Differential proteins were detected using a two-sample t-test at 0.05 permutation-based false discovery rate. Proteins with p value < 0.05 were considered significant.

### Online available patient data

Normalized RNA sequencing (RNA-Seq) data of The Cancer Genome Atlas (TCGA) were downloaded from cBiopotal (www.cbioportal.org, TCGA Provisional; RNA-Seq V2) or GEPIA (http://gepia.cancer-pku.cn). Data were available for 1105 tumor samples. The results published here are in whole or in part based upon data generated by the TCGA Research Network http://cancergenome.nih.gov 28,29.

### Statistical analysis

Categorical data are presented as relative and absolute frequencies. Continuous data are presented as mean, median, minimum, maximum and standard deviation. The t-test and Wilcoxon rank sum test were used to assess the similarity in the distributions of the continuous variables. The Pearson and Spearman coefficients were used to measure the dependence between continuous variables. Missing data are reported as an absolute number and as a percentage. Censored data are presented with median follow-up (calculated using the inverse Kaplan-Meier method 30) and the Kaplan-Meier curve. Percent survival and 95% confidence intervals are reported from 0 to 24 months with 6-month intervals. Survival curves were compared using the log-rank test and the hazard ratio was calculated using Cox regression and reported with its 95% confidence interval. Overall survival (OS) was calculated between the start of treatment and the time of death. Progression-free survival (PFS) was calculated between the start of treatment and the date of progression or death. Patients who did not experience an event (death or progression) or who were lost to follow-up were censored at the date of their last contact. For the bivariate survival analysis, the cut-off for continuous variables was determined using the bestcut2 function package in R. All analyses were performed using R version 4.3.1. To assess the characteristics of tumor aggressiveness (proliferation, migration, invasion) data were analyzed using unpaired Student's t-tests or Mann-Whitney tests for pairwise comparisons, and one-way ANOVA or Kruskal-Wallis tests for multiple comparisons, as appropriate. All analyses were performed in GraphPad Prism 10.1.1. A two-sided p-value of 0.05 or less was considered significant. Data are presented as the mean ± standard error of the mean (SEM). All experiments were performed in at least three biological replicates (n = 3) for each group, with each replicate run in triplicate.

## Results

### CTGF is among the most upregulated secreted proteins in AAT-resistant ccRCC cells

To identify secreted factors that contribute to tumor microenvironment remodeling during chronic exposure to AATs, we performed proteomic analyses of conditioned media from both naïve and resistant 786-O ccRCC cells. Conditioned media were collected from 786-O cells treated with sunitinib or axitinib for 48 h, as well as from the same cell lines that had acquired resistance to these agents (designated SUNR and AXIR, respectively).

**Figure 1:**
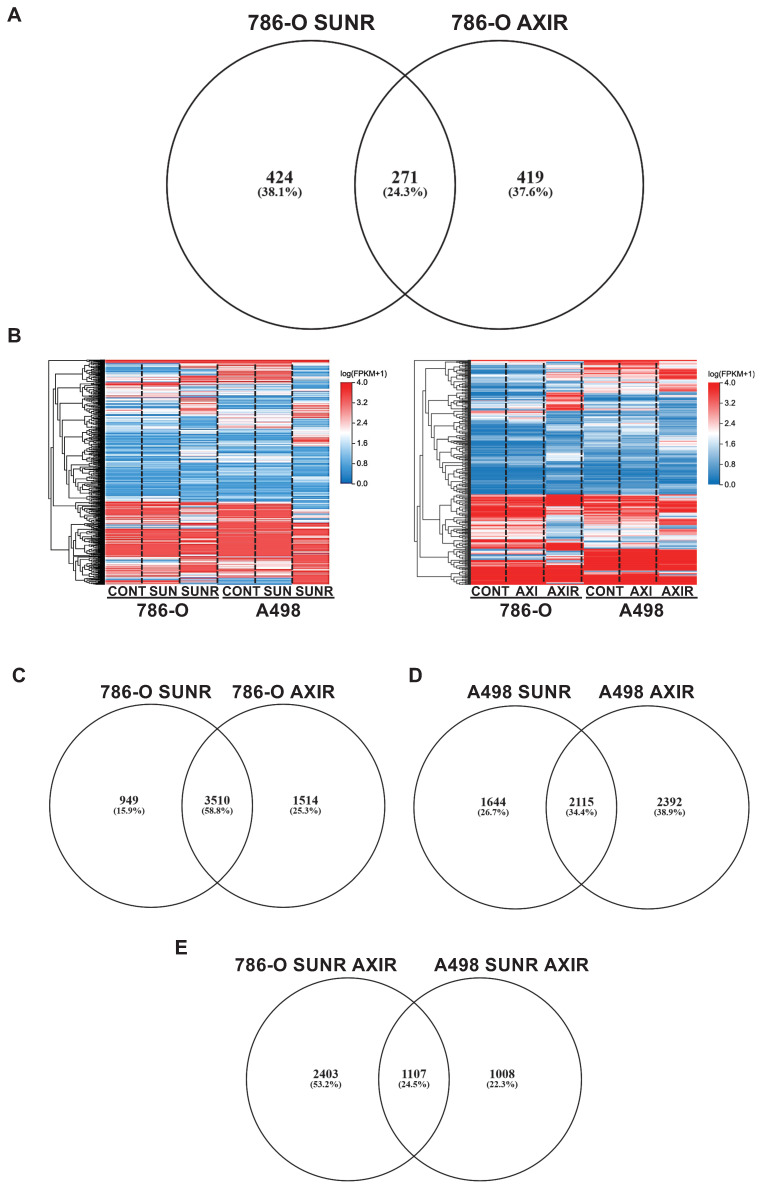
** Chronic exposure to sunitinib or axitinib shapes the transcriptomic and proteomic profiles of ccRCC cells. (A)** Venn diagram of specific and common secreted factors by SUNR and AXIR cells. **(B)** Heatmaps of the most differentially expressed genes between control cells, cells exposed for 48 h to either 2.5 μmol/L of sunitinib (SUN) or axitinib (AXI) and SUNR or AXIR cells. **(C)** Venn diagram of specific and common genes expressed by 786-O-SUNR and AXIR cells. **(D)** Venn diagram of specific and common genes expressed by A498-SUNR and AXIR cells.** (E)** Venn diagram of specific and common genes expressed by 786-O-SUNR AXIR and A498-SUNR AXIR cells.

Comparative proteomic analysis revealed 695 upregulated proteins in SUNR and 690 in AXIR conditioned media compared to control cells, with 271 proteins common to both resistant phenotypes (Figure 1A, Table S1). This common secretome included previously identified AAT resistance factors, particularly ELR+CXCL cytokines (CXCL1, CXCL2, CXCL5, CXCL8) 31, interleukin 6 (IL-6) 19 and the pro-lymphangiogenic factor VEGFC 32, confirming our experimental approach. Among these factors, CTGF emerged as a protein of particular interest as it is known to be involved in fibrosis and lymphangiogenesis and may contribute to sarcomatoid features in ccRCC 33.

To elucidate the molecular mechanisms linking CTGF to AAT response and resistance, we performed transcriptomic analyses on two independent ccRCC cell lines, 786-O and A498. Heatmaps of modified activity patterns (MAPs) revealed only modest transcriptional changes in cells acutely treated with sunitinib or axitinib compared to controls, but substantial differences in cells with acquired resistance (SUNR and AXIR) (Figure 1B). In 786-O cells, we identified 4459 upregulated genes in SUNR and 5024 in AXIR cells, with 3510 genes commonly upregulated in both conditions (Figure 1C, Table S2). Similarly, A498 cells exhibited 3759 upregulated genes in SUNR and 4507 in AXIR cells, with 2115 shared genes (Figure 1D, Table S3).

Cross comparison of these commonly upregulated genes between the two cell lines revealed a set of 1107 genes induced across both models (Figure 1E, Table S4). Notably, CTGF ranked 116^th^ in this shared list and was the top-ranked secreted protein. Another member of the CCN family, CYR61 (CCN1), ranked 821^st^. These findings underscore the consistent overexpression of CTGF in resistant models and its known involvement in key tumor-promoting processes. However, its precise contribution to AAT resistance remains unclear, prompting further investigation into CTGF as a potential mediator of resistance, particularly given its secreted nature and capacity to influence the tumor microenvironment.

### CTGF contributes to ccRCC cell aggressiveness

**Figure 2:**
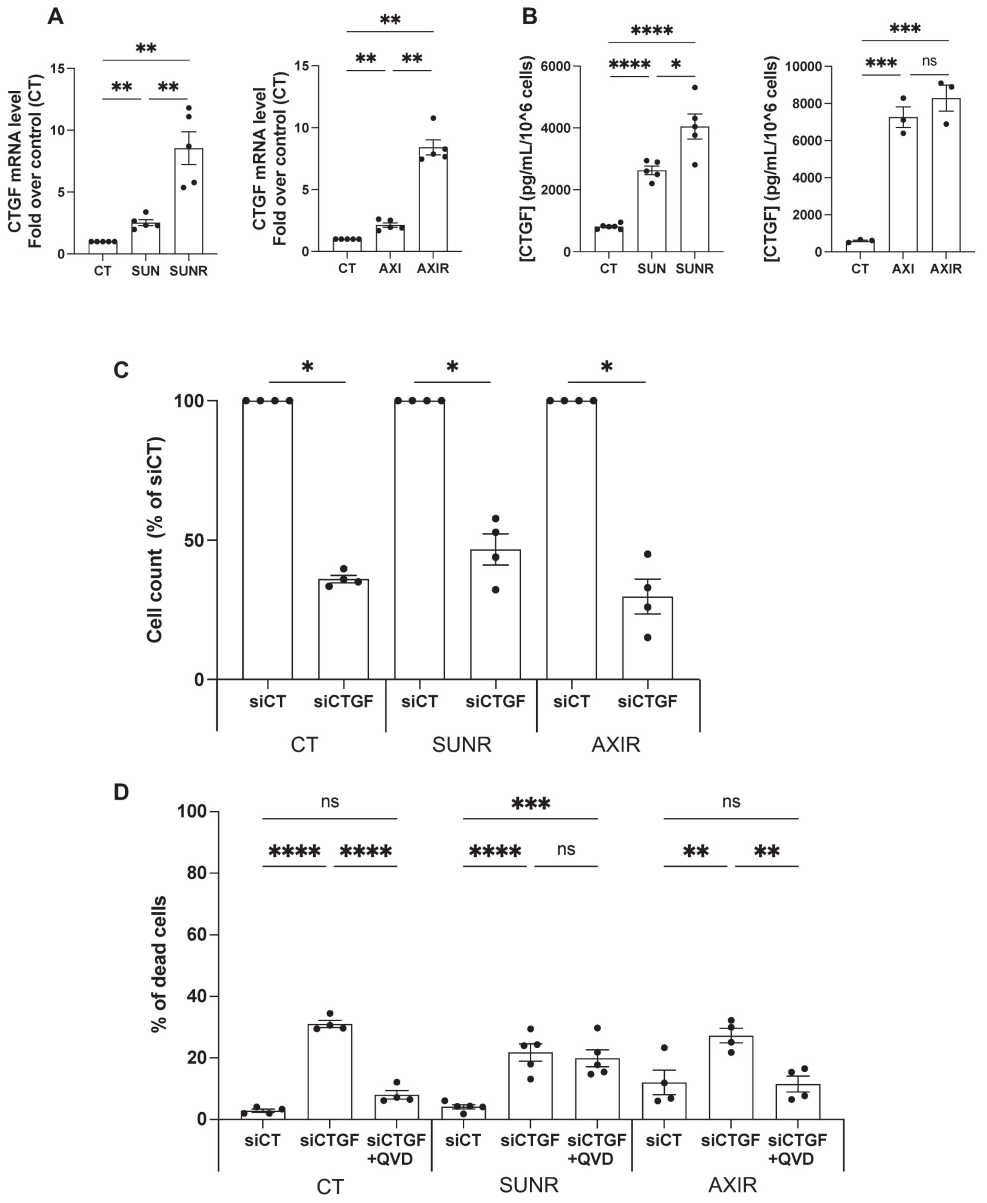
** Downregulation of CTGF by siRNA inhibits proliferation and induces cell death in ccRCC cells. (A)** *CTGF* mRNA levels in 786-O cells measured by RT-qPCR after 48 h under various conditions: control (CT), sunitinib-treated (2.5 µM) (SUN), sunitinib-resistant (SUNR), axitinib-treated (2.5 µM) (AXI), and axitinib-resistant (AXIR) cells. ** P < 0.01. **(B)** Levels of secreted CTGF in the supernatant of 786-O cells measured by ELISA after 96 h under various conditions: control (CT), sunitinib-treated (2.5 µM) (SUN), sunitinib-resistant (SUNR), axitinib-treated (2.5 µM) (AXI), and axitinib-resistant (AXIR) cells. * P < 0.05; *** P < 0.001; **** P < 0.0001; ns = not significant.** (C)** Effects of CTGF downregulation by siRNA, 96h after transfection, on the total number of cells, as measured by a Coulter counter, in control (CT), sunitinib-resistant (SUNR), and axitinib-resistant (AXIR) 786-O cells * P < 0.05. **(D)** The percentage of dead cells after treatment with siCT or siCTGF in the presence of the apoptosis inhibitor Q-VD-OPh (QVD) (10µM) was analyzed using propidium iodide (PI) and flow cytometry in CT, SUNR, and AXIR cells. ** P < 0.01; *** P < 0.001; **** P < 0.0001; ns = not significant.

To validate our proteomic and transcriptomic findings, we assessed CTGF expression at both the mRNA and protein levels in ccRCC cells. After 48 h of treatment, both sunitinib and axitinib increased *CTGF* mRNA levels, with a more pronounced elevation in SUNR and AXIR 786-O cells. CTGF protein levels also increased after 96 h of drug exposure and remained elevated in SUNR and AXIR cells. Although mRNA expression patterns were similar between the two resistant models, CTGF protein levels were significantly higher in the conditioned media of axitinib-treated and AXIR cells, suggesting a potential axitinib-specific post-transcriptional regulatory mechanism (**Figure 2A-B**). These findings were further confirmed in two additional ccRCC cell lines, A498 and RCC10 (**Figure S2A-C**).

**Figure 3:**
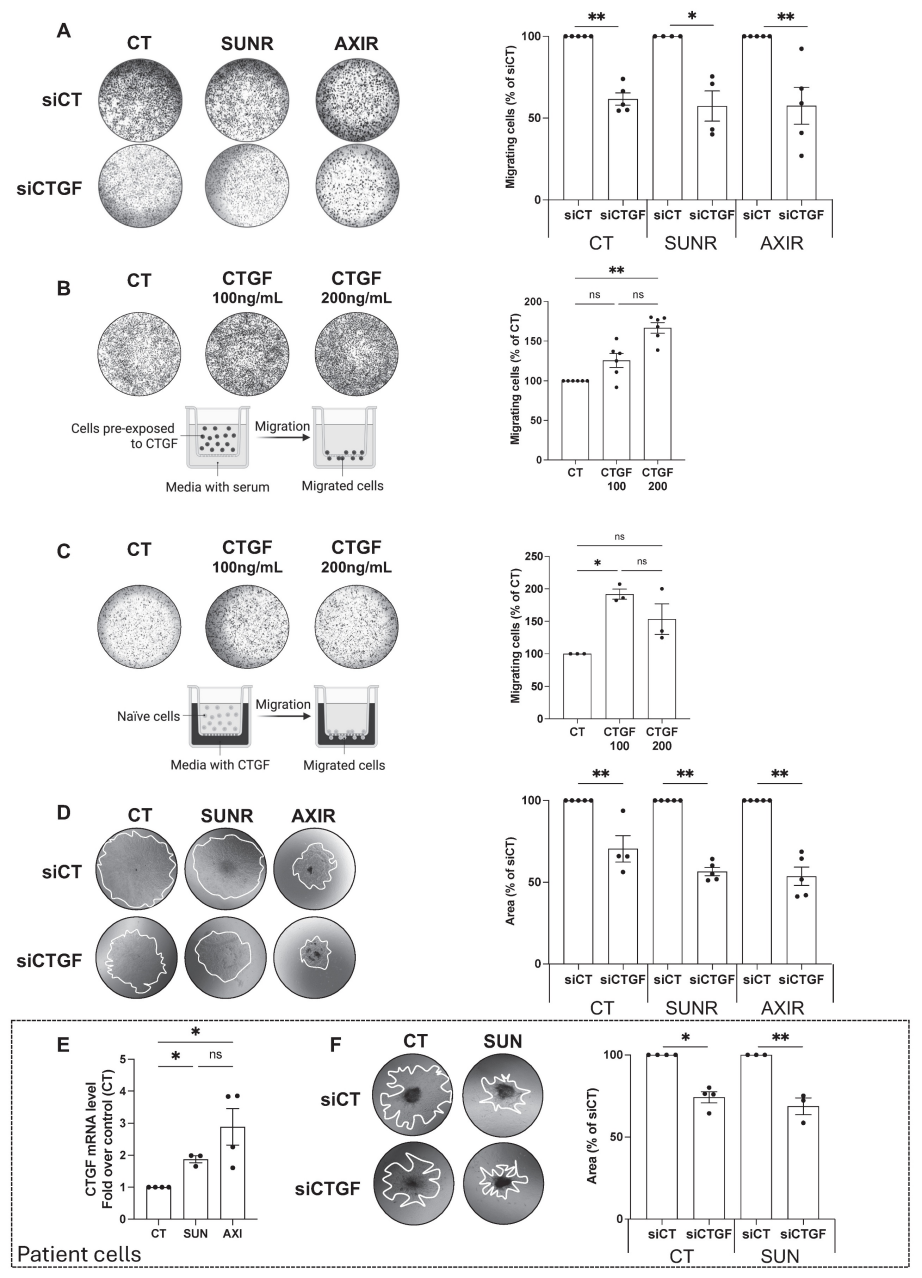
** CTGF drives tumor cell migration and invasion in ccRCC cell lines and patient samples. (A)** Migration of control (CT), sunitinib-resistant (SUNR), and axitinib-resistant (AXIR) 786-O cells after 48 h siRNA transfection. The migration assay was performed overnight and cells were attracted by serum. * P < 0.05; ** P < 0.01. **(B)** Migration of 786-O cells pre-exposed to increasing concentrations of CTGF for 48 h. A serum-driven overnight migration assay was performed. ** P < 0.01; ns = not significant. **(C)** Migration of 786-O cells toward a CTGF gradient (CTGF placed in the lower chamber in serum-free medium), and the migration assay was performed overnight. * P < 0.05; ns = not significant. **(D)** Invasion of CT, SUNR, and AXIR cells treated with either siCT or siCTGF, evaluated using tumor spheroids embedded in Matrigel. Invasion was quantified at day 5. ** P < 0.01. **(E)** *CTGF* mRNA levels measured by RT-qPCR in control (CT), sunitinib-treated (SUN) and axitinib-treated (AXI) cells freshly isolated from patients with metastatic renal cell carcinoma (ccRCC). * P < 0.05; ns = not significant. **(F)** Invasion of CT and SUN metastatic ccRCC patient cells treated with either control siRNA (siCT) or CTGF-targeting siRNA (siCTGF), evaluated using tumor spheroids embedded in Matrigel. * P < 0.05; ** P < 0.01. Representative images are shown for all panels.

Having established that CTGF is upregulated following AAT exposure, and even more so in AAT-resistant cells, we next investigated its mechanistic role in resistance by selectively silencing CTGF in both drug-naïve and resistant cell lines. A pool of siRNAs targeting CTGF was validated by demonstrating decreased *CTGF* mRNA expression and reduced intracellular and secreted protein levels (**Figure S3A-C**). CTGF knockdown significantly impaired the proliferation of parental 786-O cells and their SUNR and AXIR counterparts, compared to control siRNA-treated cells (siCT) (**Figure 2C**). This antiproliferative effect was primarily mediated by apoptosis, as evidenced by partial rescue with the pan-caspase inhibitor Q-VD-OPh. The similar extent of apoptosis across all cell types suggests that the reduction in cell number results from both diminished proliferation and increased cell death (**Figure 2D**). In addition to its effects on cell survival, CTGF also significantly influenced tumor cell aggressiveness, particularly their ability to migrate and invade. In Boyden chamber assays, naïve, SUNR and AXIR cells with control siRNA showed similar baseline migration, but CTGF knockdown significantly suppressed this ability (**Figure 3A**). To further demonstrate the migration-promoting role of CTGF, we either pretreated naïve cells with recombinant CTGF or used it as a chemoattractant. Pretreatment enhanced cell migration in a dose-dependent manner (**Figure 3B**), while CTGF showed saturating chemoattractant properties in the lower chamber at 100 ng/mL (**Figure 3C**). Elimination of CTGF also reduced invasion in spheroid assays (**Figure 3D**). The effects of CTGF invalidation by siRNA on cell proliferation, migration and invasion were confirmed in the RCC10 cell line (**Figure S4**).

To enhance the clinical relevance of our findings, we analyzed freshly isolated ccRCC specimens from the Nice University Hospital (CHU). Primary ccRCC cells treated with sunitinib or axitinib exhibited increased *CTGF* mRNA expression (**Figure 3E**), and CTGF knockdown significantly impaired spheroid invasion (**Figure 3F**). While we primarily used siRNA-based approaches to avoid potential compensatory effects associated with stable gene deletion, we validated our observations using CRISPR/Cas9-mediated CTGF knockout in 786-O cells (**Figure S5A**). CTGF-deficient cells displayed markedly reduced proliferation, diminished colony formation capacity (**Figure S5B-D**), and impaired migratory and invasive abilities (**Figure S5E-H**), confirming CTGF's essential role in both the proliferative and invasive behavior of ccRCC cells.

### YAP-mediated CTGF upregulation promotes resistance to AATs

**Figure 4:**
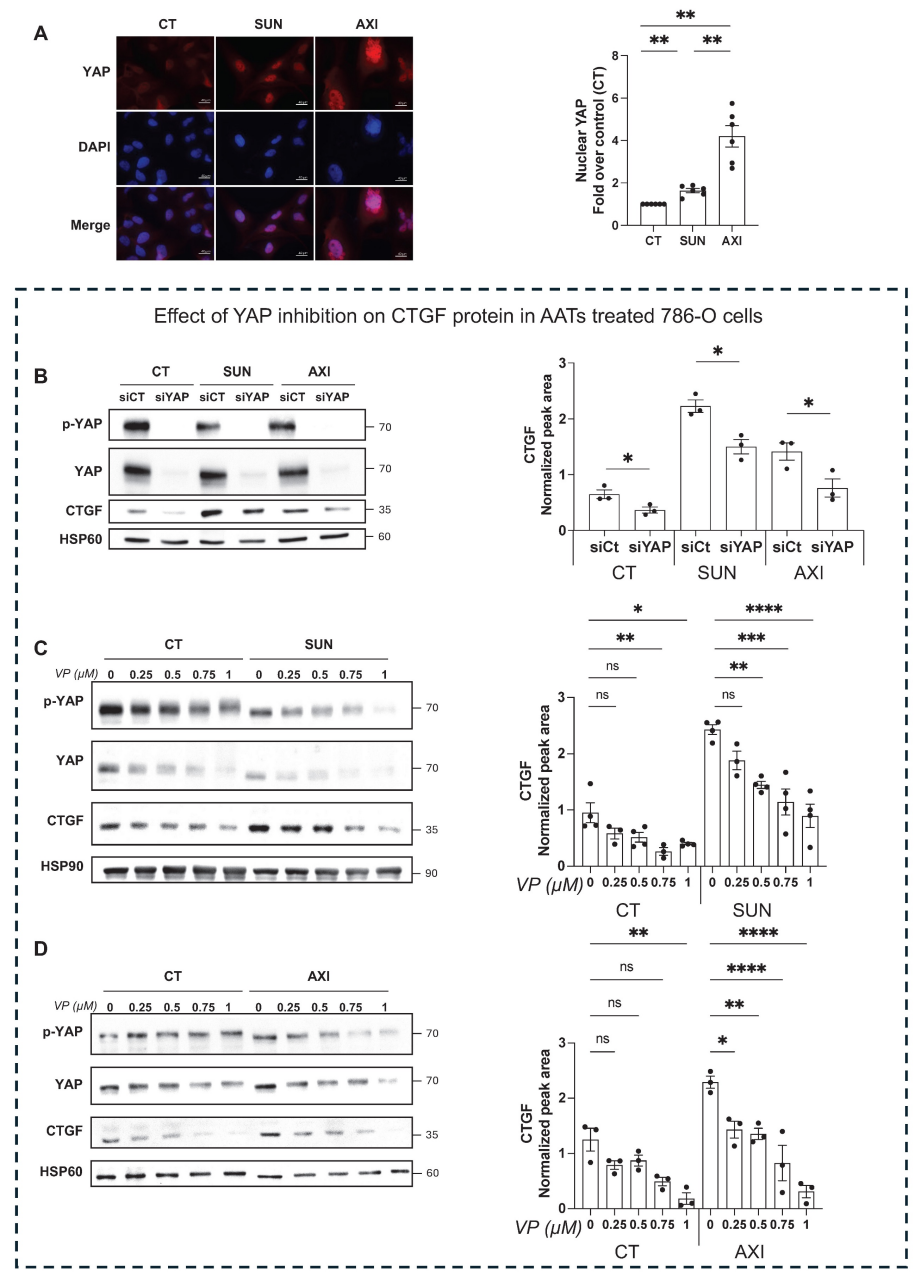
** Inhibition of YAP decreases CTGF expression and reduces AAT resistance of RCC cells. (A)** Image and quantification of nuclear YAP by immunofluorescence in control (CT), sunitinib-treated (2.5 µM) (SUN), and axitinib-treated (2.5 µM) (AXI) 786-O cells. ** P < 0.01 **(B)** Immunoblots analysis of protein expression in control (CT), sunitinib-treated (2.5µM) (SUN) or axitinib-treated (2.5µM) (AXI) 786-O cells transfected with siRNA (siCT) or YAP-targeted siRNA (siYAP) for 48 h, and the corresponding quantification of CTGF protein levels normalized to the loading control (HSP60). * P < 0.05. **(C)** Immunoblots analysis of protein expression in 786-O cells treated for 48 h with increasing concentration of the YAP inhibitor verteporfin (VP) (0-1µM), alone or in combination with sunitinib 2.5µM (SUN), and the corresponding quantification of CTGF protein levels normalized to the loading control (HSP90). ns = not significant; * P < 0.05; ** P < 0.01; *** P < 0.001; **** P < 0.0001. **(D)** Immunoblots analysis of protein expression in 786-O cells treated for 48 h with increasing concentration of the YAP inhibitor verteporfin (VP) (0-1µM), alone or in combination with axitinib 2.5µM (AXI), and the corresponding quantification of CTGF protein levels normalized to the loading control (HSP60). ns = not significant; * P < 0.05; ** P < 0.01; *** P < 0.001; **** P < 0.0001.

**Figure 5:**
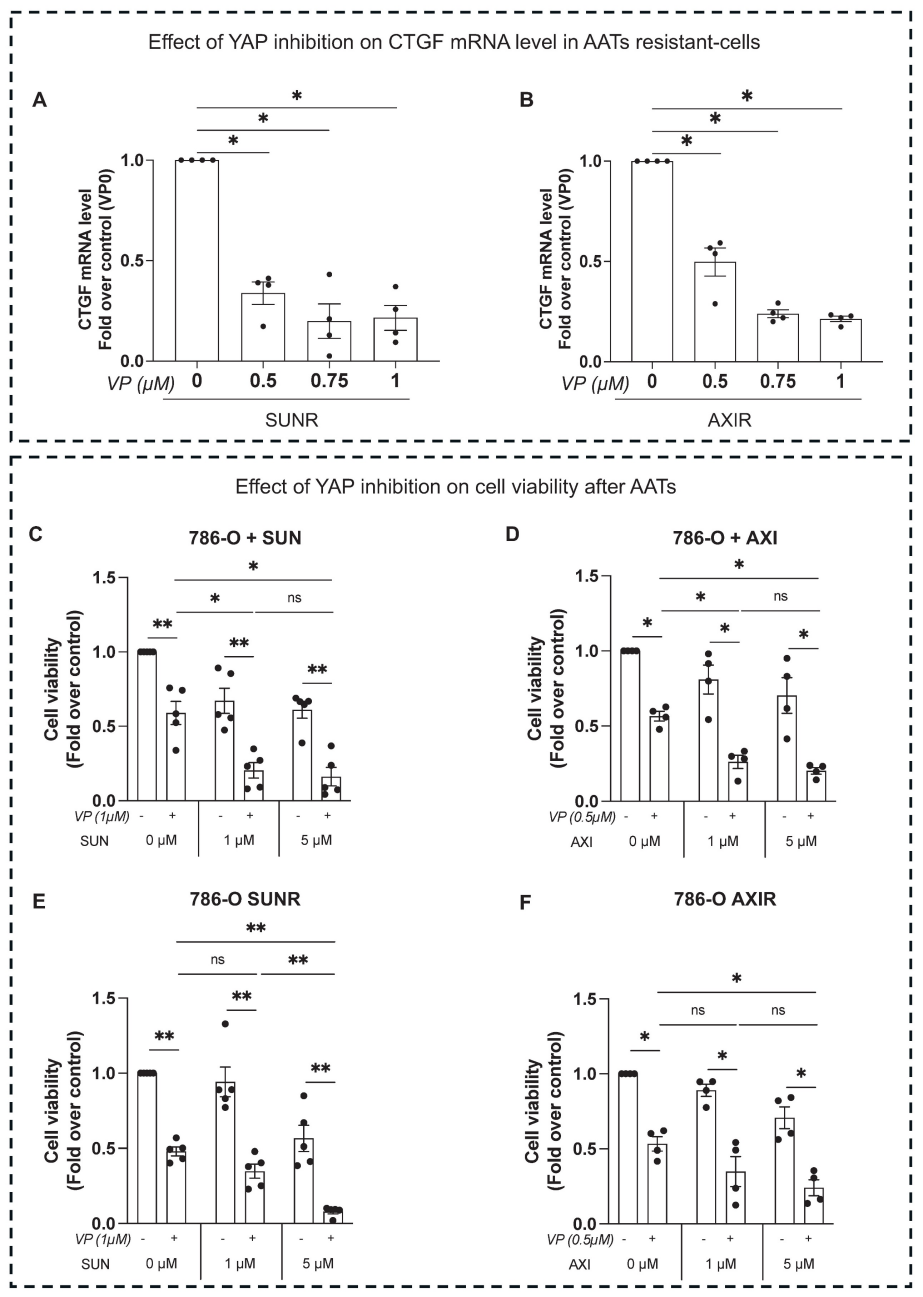
** Inhibition of YAP reduces AATs resistance of RCC cells. (A-B)** *CTGF* mRNA levels in 786-O sunitinib resistant (SUNR) **(A)** or axitinib resistant cells (AXIR) **(B)** measured by RT-qPCR after 48 h of treatment with increasing concentration of verteporfin (0-1µM). **(C-F)** Cell viability assessed using CellTiter-Glo after 24 h of treatment with verteporfin (VP) alone or in combination with increasing concentration of AATs. 786-O cells were treated either by sunitinib (0-5µM) **(C)** or by axitinib (0-5µM) **(D)**. 786-O sunitinib resistant (SUNR) **(E)** or axitinib resistant (AXIR)** (F)** cells were challenged with sunitinib or axitinib after verteporfin treatment. ns = not significant; * P < 0.05; ** P < 0.01; *** P < 0.001; **** P < 0.0001.

To investigate the molecular mechanism driving CTGF upregulation in response to sunitinib and axitinib, we examined the involvement of the YAP signaling pathway. Treatment with either drug resulted in a marked increase in YAP nuclear localization in 786-O cells (**Figure 4A**), indicating activation of YAP-mediated transcription. To confirm YAP's role in regulating CTGF expression, we demonstrated that both siRNA-mediated YAP knockdown (**Figure 4B**) and pharmacologic YAP inhibition by verteporfin, a selective inhibitor that disrupts YAP-TEAD interactions, significantly reduced CTGF protein levels in untreated, drug-treated (**Figure 4C-D**) and resistant cells (**Figure 5A-B**). Additionally, these findings establish YAP as a key transcriptional regulator of CTGF induction following anti-angiogenic drug exposure in ccRCC cells. Importantly, in sunitinib-resistant and axitinib-resistant ccRCC cells, combined treatment with verteporfin and sunitinib or axitinib resulted in a significant reduction in cell viability compared to AATs alone, as assessed by CellTiter-Glo assays (**Figure 5C-F**). This effect was not observed with AAT alone, indicating that YAP inhibition restores drug sensitivity in resistant cells. Together, these findings establish YAP as a key transcriptional regulator of CTGF induction following anti-angiogenic drug exposure and provide functional evidence that pharmacological targeting of the YAP-CTGF axis can overcome resistance to AAT in ccRCC cells.

### CTGF depletion suppresses tumor growth and metastasis in a zebrafish model

**Figure 6:**
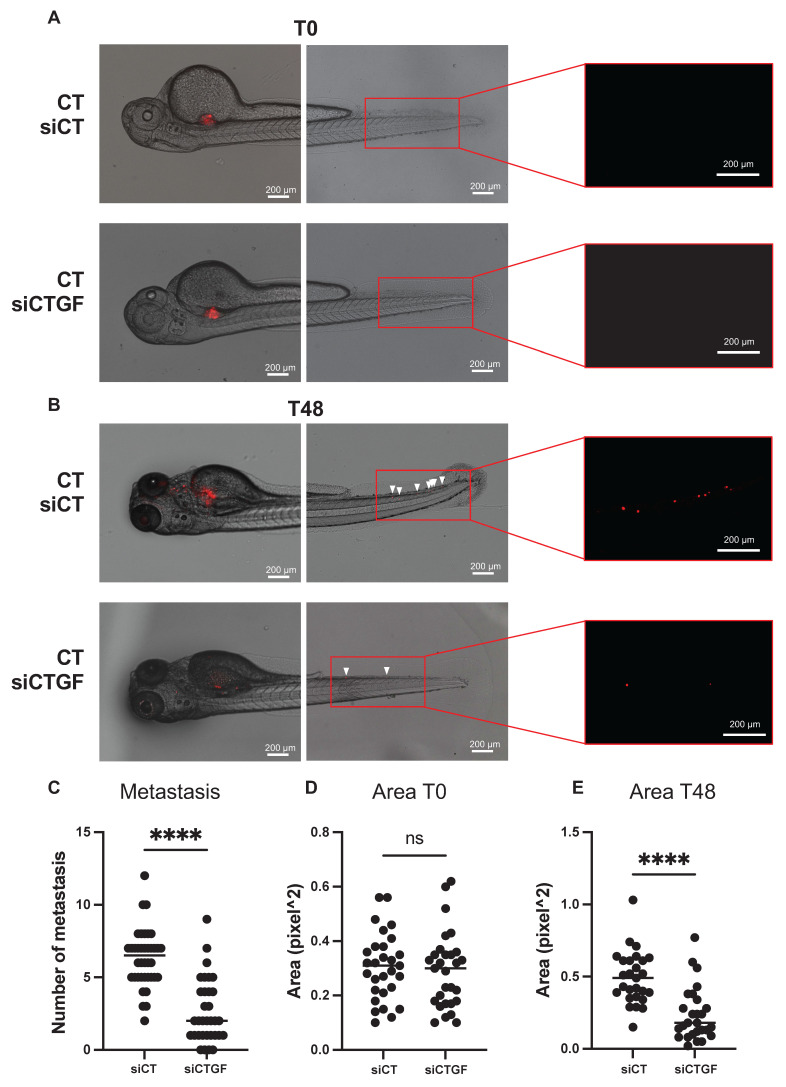
** Inhibition of CTGF by siRNA reduces local growth and distant metastasis in a zebrafish model. (A)** Representative image showing local and distant metastases. Zebrafish embryos (N = 30) were injected with 786-O cells treated with either control siRNA (siCT) or CTGF-targeting siRNA (siCTGF), labeled with red DiD, into the perivitelline space. Analyses were conducted at 0 h (T0) and 48 h post-injection (T48). **(C)** Quantification of distant metastases per zebrafish, based on fluorescent microscopy. **(D-E)** Quantification of tumor growth by measuring tumor area at the initial time point (T0) **(D)** and after 48 h (T48) **(E)**, along with the corresponding RFP signal area. ns = not significant; **** P < 0.0001.

**Figure 7:**
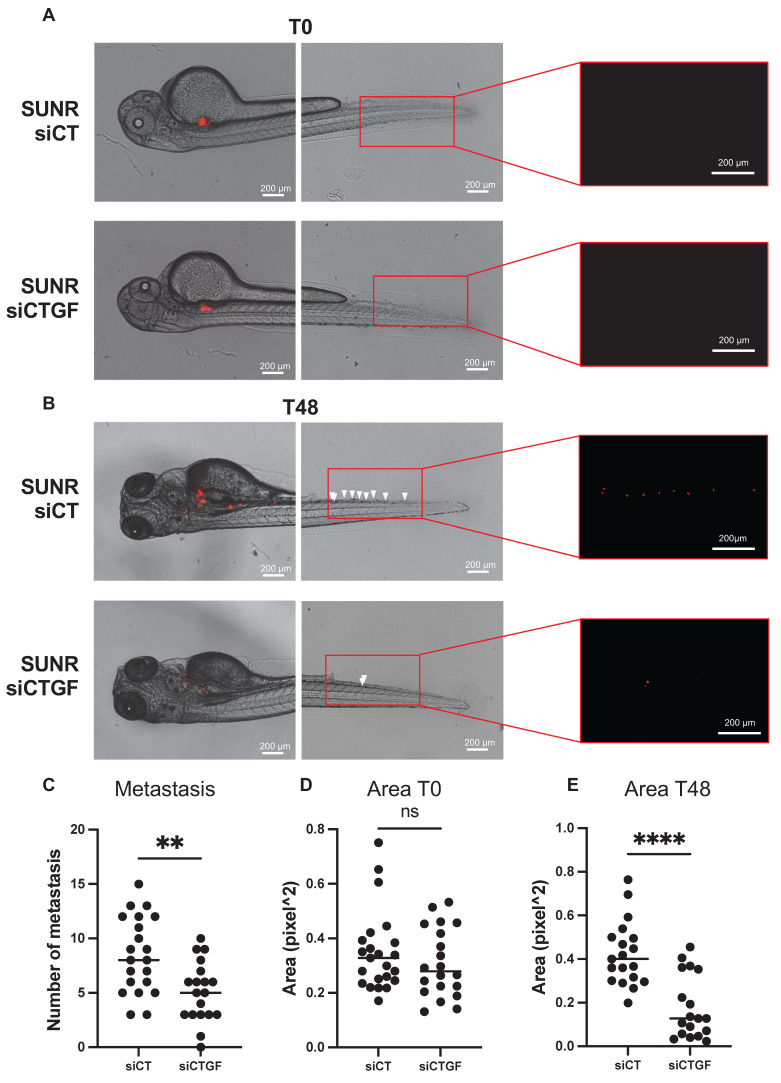
** Inhibition of CTGF by siRNA in sunitinib-resistant cells reduces local growth and distant metastasis in a zebrafish model. (A)** Representative image showing local and distant metastases. Zebrafish embryos (N = 20) were injected with 786-O sunitinib resistant cells treated with either control siRNA (siCT) or CTGF-targeting siRNA (siCTGF), labeled with red DiD, into the perivitelline space. Analyses were conducted at 0 h (T0) and 48 h post-injection (T48). **(C)** Quantification of distant metastases per zebrafish, based on fluorescent microscopy. **(D-E)** Quantification of tumor growth by measuring tumor area at the initial time point (T0) **(D)** and after 48 h (T48), along with **(E)** the corresponding RFP signal area. ns = not significant; ** P < 0.01; **** P < 0.0001.

To validate our *in vitro* findings in a physiologically relevant system, we used a zebrafish xenograft model to evaluate tumor growth and metastatic dissemination. This model allows quantitative tracking of tumor cell migration from the injection site to distal regions. Control and CTGF-depleted 786-O cells were labeled with the fluorescent dye DiI and injected into the perivitelline space (PVS) of 48-h post-fertilization (hpf) zebrafish embryos. Tumor burden was assessed at 2 days post-injection (dpi) by quantifying tumor area and RFP signal intensity. CTGF-silenced cells formed significantly smaller tumors than control cells (**Figure 6A-E**). Moreover, CTGF knockdown markedly reduced the number of metastatic tumor cells detected in the tail region (**Figure 6C**). These findings were corroborated using CRISPR/Cas9-mediated CTGF knockout cells (**Figure S6A-D**), reinforcing the pivotal role of CTGF in driving both tumor expansion and metastatic spread *in vivo*. Importantly, a similar reduction in tumor burden and metastatic dissemination were observed in sunitinib-resistant 786-O cells upon CTGF decrease by siRNA, demonstrating that the pro-tumorigenic role of CTGF is maintained in the resistant cells (**Figure 7A-E**).

### CTGF is a predictive biomarker for anti-angiogenic drug response

**Figure 8:**
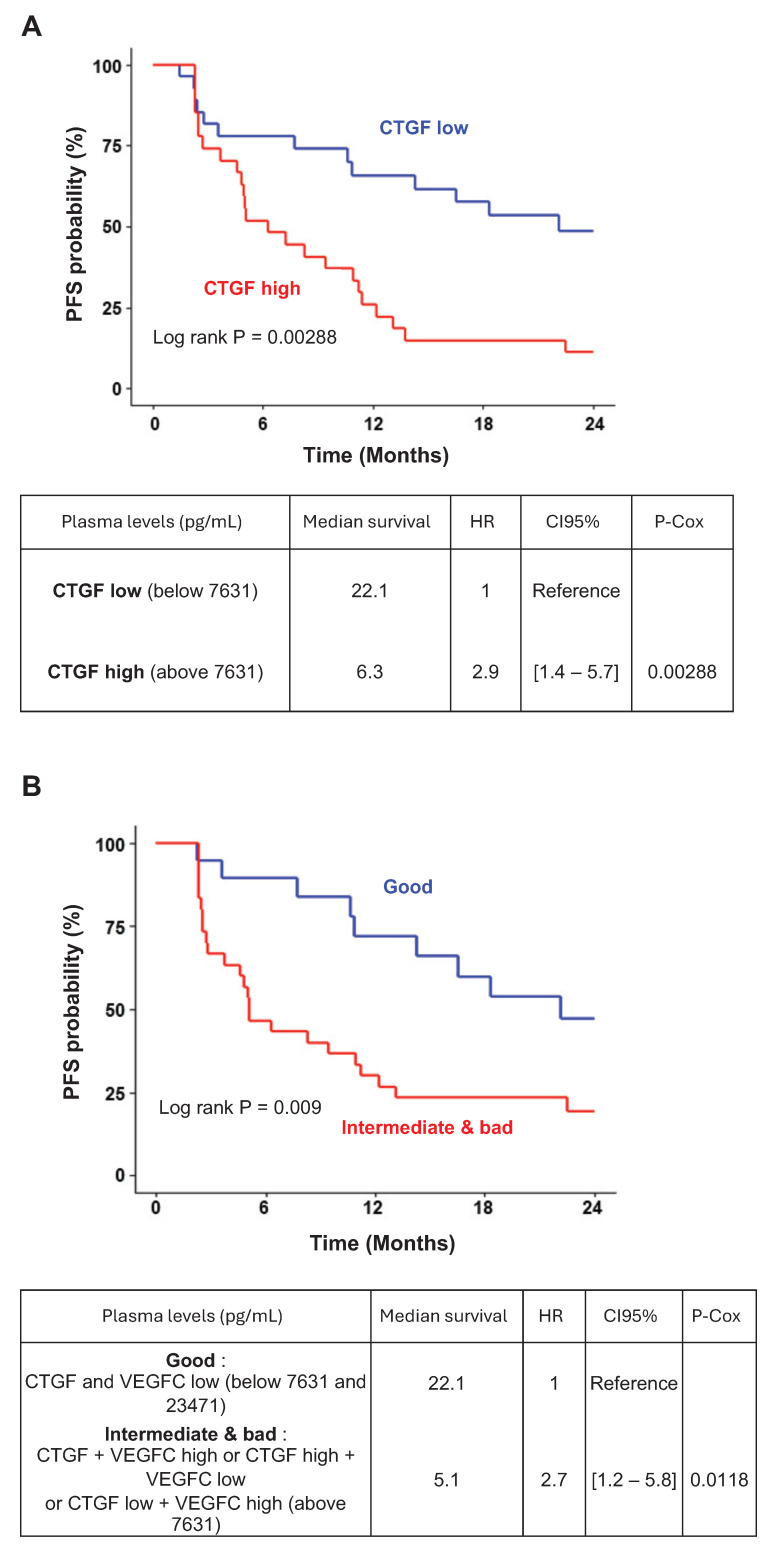
** CTGF and VEGFC as key indicators of susceptibility to AATs in ccRCC patients. (A)** Kaplan-Meier analysis of progression-free survival (PFS) in patients with ccRCC treated with AATs. PFS was calculated based on patient subgroups with plasma CTGF levels at diagnosis either below or above the cut-off value of 7631.16 pg/mL. Statistical significance (P-value), median progression-free time, hazard ratio (HR), and 95% confidence interval (CI) are provided. **(B)** Kaplan-Meier analysis of PFS in patients with ccRCC treated with AATs, based on plasma levels of both CTGF and VEGFC at diagnosis. PFS was calculated using subgroups with CTGF levels below or above 7631 pg/mL and VEGFC levels below or above 23471 pg/mL. Statistical significance (P-value), median progression-free time, HR, and 95% CI are provided.

To assess the clinical relevance of CTGF as a prognostic and predictive biomarker for response to AATs, we measured plasma CTGF levels in 56 patients with metastatic ccRCC (mccRCC) after primary tumor resection. These patients were enrolled in prospective clinical trials involving sunitinib, bevacizumab, or temsirolimus. Although CTGF levels did not significantly correlate with overall survival, they were strongly associated with progression-free survival (PFS). Patients with baseline CTGF levels below 7.631 ng/mL (n = 29) had a median PFS of 22.14 months, compared to 6.27 months in patients with higher levels (n = 27), corresponding to a hazard ratio of 2.9 [1.4-5.7], *P* = 0.00288 (**Figure 8A**). These findings suggest that elevated CTGF levels may predict reduced AAT efficacy.

Given prior evidence linking CTGF and VEGFC in mediating AAT resistance 34-36 and their co-induction in resistant cells, we also examined VEGFC levels in relation to clinical outcomes. Although patients with VEGFC levels above the optimal cutoff of 23.471 pg/mL showed a trend toward shorter PFS (hazard ratio: 1.4 [0.73-2.7], *P* = 0.13; **Figure S7A-B**), this association did not reach statistical significance.

To enhance predictive value, we stratified tumors based on combined CTGF and VEGFC expression profiles. Tumors with both markers above their respective cutoffs, or in the intermediate category (one high, one low), were associated with significantly shorter PFS than those with both markers below threshold values (**Figure 8B**). These results highlight the potential of combined CTGF and VEGFC plasma measurements to refine prognostic assessments and guide AAT selection in mccRCC.

## Discussion

Our research indicates that CTGF plays a crucial role in the aggressiveness and the development of resistance to AATs, particularly sunitinib and axitinib, in ccRCC. Using cell cultures, zebrafish models and patient-derived samples, our study is the first to explore the role of CTGF in ccRCC cells and highlight its significance in cancer progression. We found that CTGF promotes resistance by enhancing cancer cell survival, proliferation, migration and invasion.

Clinically, we also established a correlation between CTGF plasma levels and AAT response in ccRCC. Patients with elevated plasma CTGF levels had shorter PFS when treated with AATs, especially sunitinib. Our findings suggest two important clinical applications: CTGF as a biomarker to predict patient response to AATs in mccRCC, and as a target to potentially help overcome resistance to AATs.

CTGF, identified in 1991 in the endothelial vascular cell secretome 37, is a secreted protein belonging to the CCN family. It integrates pro-fibrotic TGF-β/SMAD signals and mechano-transduction via Hippo/YAP-TEAD, contributing to intratumoral fibrosis and shaping an immune-excluded tumor microenvironment 38-41. This remodeling can limit CD8+ T lymphocyte infiltration, enhance regulatory T cell proliferation, and reduce the efficacy of immune checkpoint inhibitors, creating a drug-tolerant environment 42-50. While our study did not directly evaluate cancer-associated fibroblasts or immune cells, our observations are consistent with previous findings in ccRCC and other fibrotic tumors, emphasizing the dual role of CTGF in modulating both tumor cell behavior and the surrounding stroma 51-54.

Beyond its effects on the microenvironment, CTGF acts as a direct driver of tumor cell survival. Consistent with these roles, silencing CTGF induced tumor cell death; however, the extent of rescue by pan-caspase inhibition varied depending on the resistance context: it was complete in AXIR cells, nearly complete in parental cells, and minimal in SUNR cells (**Figure 2D**). These observations suggest that CTGF loss primarily activates a caspase-dependent apoptotic program in parental and axitinib-resistant cells, whereas SUNR cells may depend on alternative, caspase-independent death mechanisms. Notably, inhibition of ferroptosis did not restore cell viability in any condition (**Figure S8**), arguing against a major role for ferroptotic cell death following CTGF silencing. Future investigations should aim to elucidate the mechanisms underlying the type of cell death of SUNR cells to CTGF depletion. Potential pathways to explore include necroptosis, pyroptosis, autophagic cell death, or mitotic catastrophe 55.

In parallel, our data identify YAP as a key upstream regulator of CTGF expression, whereas inhibition of NF-κB or p38-MAPK signaling did not affect CTGF levels (**Figure S9**), supporting a dominant role for YAP-dependent transcription under anti-angiogenic pressure.

Our results are consistent with published data in pancreatic adenocarcinoma 56, triple-negative breast cancer 15 and prostatic metastasis 57. Activation of the FAK/NF-kB pathway through the interaction of CTGF with integrin αvβ3 may be one mechanism by which CTGF induces tumor cell proliferation. The effect of CTGF on tumor cell migration has previously been described in other models such as melanoma and glioblastoma 58,59. CTGF may induce an epithelial-mesenchymal transition (EMT) phenotype in tumor cells, resulting in increased migratory capacity 60.

The observed functional role of CTGF has important implications for disease recurrence, both after remission in non-metastatic cases and following initial responses to AATs. Notably, although CTGF expression correlates with favorable prognosis in early-stage tumors, this duality echoes similar findings with VEGFC 61 and highlights the importance of disease context when considering CTGF as a therapeutic target. Based on our patient database analyses, we propose that therapeutic strategies targeting CTGF should be considered primarily in metastatic settings, potentially in combination with AATs. The relationship between CTGF expression and patient outcomes mirrors a pattern observed with vascular network mediators, including VEGFA and VEGFC. This variable prognostic association may be due to the dual nature of tumor vasculature in cancer progression 34,35. In early tumor stages, when immune function remains intact, well-developed lymphatic and/or vascular networks enhance anti-tumor immunity by facilitating immune cell infiltration. Conversely, in advanced tumor stages characterized by immune cell depletion, these same vascular networks primarily promote metastatic dissemination. This context-dependent functionality underscores the importance of considering disease stage and immune status when developing therapeutic strategies targeting vascular mediators.

Overall, these insights open new avenues for therapeutic exploration. Combining AATs, immune checkpoint inhibitors, and CTGF-targeted therapies represents a promising strategy. Our results also highlight the relevance of CTGF in modulating both tumor cell behavior and immune dynamics, reinforcing its candidacy as a therapeutic and biomarker target. Given the strong association of CTGF with treatment response and clinical outcome, integrating it into biomarker-driven treatment strategies could enhance patient stratification and therapeutic precision.

In conclusion, our findings position CTGF as a pivotal factor in ccRCC progression and resistance. Its therapeutic value lies in careful, context-aware targeting, particularly in advanced disease, where it may complement existing modalities to improve treatment outcomes in renal cancer.

## Supplementary Material

Supplementary figures and tables.

## Abbreviations

AATs: Antiangiogenic Therapies
AXI: Axitinib
AXIR: Axitinib-Resistant
CCN: Cyr61, CTGF, Nov
ccRCC: Clear Cell Renal Cell Carcinoma
CHU: Centre Hospitalier Universitaire
CT: Control
CTGF: Connective Tissue Growth Factor
CXCL: C-X-C motif chemokine ligand
DMSO: Dimethyl Sulfoxide
Dpi: Days Post-Injection
ECL: Enhanced Chemiluminescence
ECM: Extracellular Matrix
ELISA: Enzyme-Linked Immunosorbent Assay
EMT: Epithelial-Mesenchymal Transition
FBS: Fetal Bovine Serum
GFP: Green Fluorescent Protein
hpf: Hours Post-Fertilization
ITF: Intratumoral Fibrosis
KO: Knockout
MAPK: Mitogen-Activated Protein Kinase
mccRCC: Metastatic ccRCC
NF-κB: Nuclear Factor κ-light-chain-enhancer of activated B cells
OS: Overall Survival
PFS: Progression-Free Survival
PI: Propidium Iodide
PVS: Perivitelline Space
RFP: Red Fluorescent Protein
RNA-Seq: RNA sequencing
SEM: Standard Error of the Mean
siRNA: small interfering RNA
SUN: Sunitinib
SUNR: Sunitinib-Resistant
TCGA: The Cancer Genome Atlas
TGF-β: Transforming Growth Factor β
TKI: Tyrosine Kinase Inhibitor
TME: Tumor Microenvironment
VEGFA: Vascular Endothelial Growth Factor A
VEGFC: Vascular Endothelial Growth Factor C
VEGFR: Vascular Endothelial Growth Factor Receptor
YAP: Yes-Associated Protein
