# Supplementary material for: Connective tissue growth factor contributes to resistance to anti-angiogenic therapies in renal cancer
Source: Theranostics. 2026 Feb 11;16(9):4489–507. doi: 10.7150/thno.125269 (PMC12964018; doi:10.7150/thno.125269)
Supplement: Supplementary file 1 — Supplementary figures and tables. [file thnov16p4489s1.pdf]

A

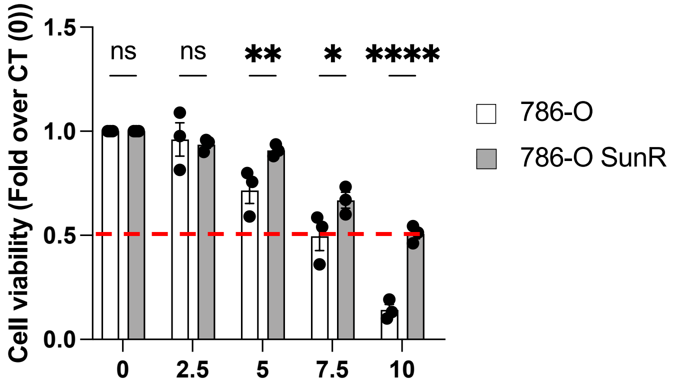

B

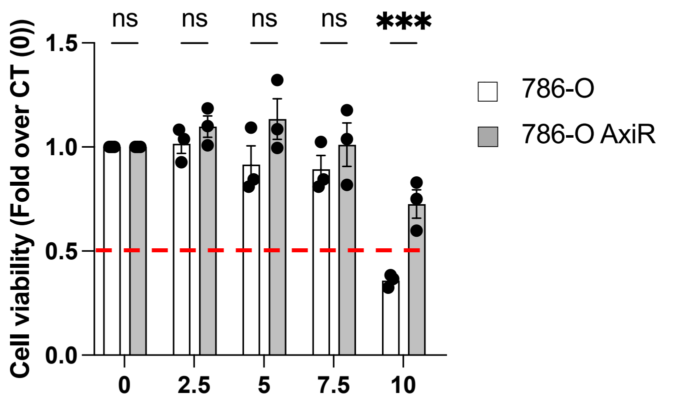

Figure S1: Teisseire, M *et al*

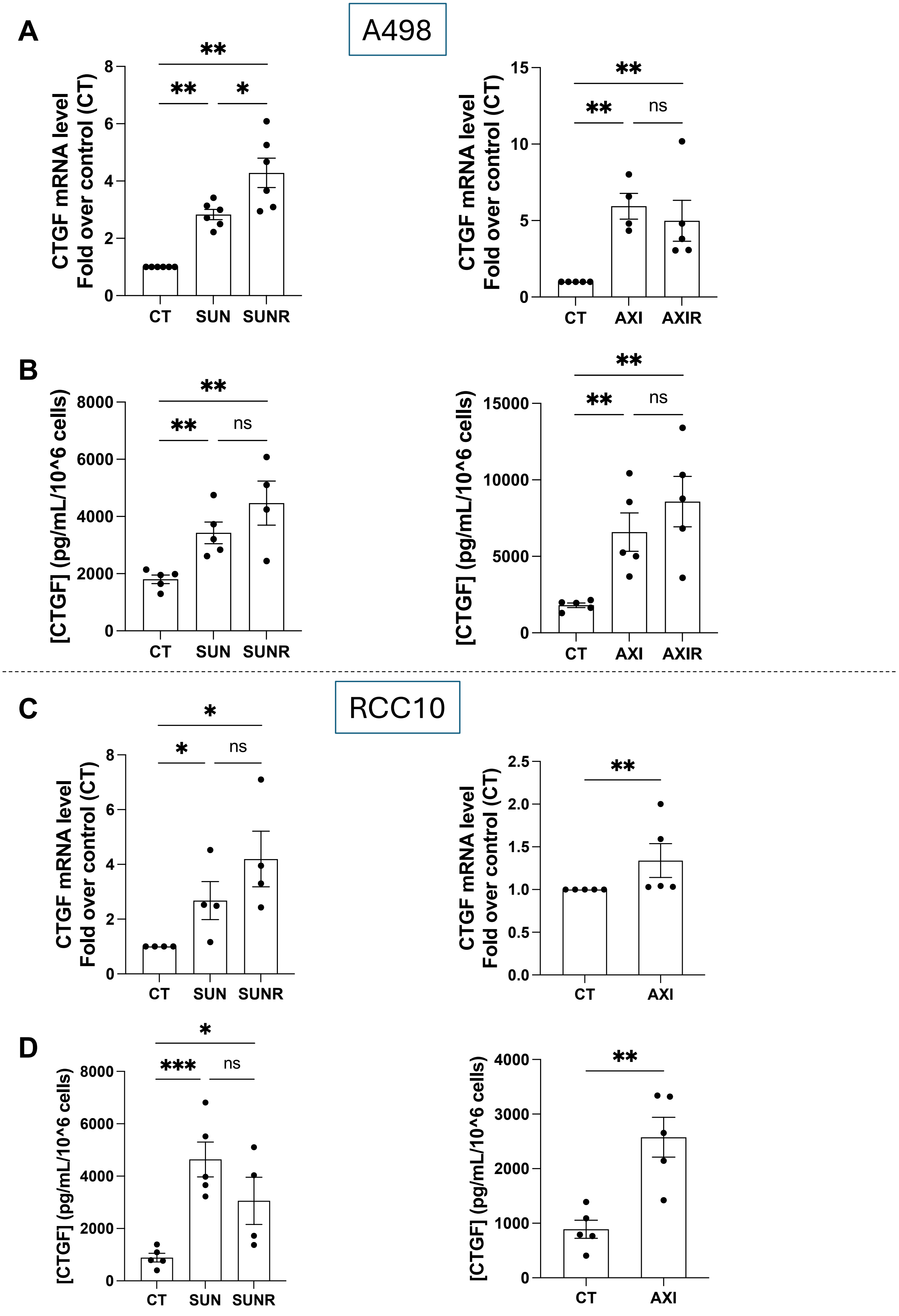

Figure S2: Teisseire, M *et al*

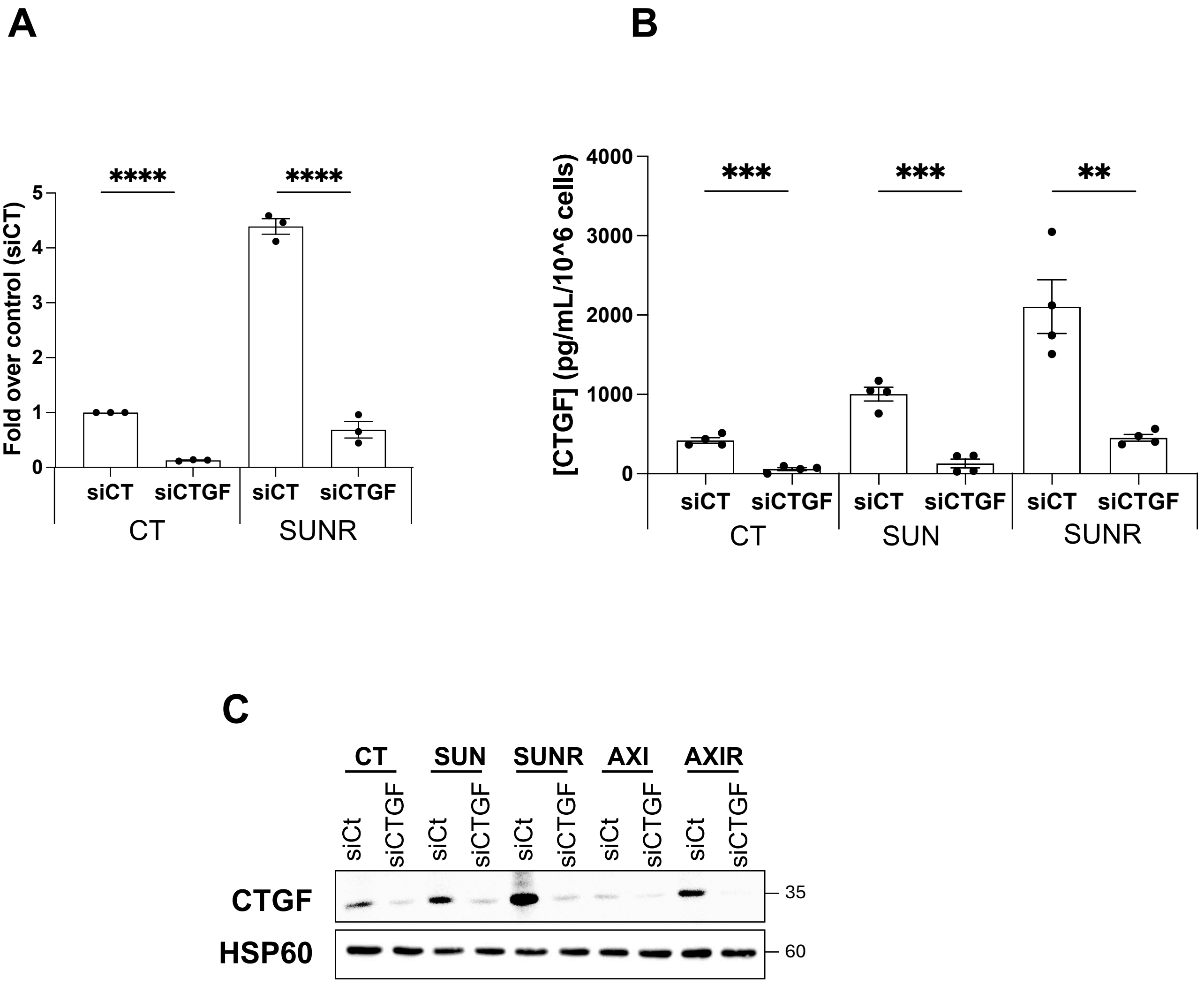

Figure S3: Teisseire, M *et al*

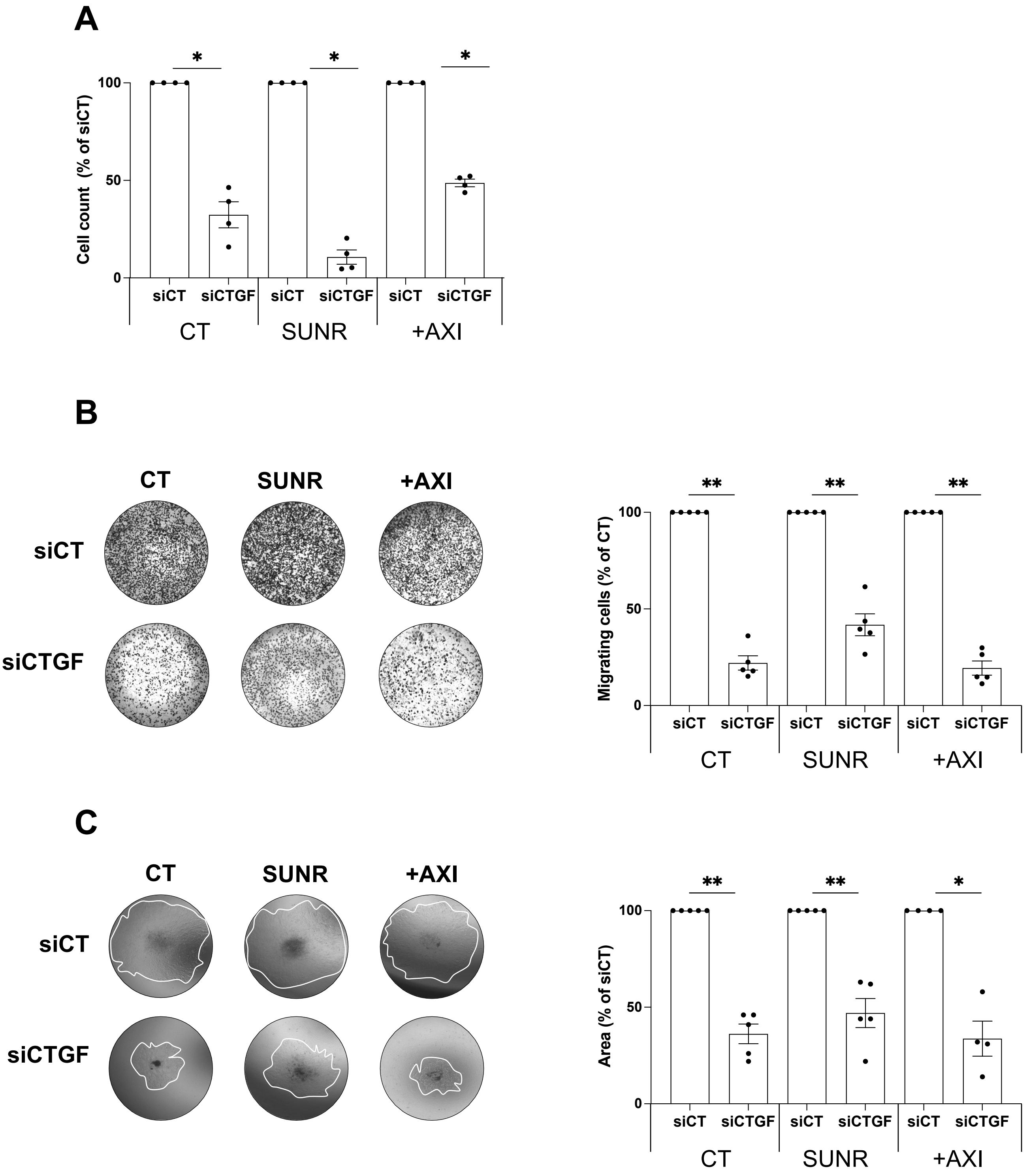

Figure S4: Teisseire, M *et al*

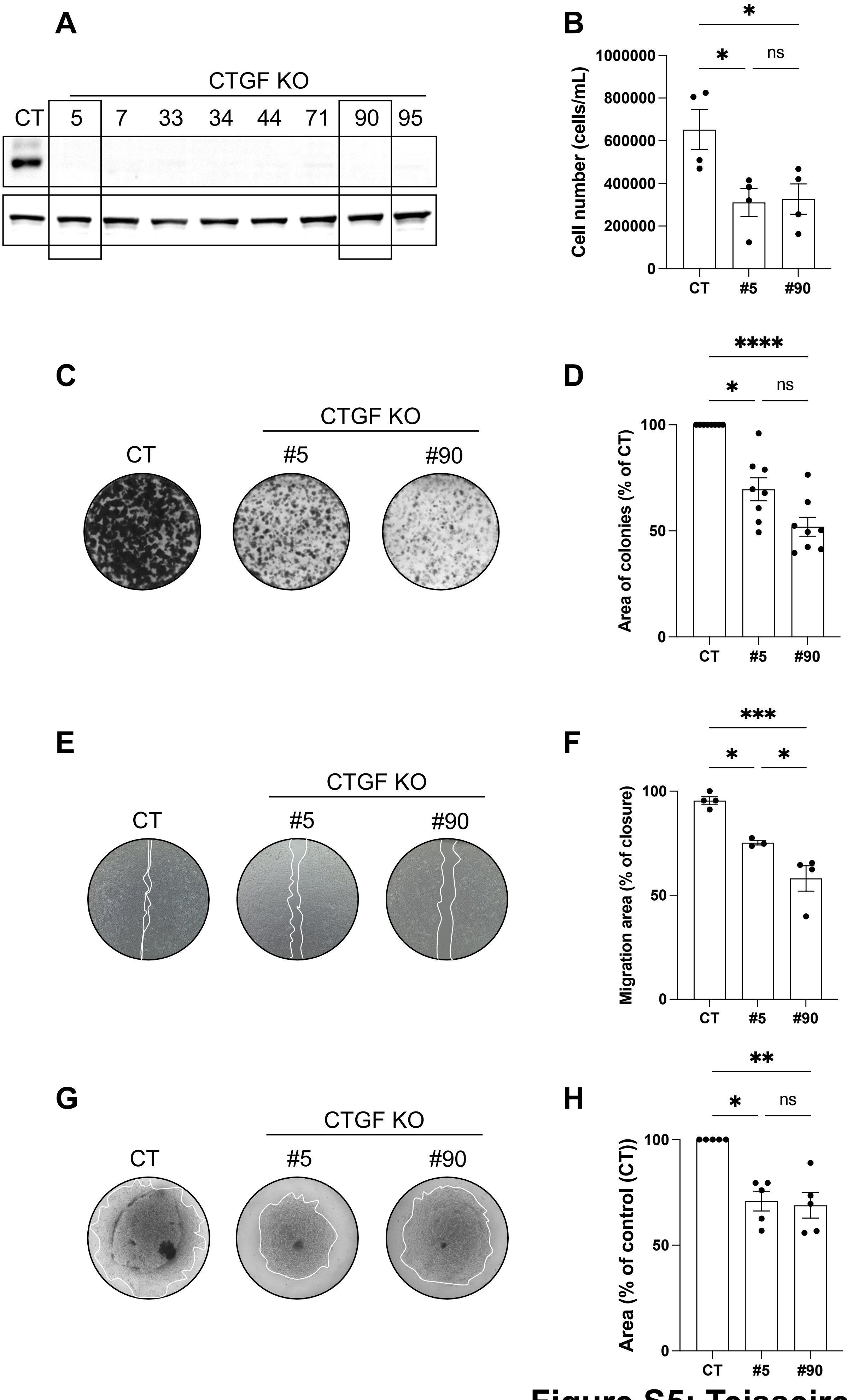

Figure S5: Teisseire, M *et al*

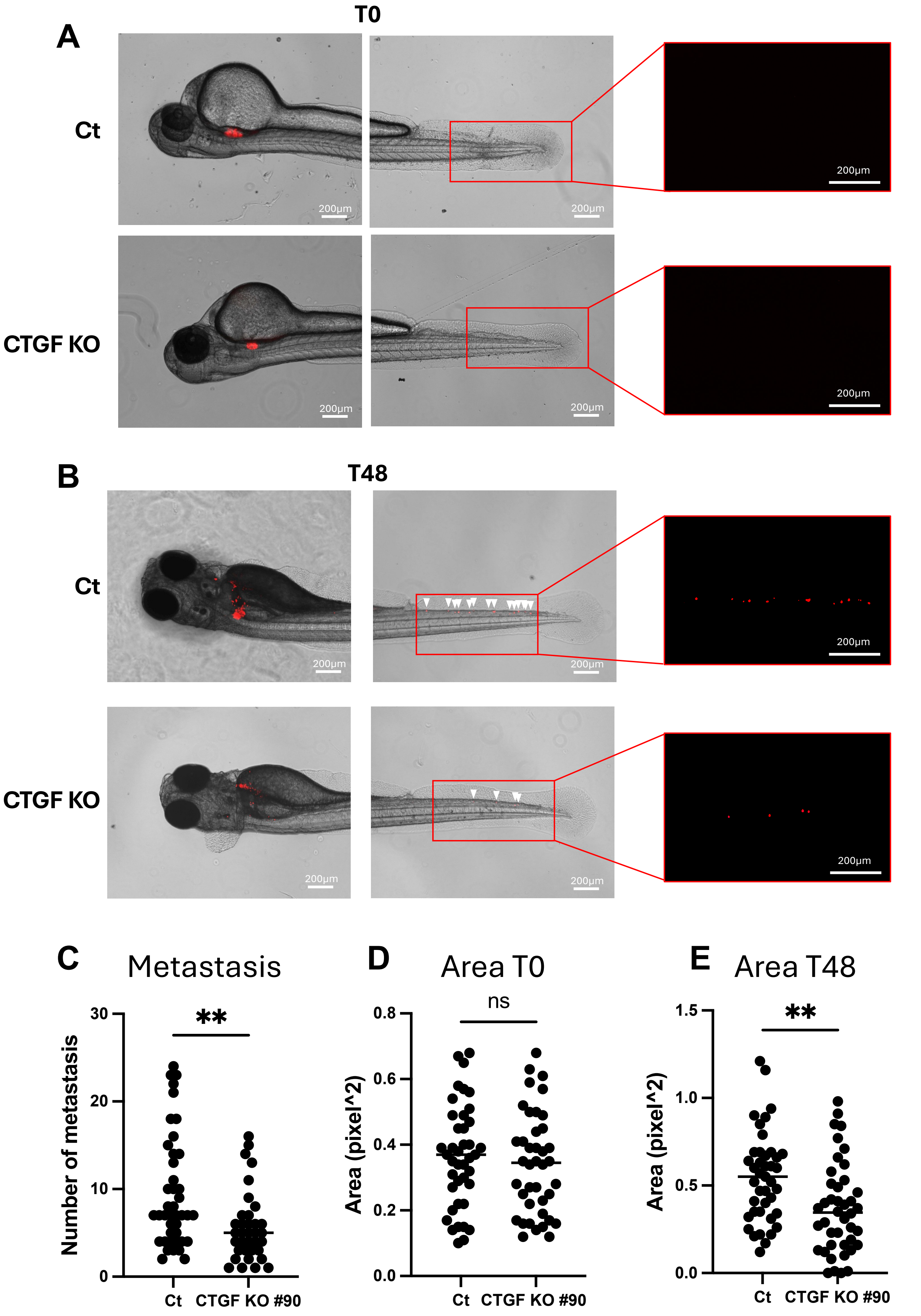

Figure S6: Teisseire, M *et al*

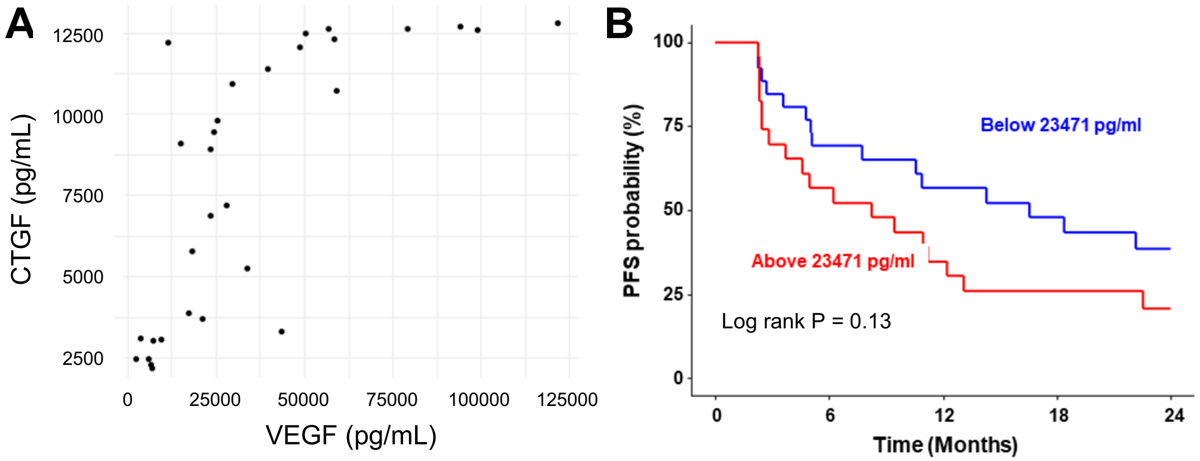

Plasma levels (pg/mL)	Median survival	HR	CI95%	P-Cox
**VEGFC low** (below 23471)	16.6	1	Reference	
**VEGFC high** (above 23471)	8.3	1.4	[0.73 – 2.7]	0.3

Figure S7: Teisseire, M *et al*

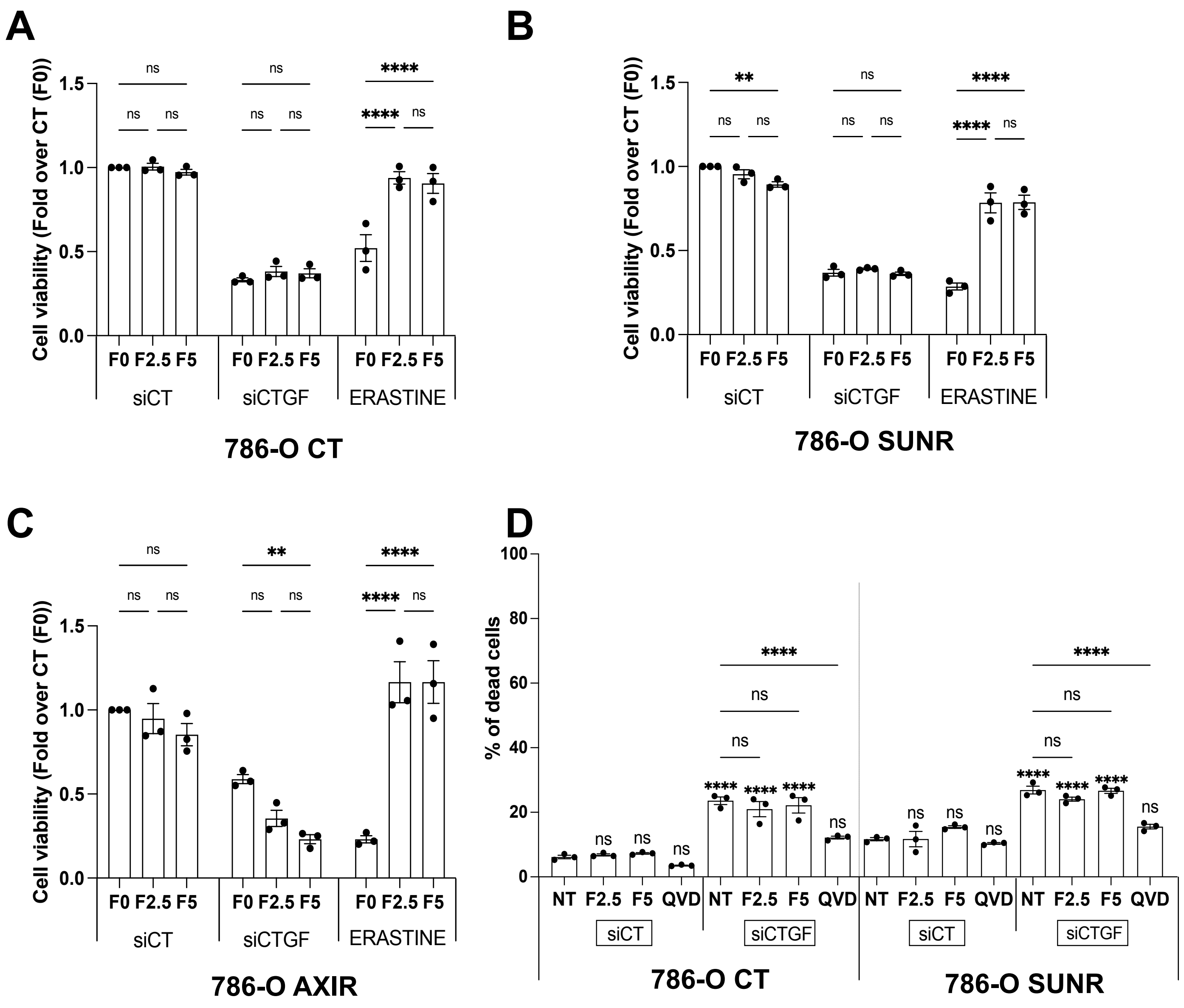

Figure S8: Teisseire, M *et al*

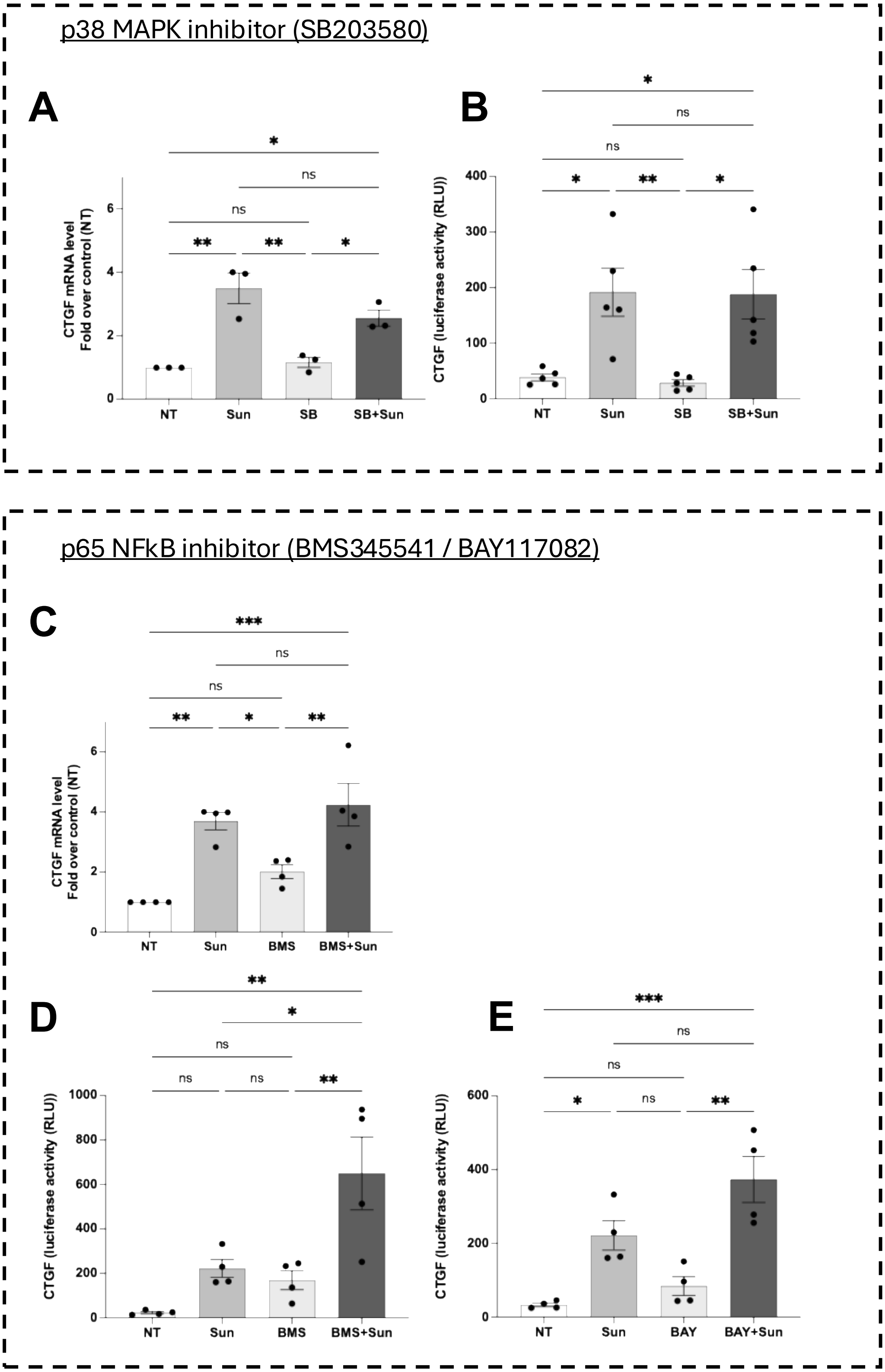

Figure S9: Teisseire, M *et al*

Supplementary informations

Supplementary Figure S1: Dose-response effect of sunitinib and axitinib on parental and resistant 786-O cell viability. Parental 786-O and sunitinib (SUNR) **(A)** or axitinib (AXIR) **(B)** resistant cells were treated for 24h with increasing concentration of sunitinib (0-10µM) **(A)** or axitinib (0-10µM) **(B)**. Cell viability was quantified using CellTiter-Glo assay.

Supplementary Figure S2: CTGF levels are elevated in sunitinib and axitinib treated and resistant ccRCC cells (A498 and RCC10). (A) CTGF mRNA levels measured by RT-qPCR after 48 hours in control (CT), sunitinib-treated (SUN), axitinib-treated (AXI), and sunitinib-resistant (SUNR) or axitinib-resistant (AXIR) A498 ccRCC cells. * P < 0.05; ** P < 0.01; ns = not significant. **(B)** Secreted CTGF levels measured by ELISA after 96 hours in control (CT), sunitinib-treated (SUN), axitinib-treated (AXI), and sunitinib-resistant (SUNR) or axitinib-resistant (AXIR) A498 ccRCC cells. ** P < 0.01; ns = not significant. **(C)** CTGF mRNA levels measured by RT-qPCR after 48 hours in control (CT), sunitinib-treated (SUN), axitinib-treated (AXI), and sunitinib-resistant (SUNR) RCC10 ccRCC cells. * P < 0.05; ** P < 0.01; ns = not significant. **(D)** Secreted CTGF levels measured by ELISA after 96 hours in control (CT), sunitinib-treated (SUN), axitinib-treated (AXI), and sunitinib-resistant (SUNR) RCC10 ccRCC cells. * P < 0.05; ** P < 0.01; *** P < 0.001; ns = not significant.

Supplementary Figure S3: Efficacy of siCTGF Treatment. (A) CTGF mRNA levels measured by RT-qPCR after 48 hours of transfection with either control siRNA (siCT) or CTGF-targeting siRNA (siCTGF) in control (CT) and sunitinib-resistant (SUNR) 786-O cells. **** P < 0.0001 **(B)** Secreted CTGF levels measured by ELISA after 48 hours of transfection with siCT or siCTGF in control (CT), sunitinib-treated (SUN), and sunitinib-resistant (SUNR) 786-O cells. ** P < 0.01; *** P < 0.001. **(C)** Protein expression profiles analyzed by

immunoblotting of CT, SUN, SUNR, axitinib-treated (AXI), and axitinib-resistant (AXIR) 786-O cells transfected with either siCT or siCTGF for 48 hours.

Supplementary Figure S4: CTGF drives tumor cell proliferation, migration and invasion in RCC10. (A) Effects of CTGF downregulation by siRNA on the total number of cells, as measured by a Coulter counter, in control (CT), sunitinib-resistant (SUNR), and axitinib-treated (AXI) RCC10 cells, 96 hours post-transfection. * $P < 0.05$. **(B)** Effects of siCT or siCTGF after 48 hours post-transfection on the migration of control (CT), sunitinib-resistant (SUNR), and axitinib-treated (+AXI) RCC10 cells, assessed using Boyden chamber assays. Representative images of Boyden chambers are shown. ** $P < 0.01$. **(C)** The invasive properties of control (CT), sunitinib-resistant (SUNR), and axitinib-treated (+AXI) RCC10 cells, treated with either siCT or siCTGF were evaluated using tumor spheroids embedded in Matrigel. Representative images are shown * $P < 0.05$;** $P < 0.01$.

Supplementary Figure S5: CTGF Invalidation using CRISPR-Cas9 technology. (A) CTGF expression analyzed by immunoblotting in control (CT) and CTGF knockout (KO) clones #5 and #90. **(B)** Cell count of control (CT) and CTGF KO (#5 and #90) cells after 96 hours of growth. ns = not significant; * $P < 0.05$ **(C-D)** Clonogenic assay results. **(C)** Representative images of the clonogenic assay showing colony formation for control (CT) and CTGF KO clones (#5 and #90) and **(D)** area of colonies measured for control (CT) and CTGF KO clones (#5 and #90) ns = not significant; * $P < 0.05$; **** $P < 0.0001$. **(E-F)** Effects of CTGF KO on cell migration assessed by scratch assays. **(E)** Representative images of the scratch assays are shown and **(F)** migration area representing the percentage of wound closure. ns = not significant; ** $P < 0.01$. **(G-H)** Effects of CTGF KO on cell invasion. **(G)** Representative

images of spheroids embedded in matrigel are shown **(H).** Invasion area is represented as a percentage of the control (CT). ns = not significant; * P < 0.05; ** P < 0.01.

Supplementary Figure S6: CTGF invalidation by CRISPR-Cas9 decreases local growth and distant metastasis in a zebrafish model. (A) Representative image showing local and distant metastases. Zebrafish embryos (N = 35) were injected with 786-O cells treated with either control cells (Ct) or CTGF KO cells (CTGF KO), labeled with red DiD, into the perivitelline space. Analysis was conducted at 0 hours (T0) and 48 hours post-injection (T48). **(C)** Quantification of distant metastases per zebrafish, based on fluorescent miscroscopy. **(D-E)** Quantification of tumor growth by measuring tumor area at the initial time point (T0) **(D)** and after 48 hours (T48) **(E)**, along with the corresponding RFP signal area. ns = not significant; ** P < 0.01.

Supplementary Figure S7: The expression of CTGF and VEGFC is interdependent. (A) Logarithmic correlation curve showing the relationship between CTGF and VEGFC levels. The curve illustrates the degree of correlation between these two markers. **(B)** Kaplan–Meier analysis of progression-free survival (PFS) in patients with clear cell renal carcinoma (KIRC). PFS was calculated based on patient subgroups with plasma levels of VEGFC either below or above an optimal cut-off value. P-values and median survival times are provided.

Supplementary Figure S8: Ferrostatin-1 effect on cell viability and cell death following 96h siRNA transfection in 786-O cells. (A-C) Cell viability mesured using CellTiter-Glo assay 96h after siRNA transfection (siCT or siCTGF) or after treatment with erastin (2.5µM), either alone (F0) or in combination with ferrostatin-1 (2.5µM (F2.5) or 5µM (F5)) a, ferroptosis inhibitor. Luminescence values are normalized to the siCT F0 condition. Panels correspond to

786-O cells (CT) **(A)**, sunitinib resistant 786-O cells (SUNR) **(B)**, and axitinib resistant 786-O cells (AXIR) **(C). (D)** Percentage of dead cells measured 96h after siRNA transfection (siCT or siCTGF) in the presence of ferrostatin-1 (2.5µM (F2.5) or 5µM (F5)) or the pan-caspase inhibitor Q-VD-OPh (QVD, 10µM). Cell death was analyzed by propidium iodide staining using flow cytometry in CT and SUNR 786-O cells. ** P < 0.01; *** P < 0.001; *** P < 0.0001; ns = non-significant.

Supplementary Figure S9: Sunitinib-induced CTGF upregulation is not reversed by MAPK or NFkB inhibitors. (A-B) Effect of p38 MAPK inhibitor SB203580. **(A)** CTGF mRNA levels mesured by RT-qPCR after 48h of treatment with sunitnib 2.5µM (Sun), SB203580 10µM (SB) alone, or in combination (SB+Sun). **(B)** CTGF luciferase activity (RLU, Relative Luciferase Units) mesured after 48h of same treatment. **(C-E)** Effect of NFkB inhibitors BMS345541 and BAY117082. **(C)** CTGF mRNA level mesured by RT-qPCR after 48h of treatment with sunitnib 2.5µM (Sun), BMS345541 2.5µM (BMS) alone, or in combination (BMS+Sun). **(D-E)** CTGF luciferase activity after 48h of treatment with sunitnib 2.5µM (Sun), BMS345541 2.5µM (BMS) or BAY117082 2.5µM (BAY) alone, or in combination.

Supplementary Table S1: Common Proteins Upregulated in SUNR and AXIR Conditioned Media.

This table lists proteins that are commonly upregulated in the conditioned media of sunitinib-resistant (SUNR) and axitinib-resistant (AXIR) ccRCC cells.

Supplementary Table S2: Common Genes Upregulated in 786-O SUNR and AXIR Cells.

This table identifies genes that are commonly upregulated in 786-O ccRCC cells resistant to sunitinib (SUNR) and axitinib (AXIR).

Supplementary Table S3: Common Genes Upregulated in A498 SUNR and AXIR Cells.

This table details genes that are commonly upregulated in A498 ccRCC cells resistant to sunitinib (SUNR) and axitinib (AXIR).

Supplementary Table S4: Common Genes Upregulated in 786-O SUNR and AXIR, and A498 SUNR and AXIR Cells.

This table presents genes that are commonly upregulated in both 786-O and A498 ccRCC cells resistant to sunitinib (SUNR) and axitinib (AXIR).

Supplementary Table 1: Common proteins upregulated in SUNR and AXIR conditionned media

1	LCN2
2	CTHRC1
3	MMP1
4	PSG1;PSG3;PSG9
5	NID2
6	PSG1;PSG3;PSG5;PSG9
7	IL6
8	C3
9	L1CAM
10	CFB
11	ERVMER34-1
12	COL12A1
13	CXCL5
14	CXCL8
15	ANGEL2
16	FBN1
17	PSG9
18	PSG1;PSG3
19	PSG1
20	TENM3;TENM4
21	PLAT
22	SOD2
23	MATN2
24	CDH4
25	RNASEH2A
26	CTSS
27	SIRPB1
28	HS6ST1;HS6ST2
29	RELN
30	PLOD2
31	CXCL1
32	ASS1
33	MCAM
34	INHBA
35	EFNA1
36	CCN2
37	ACP5
38	ANPEP
39	LRRN4
40	OR5AC2
41	CD44
42	C1QTNF1
43	DNASE2
44	FSTL1
45	TENM3
46	ICOSLG
47	PSG3;PSG5;PSG9
48	ATRN
49	PCSK9
50	CXCL1;CXCL2
51	TNC
52	RGMB

53	SORT1
54	PROS1
55	PTX3
56	OAF
57	PSG1;PSG5
58	CAT
59	FAT1
60	PLSCR3
61	AGA
62	COL6A2
63	GCNT3
64	IGFBP7
65	SERPINB9
66	B3GNT7
67	ANKRD30A
68	SERPINI1
69	GFRA1
70	ICAM2
71	ANTXR1
72	DKK3
73	TAGLN
74	CHRDL1
75	NRXN3
76	ACP6
77	CADM1
78	NEGR1
79	PTPRM
80	CPA4
81	NXPE3
82	GJC1
83	ICAM1
84	HS6ST2
85	BPHL
86	TNFRSF9
87	TP53I3
88	LCP1
89	GSTM3
90	TLL2
91	MDGA2
92	C1QTNF6
93	FJX1
94	GLA
95	PTPRS
96	HMOX1
97	ARSB
98	FTH1
99	BCAM
100	CD81
101	LSR
102	EFEMP2
103	FZD2;FZD7
104	EFNB1

105	CLMP
106	EXOC3L4
107	CCDC80
108	GALNT18
109	LOX
110	LAMB2
111	HEXB
112	DCBLD1
113	CLU
114	LY96
115	CTSH
116	IDUA
117	PTPRB
118	CNRIP1
119	ADAM9
120	EXT1
121	IGSF3
122	TMBIM1
123	GBA
124	LUM
125	PPIF
126	NECTIN2
127	IGF2R
128	EXT2
129	TMEM132A
130	IFI30
131	GLUD1
132	TNFRSF11B
133	ACP2
134	PPIE;PPIF
135	SPRYD4
136	TOR1B
137	CUX2
138	IGSF8
139	CRAT
140	KYNU
141	PTTG1IP
142	SDCBP
143	ECH1
144	ADAMTSL3
145	KIAA1755
146	ASAH1
147	VEGFC
148	ACO2
149	FSTL3
150	DSC2
151	PCK2
152	GOT2
153	IL6ST
154	ITGB3
155	FXN
156	BCAT2

157	SEMA4C
158	DPP7
159	GCSH
160	QSOX1
161	PSG5
162	TIMP1
163	B2M
164	C1QBP
165	BNC2
166	MAN2B1
167	PLSCR1
168	CDCP1
169	PEPD
170	DPP4
171	C1R;C1RL
172	NECTIN1
173	ST3GAL1
174	CEMIP2
175	HIBADH
176	C1GALT1C1
177	HIBCH
178	PTS
179	CACHD1
180	SRPX
181	FCAMR
182	CADM4
183	TLL1;TLL2
184	SERPINB8;SERPINB9
185	TGFB2
186	TXNRD2
187	EPHB2
188	C11orf54
189	ROBO1
190	TSPAN4
191	CCN3
192	CHID1
193	OGFOD3
194	FDX1
195	TSPAN14
196	ECI1
197	CNPY3
198	AGT
199	IL4I1
200	SERPINA5
201	ST3GAL1;ST3GAL2
202	CYCS
203	ATF6
204	DLST
205	SORCS2
206	CST3
207	ACOT1
208	CTSO

209	SLIT2
210	DLD
211	ADGRE5
212	IST1
213	MMP2;MMP7
214	MMP2
215	ADAM12
216	CCL2
217	POLDIP2
218	MRC2
219	ACAT1
220	MEGF8
221	GRPEL1
222	LHPP
223	HLA-A;HLA-B
224	ABHD10
225	PAPPA
226	PLD3
227	ACAA2
228	SUMF2
229	SERPINB7
230	SERPINB8
231	SDK1
232	MSRA
233	POGLUT3
234	LONP1
235	EFNA4
236	ZNF428
237	GALNT12
238	ANXA5
239	LAMB3
240	CHMP1B
241	ATP1B3
242	B4GALT3
243	RMDN3
244	SLC31A1
245	MMP9
246	FKBP2
247	CDH13
248	SERPING1
249	MVB12A
250	ANXA11
251	STX7
252	NFU1
253	LRPPRC
254	CLDN1
255	MGAT5
256	MIX23
257	ADAM10
258	ANXA6
259	CPNE2
260	ATP5F1B

261	NIPSNAP3A
262	TXN2
263	HSPD1
264	SH3BGRL
265	DIABLO
266	ADGRG1
267	EML2
268	HSPA9
269	PRDX3
270	PLOD1
271	TOLLIP

Supplementary Table 2: Common genes upregulated in 786-O SUNR and AXIR

1	'ENG'
2	'SULF2'
3	'KCNIP1'
4	'LIMCH1'
5	'H1F0'
6	'PRKCZ'
7	'AJAP1'
8	'CHRDL1'
9	'SPX'
10	'TCF7L1'
11	'PRXL2A'
12	'CDH4'
13	'NPPB'
14	'SULT1A4'
15	'EPN2'
16	'LBH'
17	'RELN'
18	'CLDN16'
19	'TNFSF12'
20	'COL4A4'
21	'AUTS2'
22	'SCN8A'
23	'RRAGD'
24	'APBB1'
25	'TP53I11'
26	'SUN3'
27	'INPP4B'
28	'OAS2'
29	'IGFBP2'
30	'ALDH1L2'
31	'RAB19'
32	'TMEM40'
33	'ERICH5'
34	'C4BPB'
35	'KRT17'
36	'TMEM98'
37	'PTPRS'
38	'AQP1'
39	'JPH2'
40	'SARDH'
41	'PHLDB3'
42	'SYT17'
43	'APBA1'
44	'PSG2'
45	'FBLN1'
46	'SMTNL2'
47	'RAB3IL1'
48	'TUBB4A'
49	'WNT5B'
50	'CRISPLD1'
51	'PDPN'
52	'EPHB1'
53	'LOC1006530
54	'HDX'

55 'PQLC2L'
56 'CELF2'
57 'MX2'
58 'TAGLN'
59 'GPRC5C'
60 'ZNF114'
61 'SAMD5'
62 'ERVMER34-
63 'C6orf132'
64 'ULK2'
65 'TLR2'
66 'ACSS1'
67 'ADAM28'
68 'ATP8B2'
69 'LRFN3'
70 'RENBP'
71 'SLAIN1'
72 'ANPEP'
73 'TMEM130'
74 'KIF6'
75 'TSPAN18'
76 'ZNF716'
77 'B4GALNT2'
78 'PCSK9'
79 'MLPH'
80 'MX1'
81 'SLC37A1'
82 'ANKRD6'
83 'FAM89A'
84 'PSG9'
85 'KCTD16'
86 'FAM19A2'
87 'ELFN1'
88 'GATA3'
89 'CXCL16'
90 'ARL10'
91 'GALNT12'
92 'SERPINF1'
93 'TLR4'
94 'CHST13'
95 'FAM43A'
96 'ETNK2'
97 'COL12A1'
98 'FCGRT'
99 'SCIN'
100 'PCDH20'
101 'SORL1'
102 'KCNK6'
103 'KLHL30'
104 'STK33'
105 'LPL'
106 'TMEM255B'
107 'NPR3'
108 'C11orf52'
109 'PSG5'
110 'KLF8'

111 'PTCHD1'
112 'HIST1H3E'
113 'PSG8'
114 'SORCS2'
115 'WTIP'
116 'PDE3B'
117 'ARMC4'
118 'PIK3IP1'
119 'NOXA1'
120 'HS3ST3A1'
121 'EFHD1'
122 'BPIFA2'
123 'ST6GALNAC
124 'OLR1'
125 'LRRC75A'
126 'FAM189A1'
127 'PLXNA2'
128 'SLC16A9'
129 'C1orf115'
130 'LOC1053748
131 'AMDHD1'
132 'PHF21B'
133 'CXCL12'
134 'L1CAM'
135 'MYH14'
136 'CDKN1C'
137 'IGFBP4'
138 'COL4A3'
139 'GSTT2'
140 'ZFP69'
141 'MEIS3'
142 'COLEC10'
143 'CSNK2A3'
144 'GAB3'
145 'IGFBP5'
146 'XK'
147 'HIST1H2AD'
148 'PSG11'
149 'PHOSPHO2-
150 'RIMS1'
151 'MKX'
152 'SSX1'
153 'LMO2'
154 'TECTA'
155 'HOXA7'
156 'SERPING1'
157 'NHLRC1'
158 'RASAL1'
159 'PCDHB11'
160 'KRT33B'
161 'LEF1'
162 'TFAP2C'
163 'RSAD2'
164 'LY6G5C'
165 'PSG1'
166 'NR0B1'

167 'SFMBT2'
168 'C11orf45'
169 'GRIN3B'
170 'TMEM59L'
171 'PSG3'
172 'TNNT2'
173 'SLC15A3'
174 'PRRX2'
175 'MYO5C'
176 'LY6K'
177 'GNG4'
178 'CRYAB'
179 'NPNT'
180 'ESYT3'
181 'B3GNT3'
182 'DACH1'
183 'FN3K'
184 'CYP2J2'
185 'CST6'
186 'DLK2'
187 'OAS1'
188 'SLC26A11'
189 'HS6ST2'
190 'IFI27'
191 'NME4'
192 'IL2RG'
193 'ATP8B1'
194 'NTN1'
195 'PLAT'
196 'CPA4'
197 'NOG'
198 'ALOX5'
199 'PSG4'
200 'LRFN1'
201 'MMP17'
202 'DNER'
203 'NFATC1'
204 'SLC9A2'
205 'EHD3'
206 'SETBP1'
207 'IL31RA'
208 'PLEKHB1'
209 'NRG2'
210 'MARCH4'
211 'CHRM4'
212 'GPM6A'
213 'ELOVL4'
214 'ADAMTS10'
215 'MAPK8IP1'
216 'GPR75-ASB[illegible]
217 'PDGFRL'
218 'ICAM2'
219 'CALHM6'
220 'PAX9'
221 'CXCL11'
222 'SSX5'

223 'ZNF362'
224 'NPR2'
225 'FSD1'
226 'GRK3'
227 'DGKA'
228 'ERP27'
229 'CYP1A1'
230 'TRAF5'
231 'OSBPL1A'
232 'ADAMTS14'
233 'KRT81'
234 'PDZD4'
235 'PEX11G'
236 'SCARA3'
237 'FRY'
238 'TSPAN15'
239 'MYO7A'
240 'RTN1'
241 'MATN2'
242 'LRRC63'
243 'MCF2L'
244 'PRTN3'
245 'HRK'
246 'TMEM236'
247 'EPSTI1'
248 'HCN4'
249 'DOCK11'
250 'ALPI'
251 'CSF2RA'
252 'PRR26'
253 'CCDC85A'
254 'IL27RA'
255 'GBX2'
256 'PSG7'
257 'PTP4A3'
258 'BIRC7'
259 'SCD5'
260 'ADRB2'
261 'GSTT2B'
262 'SIGLEC15'
263 'KCNK15'
264 'C5AR1'
265 'COLCA2'
266 'CYS1'
267 'HIST1H2AK'
268 'HID1'
269 'GALNT18'
270 'IGFL2'
271 'MDGA1'
272 'CLDN3'
273 'HMGA1'
274 'IL1RAPL1'
275 'CECR2'
276 'GSTM3'
277 'SLC4A8'
278 'SDK1'

279 'GJD3'
280 'TRPV2'
281 'LGI2'
282 'RAB11FIP4'
283 'DPEP1'
284 'MPP2'
285 'SPTBN5'
286 'CEBPA'
287 'PXDNL'
288 'RASGRP3'
289 'NUPR1'
290 'TMEM174'
291 'FAM189A2'
292 'STEAP1B'
293 'GPER1'
294 'C4orf19'
295 'STON1'
296 'LOC1103846
297 'TMEM238'
298 'NEIL1'
299 'MN1'
300 'CABYR'
301 'ST6GALNAC
302 'CXADR'
303 'PPP1R36'
304 'HSPB2'
305 'RSPO3'
306 'SYNGR1'
307 'RIPOR3'
308 'ANO9'
309 'BCAM'
310 'ALPP'
311 'RAET1G'
312 'TNFSF12-TN
313 'CXCL10'
314 'WHRN'
315 'ASS1'
316 'CASP1'
317 'DEGS2'
318 'TRIB2'
319 'PDZRN4'
320 'SEMA3G'
321 'SALL2'
322 'CALB1'
323 'LOC1019285
324 'CYP24A1'
325 'C15orf62'
326 'SSTR1'
327 'CERS4'
328 'SH2D2A'
329 'POU3F3'
330 'NAT8L'
331 'GRK5'
332 'FBXO44'
333 'EMID1'
334 'PCDHB8'

335	'CCDC181'
336	'SEMA4A'
337	'GSTO2'
338	'ZNF98'
339	'GMPR'
340	'PPFIBP2'
341	'LIMS2'
342	'PPARGC1A'
343	'ZNF559-ZNF
344	'CSTA'
345	'CHADL'
346	'GCNT4'
347	'HIST1H2AI'
348	'DYSF'
349	'PLEKHA7'
350	'IFI44'
351	'TMEM139'
352	'BATF2'
353	'CHSY3'
354	'TFAP2A'
355	'CNRIP1'
356	'FAM20C'
357	'GPRASP2'
358	'CPM'
359	'CYBB'
360	'TMEM229B'
361	'DACT1'
362	'DNAJC25-GI
363	'BCL11B'
364	'SLC22A1'
365	'VTCN1'
366	'CYBRD1'
367	'NCALD'
368	'RIPOR2'
369	'MCOLN3'
370	'ACP5'
371	'ZNF285'
372	'NOTUM'
373	'GLI2'
374	'CTGF'
375	'SLC48A1'
376	'MSLN'
377	'TNFSF13B'
378	'PADI2'
379	'CRYM'
380	'DAAM2'
381	'CCDC160'
382	'TNNC1'
383	'C1orf226'
384	'UBE2L6'
385	'DHRS2'
386	'GABRR2'
387	'SPDEF'
388	'MGAT4C'
389	'GOLGA7B'
390	'WNT4'

391 'RNF150'
392 'SLCO2A1'
393 'NRXN2'
394 'PCDH1'
395 'OGDHL'
396 'ADAMTS15'
397 'LAMA1'
398 'LIMS3'
399 'PLPP2'
400 'INSYN2B'
401 'CILP2'
402 'FAM124A'
403 'SORT1'
404 'PLA2G16'
405 'WRB-SH3BG
406 'SLCO1B3'
407 'HIST1H2AE'
408 'ST6GAL1'
409 'TRPC4'
410 'TUB'
411 'TNFRSF14'
412 'CYP39A1'
413 'NCAM2'
414 'LCP1'
415 'LOC1079841
416 'LOC1079842
417 'NFAM1'
418 'RNF125'
419 'TP53AIP1'
420 'AGBL2'
421 'TEAD2'
422 'LHX2'
423 'ETV7'
424 'DAB2IP'
425 'EMILIN2'
426 'SLC9A3R2'
427 'SHISA9'
428 'LIPC'
429 'ZNF730'
430 'APC2'
431 'TMC6'
432 'POF1B'
433 'AFAP1L1'
434 'EBF4'
435 'LZTS1'
436 'CYP1A2'
437 'ALPG'
438 'LYPD3'
439 'OR2T8'
440 'FIGNL2'
441 'P2RX5'
442 'DCHS2'
443 'SVOP'
444 'BMP6'
445 'SYT16'
446 'BRSK1'

447 'ABLIM2'
448 'NOS1AP'
449 'OLFML2A'
450 'STX11'
451 'ST6GALNAC
452 'CFAP300'
453 'PNMA2'
454 'NYX'
455 'DBNDD1'
456 'GOLGA6L19
457 'LOC1053691
458 'IGF2BP1'
459 'TMEM178A'
460 'BEAN1'
461 'CST2'
462 'AKR1C2'
463 'NIM1K'
464 'DOK6'
465 'INSR'
466 'TMEM63C'
467 'ZSWIM5'
468 'MOB3B'
469 'MPP7'
470 'CCL5'
471 'WLS'
472 'FBN1'
473 'HIF1A'
474 'MAFA'
475 'LOC390937'
476 'CAPN3'
477 'SEPT6'
478 'OASL'
479 'SAA1'
480 'IGDCC4'
481 'ADAP1'
482 'SLC43A1'
483 'PIP5KL1'
484 'WNT7B'
485 'PADI1'
486 'IFIT1'
487 'SLAMF7'
488 'DPYSL2'
489 'SP9'
490 'KRT23'
491 'LRRC32'
492 'MATK'
493 'PLEKHG7'
494 'MMP25'
495 'IFIH1'
496 'GSTA4'
497 'EFNA1'
498 'EPB41L4A'
499 'CCL2'
500 'PTK7'
501 'BTG4'
502 'DBNDD2'

503 'NAP1L5'
504 'LIPH'
505 'SAMD12'
506 'TRIM7'
507 'CEACAM1'
508 'CD177'
509 'SNAPC1'
510 'LOC1079874
511 'COL15A1'
512 'ZNF385B'
513 'GPC2'
514 'IGF2'
515 'SPATA21'
516 'TRIM73'
517 'KRT86'
518 'SEPT4'
519 'SPRY4'
520 'GLT8D2'
521 'MAP2'
522 'VSIR'
523 'LOC388282'
524 'FCMR'
525 'TTC28'
526 'GUCA1B'
527 'SOHLH2'
528 'SULT2B1'
529 'RGS5'
530 'SARM1'
531 'RASGRF1'
532 'KRT7'
533 'LINC00452'
534 'ABI3'
535 'PIK3AP1'
536 'LOC1019280
537 'DISC1'
538 'YPEL1'
539 'SLC4A11'
540 'PCDHGA11'
541 'SYCP2'
542 'IQGAP2'
543 'NLRP3'
544 'OLIG3'
545 'FGF1'
546 'ICAM4'
547 'C5orf46'
548 'SLC22A17'
549 'TENT5C'
550 'NPAS3'
551 'SLC7A2'
552 'SLC19A3'
553 'HIST1H3H'
554 'KCNQ4'
555 'KCNB2'
556 'LY6E'
557 'PLA2G4C'
558 'SHBG'

559 'SLC38A5'
560 'PJVK'
561 'C13orf46'
562 'ROBO4'
563 'CAVIN2'
564 'CYP2U1'
565 'SPRY1'
566 'TRIML2'
567 'REEP2'
568 'LRRC56'
569 'KIF17'
570 'PCDHGB3'
571 'NEGR1'
572 'KIAA1211'
573 'SLC20A2'
574 'PROS1'
575 'KCTD12'
576 'CYSTM1'
577 'GCNA'
578 'CLGN'
579 'ARHGAP9'
580 'GPNMB'
581 'ADAMTS7'
582 'OPLAH'
583 'SAMD9L'
584 'PPP1R32'
585 'SYBU'
586 'TMEM52B'
587 'MOCS1'
588 'IFI6'
589 'JDP2'
590 'GOLGA8M'
591 'CSPG5'
592 'DUSP19'
593 'TEKT4'
594 'CYP17A1'
595 'EMP1'
596 'FES'
597 'ARC'
598 'LOC283710'
599 'RTL5'
600 'KCNF1'
601 'MYO7B'
602 'CSGALNACT
603 'PTH1R'
604 'RAB17'
605 'LOC644634'
606 'TCHH'
607 'TNXB'
608 'FLJ45513'
609 'VWF'
610 'ANGPTL6'
611 'PARD6G'
612 'MARCO'
613 'RASL10B'
614 'ETHE1'

615 'TMEM37'
616 'NID2'
617 'HOXA13'
618 'DISP2'
619 'SHANK3'
620 'LOC1079859
621 'DEPTOR'
622 'ISG20'
623 'FTH1'
624 'SERPINB9'
625 'CORO2A'
626 'TAS1R3'
627 'CCDC69'
628 'PLSCR4'
629 'PXYLP1'
630 'ARHGAP23'
631 'STOX1'
632 'ANTXR1'
633 'COLGALT2'
634 'TMEM255A'
635 'SPTB'
636 'TMEM38A'
637 'TUBAL3'
638 'GDAP1'
639 'NLGN1'
640 'MMP1'
641 'KCNMB1'
642 'TIMP4'
643 'AKR1C1'
644 'LSR'
645 'MAP1LC3C'
646 'B3GNT7'
647 'BAHCC1'
648 'TM4SF19'
649 'HMOX1'
650 'CRIM1'
651 'TMEM221'
652 'SPEG'
653 'RASL10A'
654 'CERS1'
655 'CHRNE'
656 'RAB39B'
657 'OSCAR'
658 'BMPER'
659 'DUSP2'
660 'NMNAT2'
661 'MLC1'
662 'SPEF1'
663 'GDF1'
664 'PCSK1N'
665 'GNRH2'
666 'NUDT7'
667 'MILR1'
668 'ARHGAP40'
669 'ZFP57'
670 'C4orf47'

671 'PLGLB2'
672 'IL17RB'
673 'PRKCG'
674 'VTN'
675 'SLC25A27'
676 'IFITM10'
677 'OSR1'
678 'CBLN3'
679 'ANKRD1'
680 'AMOTL1'
681 'TIAM2'
682 'GRIN2A'
683 'PALM2-AKAI
684 'GSG1'
685 'F2RL1'
686 'TAGLN3'
687 'OPHN1'
688 'UAP1L1'
689 'NXPH4'
690 'FAM47E-STE
691 'NOV'
692 'ZNF219'
693 'ARPP21'
694 'CADM4'
695 'GFI1'
696 'SYNDIG1L'
697 'CD14'
698 'CORO2B'
699 'SERPINI1'
700 'CFB'
701 'PATL2'
702 'BATF3'
703 'CSRNP3'
704 'CDK5R2'
705 'FYN'
706 'TP53I3'
707 'GOLGA8Q'
708 'KPNA5'
709 'PARD3B'
710 'PRSS27'
711 'PRDM6'
712 'TMEM191C'
713 'RHOBTB1'
714 'LOC1010601
715 'RAPGEF3'
716 'TLL2'
717 'DLL3'
718 'LOC1079846
719 'UPK1A'
720 'DCST2'
721 'SESN3'
722 'VASH1'
723 'FMO5'
724 'OSBP2'
725 'CRB2'
726 'GRIK5'

727 'SH3RF3'
728 'FOXD4L4'
729 'C9orf152'
730 'MYT1'
731 'PLCB2'
732 'AMIGO1'
733 'DLGAP3'
734 'CPLX3'
735 'ZNF704'
736 'BCAN'
737 'RNF112'
738 'CALB2'
739 'EPPK1'
740 'PHYHIPL'
741 'NEURL1'
742 'CPA5'
743 'CLIP3'
744 'FLVCR2'
745 'MCAM'
746 'C12orf60'
747 'ZBTB44'
748 'TMEFF2'
749 'CDRT1'
750 'RBM44'
751 'AMPH'
752 'HELZ2'
753 'NPR1'
754 'TXK'
755 'FGFR2'
756 'NEK10'
757 'TPPP3'
758 'SC5D'
759 'PLCG2'
760 'PDZD2'
761 'CHAC1'
762 'CFAP57'
763 'GPR15'
764 'VWA2'
765 'ACHE'
766 'RCAN2'
767 'CEACAM19'
768 'DOK7'
769 'KIRREL3'
770 'CARD9'
771 'LOX'
772 'LOC1079858
773 'CTHRC1'
774 'IGFBP7'
775 'BEST3'
776 'TTLL3'
777 'ARMH1'
778 'WNT5A'
779 'FRMD4A'
780 'ITGA1'
781 'C11orf1'
782 'ZFHX2'

783 'PNPLA7'
784 'DDX58'
785 'IQCN'
786 'RAB6B'
787 'BTBD6'
788 'MAL2'
789 'TBC1D3K'
790 'FLRT3'
791 'LOC653513'
792 'KIAA0319'
793 'ANKDD1A'
794 'FGF5'
795 'JPH1'
796 'ITGB4'
797 'SLC3A1'
798 'HSPA2'
799 'TMEM140'
800 'LOC1122682
801 'PEAR1'
802 'WWC2'
803 'WASF3'
804 'CSAG2'
805 'LOC1053732
806 'LOC1079861
807 'SLC27A2'
808 'RAPGEF4'
809 'ADCY5'
810 'TMEM132B'
811 'ZG16B'
812 'NTN5'
813 'RINL'
814 'RTN4RL1'
815 'RHBDL3'
816 'EDA'
817 'HEY2'
818 'GRID1'
819 'GRIN2D'
820 'AQP4'
821 'RGPD1'
822 'MAP6'
823 'MST1R'
824 'NGFR'
825 'POU5F1B'
826 'OLFML3'
827 'IL22RA1'
828 'CELF6'
829 'C4B'
830 'ACSS3'
831 'ADAMTS12'
832 'PLVAP'
833 'FAM83A'
834 'RGS11'
835 'MARVELD3'
836 'PLCH2'
837 'PHACTR1'
838 'DUXA'

839 'IFI35'
840 'MYBPH'
841 'BRSK2'
842 'AHNAK2'
843 'GPBAR1'
844 'DAGLB'
845 'SYT1'
846 'GUCY1A2'
847 'CCDC84'
848 'HERC5'
849 'CCDC80'
850 'ATP2A1'
851 'ISG15'
852 'STC2'
853 'KLHL24'
854 'CHN1'
855 'CTIF'
856 'LRP4'
857 'PCDHB13'
858 'ULBP1'
859 'DGKD'
860 'TLE2'
861 'VASH2'
862 'ROBO3'
863 'ANK2'
864 'ITIH5'
865 'DNASE2'
866 'CYP27A1'
867 'SOCS1'
868 'OBSCN'
869 'AFAP1'
870 'ZFAND4'
871 'TMEM45A'
872 'CITED4'
873 'NKPD1'
874 'HGFAC'
875 'IL11RA'
876 'PAIP2B'
877 'SELPLG'
878 'CNTNAP3'
879 'CAMK2D'
880 'CD22'
881 'GREB1'
882 'NQO1'
883 'SLC46A3'
884 'VPS35L'
885 'RRAGB'
886 'GPR146'
887 'PODXL'
888 'FSTL1'
889 'LAMA2'
890 'SLC25A4'
891 'C20orf96'
892 'MAGEH1'
893 'CLIP2'
894 'NPAS1'

895 'SGK3'
896 'REPS2'
897 'PLEKHH1'
898 'MAFK'
899 'SP140'
900 'THBS1'
901 'GOLGA8R'
902 'CBSL'
903 'FOXJ1'
904 'TRMT9B'
905 'CORO6'
906 'FDXACB1'
907 'PLAC8'
908 'THYN1'
909 'TMEM136'
910 'MERTK'
911 'ALDH3A2'
912 'GAD1'
913 'PLCXD2'
914 'SEMA3E'
915 'CASP4'
916 'PCDH10'
917 'RBM20'
918 'MSANTD1'
919 'VPS26B'
920 'TMEM47'
921 'GRK4'
922 'CLU'
923 'WDR97'
924 'AGA'
925 'MISP3'
926 'THBD'
927 'ABHD4'
928 'HTR1D'
929 'PRKAA2'
930 'PAPPA'
931 'GDPD1'
932 'RTN4R'
933 'FAM110B'
934 'CPT1B'
935 'PEPD'
936 'SP110'
937 'ADGRB3'
938 'PSORS1C1'
939 'MIOX'
940 'RHOB'
941 'PTPRU'
942 'NPY4R2'
943 '112267922'
944 '112268338'
945 'IP6K3'
946 'SGSM1'
947 'MUCL3'
948 'PIANP'
949 'ALDH3A1'
950 'NAALADL2'

951 'RGS22'
952 'FILIP1'
953 'FAM19A3'
954 'SH2D6'
955 'TPRG1'
956 'PURG'
957 'POLN'
958 'SHROOM2'
959 'SNORC'
960 'NOTCH3'
961 'SH3TC1'
962 'PPP2R2B'
963 'RCN3'
964 'SLC4A5'
965 'BCHE'
966 'FOXD4L5'
967 'ST8SIA4'
968 'MMP28'
969 'GAL3ST4'
970 'DENND1C'
971 'PRRT1'
972 'FAM167B'
973 'PDE5A'
974 'CACNA1H'
975 'RSPH1'
976 'BOC'
977 'PTPRJ'
978 'IDNK'
979 'EPHA6'
980 'ARHGEF12'
981 'SERTAD4'
982 'GOLGA8J'
983 'RILP'
984 'ACAD8'
985 'PIH1D2'
986 'MSRA'
987 'IGSF23'
988 'FADS3'
989 'WFS1'
990 'PSCA'
991 'THAP8'
992 'GFRA1'
993 'CGN'
994 'SLIT2'
995 'FTCDNL1'
996 'LOC1027241
997 'SAMD10'
998 'CREG2'
999 'LOC401478'
1000 'DLX2'
1001 'PABPC4L'
1002 'TJP3'
1003 'MMP23B'
1004 'SP140L'
1005 'IFI30'
1006 'GOLGA6L4'

1007 'NLGN3'
1008 'KLHL29'
1009 'ARFGEF3'
1010 'RNF122'
1011 'PRTG'
1012 'IL7R'
1013 'PANX1'
1014 'TRABD2A'
1015 'HIST1H1C'
1016 'PDE11A'
1017 'GPR137B'
1018 'NEU1'
1019 'HIST1H2AC'
1020 'SLC16A7'
1021 'TIMM8B'
1022 'PAMR1'
1023 'PLCD4'
1024 'ISYNA1'
1025 'EHD1'
1026 'EXOC6'
1027 'TRPM4'
1028 'FAM71E1'
1029 'GPR35'
1030 'MGAT4A'
1031 'MAP3K15'
1032 'TMCC2'
1033 'VAT1'
1034 'HLA-F'
1035 'ASRGL1'
1036 'NINL'
1037 'CDC42EP2'
1038 'SNPH'
1039 'NEBL'
1040 'ZNF846'
1041 'PRKN'
1042 'ATRX'
1043 'D2HGDH'
1044 'PARD6A'
1045 'TTC14'
1046 'STON2'
1047 'GLI3'
1048 'DKK3'
1049 'ANKRD18B'
1050 'C19orf38'
1051 'IDUA'
1052 'FBXL18'
1053 'NFIB'
1054 'LNPEP'
1055 'MACROD2'
1056 'FAM71F2'
1057 'MYO5B'
1058 'GDPD5'
1059 'FOXRED2'
1060 'PHYH'
1061 'ZSCAN16'
1062 'MST1L'

1063 'MUC20'
1064 'C11orf54'
1065 'TTC6'
1066 'MSR1'
1067 'ANO7'
1068 'PREX1'
1069 'BCO2'
1070 'AMBP'
1071 'MROH8'
1072 'LOC1122683
1073 'HOXC11'
1074 'RETREG1'
1075 'TMEM56-RW
1076 'GOLGA6L3'
1077 'RAB32'
1078 'NCOA7'
1079 'ACOT1'
1080 'OGT'
1081 'PPARGC1B'
1082 'NLRX1'
1083 'GRN'
1084 'ZNF391'
1085 'SIK2'
1086 'FJX1'
1087 'CDS2'
1088 'ADSSL1'
1089 'PCDHB16'
1090 'PAQR6'
1091 'DZIP1'
1092 'TBC1D12'
1093 'CMTM3'
1094 'MARCH2'
1095 'GPR176'
1096 'LTBP2'
1097 'CCDC110'
1098 'SOWAHD'
1099 'HPS5'
1100 'UFSP2'
1101 'TPP1'
1102 'TMEM240'
1103 'NANOS1'
1104 'C19orf66'
1105 'WASHC2A'
1106 'OSTM1'
1107 'CWF19L2'
1108 'FKBP9'
1109 'TRIM21'
1110 'PRSS35'
1111 'C2orf27A'
1112 'ACP2'
1113 'TK2'
1114 'SLC17A5'
1115 'MDM4'
1116 'IQUB'
1117 'KRT34'
1118 'PYGO1'

1119	'PTPRM'
1120	'EPHA4'
1121	'NHLRC2'
1122	'LAD1'
1123	'KCP'
1124	'ST3GAL6'
1125	'PCDHB2'
1126	'FAM102B'
1127	'ABCB6'
1128	'IGFN1'
1129	'CNTNAP1'
1130	'LYPD1'
1131	'FBXO36'
1132	'RASSF2'
1133	'LGALSL'
1134	'NPC2'
1135	'CEP295NL'
1136	'WDR63'
1137	'MAD1L1'
1138	'EFEMP2'
1139	'SRPX'
1140	'IGBP1'
1141	'HS1BP3'
1142	'RAB30'
1143	'EBI3'
1144	'CCL28'
1145	'BOK'
1146	'ARHGAP24'
1147	'ZNF460'
1148	'ZDHHC1'
1149	'CHURC1-FN
1150	'NR3C2'
1151	'HINFP'
1152	'NKAPD1'
1153	'VLDLR'
1154	'ARHGEF28'
1155	'PDZD11'
1156	'LCN12'
1157	'FAM166A'
1158	'CCPG1'
1159	'LOC1009967
1160	'MAPT'
1161	'MMP15'
1162	'SNX18'
1163	'CNTNAP3B'
1164	'FAM171B'
1165	'IER5L'
1166	'ZDHHC11'
1167	'GPR173'
1168	'CA11'
1169	'LGR4'
1170	'IFIT2'
1171	'C16orf74'
1172	'RND3'
1173	'MTHFS'
1174	'ACCS'

1175 'CPA6'
1176 'EPB41L1'
1177 'GTDC1'
1178 'RHPN1'
1179 'TUBB2A'
1180 'BDNF'
1181 'SUSD1'
1182 'NXN'
1183 'REC8'
1184 'KLF5'
1185 'ZFYVE26'
1186 'FOXA1'
1187 'TIRAP'
1188 'FAT1'
1189 'CHORDC1'
1190 'ITGA6'
1191 'ALDH9A1'
1192 'NAT2'
1193 'CDH3'
1194 'SH2D3C'
1195 'LOC1019275
1196 'OLIG2'
1197 'FST'
1198 'LOC1053723
1199 'LOC1053778
1200 'CD226'
1201 'LDB3'
1202 'KLHDC7B'
1203 'PKD1L2'
1204 'NOSTRIN'
1205 'RASGRP4'
1206 'KRBA2'
1207 'DIRAS1'
1208 'EDNRB'
1209 'ZNF385C'
1210 'FAAH'
1211 'TIGD3'
1212 'ADGRG3'
1213 'FOXS1'
1214 'ABCA4'
1215 'FNDC5'
1216 'PARM1'
1217 'PCDHB5'
1218 'GGT5'
1219 'PDE7B'
1220 'PALD1'
1221 'CPAMD8'
1222 'AMY2B'
1223 'RCOR2'
1224 'SYPL2'
1225 'DCAF4L1'
1226 'C4orf54'
1227 'HPCA'
1228 'IRF8'
1229 'IL1B'
1230 'KCNJ11'

1231 'LOC400499'
1232 'FAM177B'
1233 'LSP1'
1234 'ABCB4'
1235 'PLAG1'
1236 'TLR9'
1237 'SOBP'
1238 'NIPSNAP3B'
1239 'ASIC4'
1240 'SEPT3'
1241 'SUSD2'
1242 'TMPRSS15'
1243 'KLK10'
1244 'C14orf132'
1245 'PLEKHG1'
1246 'PYGM'
1247 'MPP4'
1248 'SELENOP'
1249 'SBK2'
1250 'SLC1A3'
1251 'NCF1'
1252 'SLC22A2'
1253 'TEK'
1254 'TH'
1255 'UGT2B17'
1256 'XDH'
1257 'NDNF'
1258 'TET1'
1259 'COLQ'
1260 'MRO'
1261 'CILP'
1262 'RDH16'
1263 'FCGBP'
1264 'HNRNPLL'
1265 'SDR42E1'
1266 'LRRC4B'
1267 'GABBR2'
1268 'GPRASP1'
1269 'NAV3'
1270 'SNTA1'
1271 'C19orf33'
1272 'TRIM25'
1273 'PPP2R1B'
1274 'DUSP3'
1275 'C3orf58'
1276 'APOBEC3G'
1277 'RALGAPA2'
1278 'TMEM217'
1279 'CTSD'
1280 'TUBB2B'
1281 'TCEAL4'
1282 'KLHDC8B'
1283 'PPP1R3C'
1284 'SOX13'
1285 'WASHC2C'
1286 'ACOT11'

1287 'HIST1H2BK'
1288 'PQBP1'
1289 'PDE6D'
1290 'FMNL2'
1291 'GOLGA8N'
1292 'TRIM14'
1293 'CACNA2D3'
1294 'SPRED3'
1295 'NKIRAS2'
1296 'NRXN3'
1297 'TMCC3'
1298 'NOCT'
1299 'ANO2'
1300 'HSD11B2'
1301 'RIN3'
1302 'RFTN1'
1303 'CCDC82'
1304 'BMPR2'
1305 'RLIM'
1306 'TAP1'
1307 'RDX'
1308 'INCA1'
1309 'SLFN12'
1310 'EGFL8'
1311 'ASAH1'
1312 'LOC1122682
1313 'RASA4B'
1314 'UPK3B'
1315 'MTRNR2L3'
1316 'CDH17'
1317 'ATP6V0A1'
1318 'DYNC2H1'
1319 'NEK11'
1320 'TMEM268'
1321 'PUS3'
1322 'PRICKLE4'
1323 'NPIPB9'
1324 'TRMT10B'
1325 'SNX19'
1326 'ATAD3C'
1327 'SDHD'
1328 'FAM118B'
1329 'CC2D2A'
1330 'C2orf92'
1331 'IRF9'
1332 'CTTN'
1333 'ZW10'
1334 'KPTN'
1335 'NEXN'
1336 'EIF2AK2'
1337 'GCNT3'
1338 'ERCC2'
1339 'ADAMTS13'
1340 'CHRM3'
1341 'P2RY8'
1342 'GTF2H1'

1343 'ADAM11'
1344 'RP1'
1345 'BFSP1'
1346 'TENM3'
1347 'CCR10'
1348 'C1orf198'
1349 'HIST1H2BD'
1350 'YAP1'
1351 'KIZ'
1352 'SCPEP1'
1353 'DOCK2'
1354 'ZNF181'
1355 'VPS13B'
1356 'FOXC1'
1357 'ING2'
1358 'CITED1'
1359 'MYEF2'
1360 'TTC39B'
1361 'CYP4V2'
1362 'AP5S1'
1363 'MFSD12'
1364 'SLC16A13'
1365 'GET4'
1366 'KIF16B'
1367 'LOC1079848[illegible]
1368 'LDHD'
1369 'NYAP1'
1370 'DDR2'
1371 'ARHGAP1'
1372 'ANKRD2'
1373 'HYLS1'
1374 'ZPR1'
1375 'ZNF792'
1376 'YIPF6'
1377 'SNX12'
1378 'ARHGEF37'
1379 'PIWIL4'
1380 'GABRB3'
1381 'LRRC8B'
1382 'NFE2L1'
1383 'ST3GAL1'
1384 'C2orf15'
1385 'SLC9A7'
1386 'IFT46'
1387 'RGS9BP'
1388 'PLCD3'
1389 'SDCBP2'
1390 'ERBB3'
1391 'TBCEL'
1392 'ABCA5'
1393 'RNF227'
1394 'NPIPB6'
1395 'USP11'
1396 'TGFBR3'
1397 'METTL8'
1398 'SNX24'

1399 'APOOL'
1400 'FBXO25'
1401 'NUTM2B'
1402 'LRP5L'
1403 'APOBEC3F'
1404 'LMAN2L'
1405 'MAPK11'
1406 'TRIM69'
1407 'SLC25A34'
1408 'NEK5'
1409 'ZNF850'
1410 'TMOD1'
1411 'FAM110A'
1412 'IFNGR1'
1413 'IFT122'
1414 'FAM241B'
1415 'MBP'
1416 'AGAP2'
1417 'LOC1053769
1418 'TGFBRAP1'
1419 'WDR81'
1420 'ARRB1'
1421 'TRPV1'
1422 'MSANTD4'
1423 'CERCAM'
1424 'HDAC9'
1425 'CBL'
1426 'CIB2'
1427 'OSGIN1'
1428 'RAG1'
1429 'TCN2'
1430 'CDCA7L'
1431 'SUN1'
1432 'COL11A2'
1433 'LHPP'
1434 'BPHL'
1435 'REXO2'
1436 'LOC1019294
1437 'APOE'
1438 'DCHS1'
1439 'SLC25A21'
1440 'HACD1'
1441 'RIOK3'
1442 'STOX2'
1443 'SLC35F2'
1444 'UBOX5'
1445 'NR4A2'
1446 'TCP11L1'
1447 'PPP1R15A'
1448 'FOXO4'
1449 'PIDD1'
1450 'NPAT'
1451 'CCNB3'
1452 'CBWD2'
1453 'BAMBI'
1454 'PYGB'

1455 'DNAAF5'
1456 'ADAMTSL4'
1457 'SP100'
1458 'ZFP69B'
1459 'STT3A'
1460 'ADAMTS5'
1461 'SIAE'
1462 'STS'
1463 'TGFB1I1'
1464 'CAPN5'
1465 'CUL5'
1466 'RNF214'
1467 'CD34'
1468 'EPG5'
1469 'ABCB7'
1470 'CCDC146'
1471 'ST14'
1472 'TPK1'
1473 'TRPM2'
1474 'ZNF202'
1475 'HS3ST1'
1476 'DDX6'
1477 'SH2D5'
1478 'CAMKMT'
1479 'PCED1A'
1480 'PGPEP1'
1481 'IL4I1'
1482 'DTX3L'
1483 'PSAT1'
1484 'TPRG1L'
1485 'RPUSD4'
1486 'UBE4A'
1487 'VPS11'
1488 'CBS'
1489 'CDSN'
1490 'FAM131A'
1491 'CHD5'
1492 'TFAP2E'
1493 'P2RX7'
1494 'CHDH'
1495 'TIE1'
1496 'CACNB4'
1497 'ZNF518B'
1498 'TRAM2'
1499 'HOMER1'
1500 'CCSER2'
1501 'MRGPRX3'
1502 'VPS41'
1503 'ZDHHC21'
1504 'SSPN'
1505 'ADAMTS3'
1506 'ZNF780A'
1507 'NECTIN2'
1508 'CPNE5'
1509 'KHDRBS3'
1510 'DDX10'

1511 'CCDC93'
1512 'RNF185'
1513 'HSH2D'
1514 'PRKRA'
1515 'SIM1'
1516 'ESF1'
1517 'ADORA2B'
1518 'LIN7C'
1519 'PLA2R1'
1520 'ASIC1'
1521 'PDE4D'
1522 'MINDY1'
1523 'FOXH1'
1524 'KDELC2'
1525 'PARP9'
1526 'TAF1D'
1527 'BNIP3L'
1528 'TRMT6'
1529 'ALG9'
1530 'CCDC180'
1531 'CPQ'
1532 'COL14A1'
1533 'MRPS9'
1534 'LOC107987[illegible]
1535 'SRCIN1'
1536 'SKIDA1'
1537 'CHRNA10'
1538 'MGST3'
1539 'NUAK1'
1540 'TPPP'
1541 'ZNF554'
1542 'TMEM86A'
1543 'SULT1C4'
1544 'GATD3A'
1545 'RIMS3'
1546 'QKI'
1547 'COMMD9'
1548 'PHLDB1'
1549 'EXT1'
1550 'SYDE1'
1551 'TAOK1'
1552 'ZNF235'
1553 'SIX1'
1554 'USP28'
1555 'CEP126'
1556 'SKIL'
1557 'PLEKHO2'
1558 'SLC16A10'
1559 'FAM53A'
1560 'NLN'
1561 'AP5Z1'
1562 'PID1'
1563 'NPIPB13'
1564 'LONRF3'
1565 'TINAGL1'
1566 'SLC35F6'

1567 'ADM2'
1568 'ANKRD36B'
1569 'PRKCE'
1570 'L3MBTL1'
1571 'LRRC20'
1572 'GJA3'
1573 'OPTN'
1574 'RPS6KA2'
1575 'CARS'
1576 'NR4A1'
1577 'CEP164'
1578 'SH3D19'
1579 'LACTB2'
1580 'ZNF160'
1581 'NAGK'
1582 'TUBA1A'
1583 'PPM1N'
1584 'NAV2'
1585 'RHBDF1'
1586 'RASA4'
1587 'MAP4K2'
1588 'SLC22A18'
1589 'TUFT1'
1590 'C2CD2L'
1591 'FSCN2'
1592 'SH3BP4'
1593 'GMDS'
1594 'XXYLT1'
1595 'SYT7'
1596 'ACVRL1'
1597 'CRTC1'
1598 'PCSK7'
1599 'ZSCAN26'
1600 'F2R'
1601 'RALGAPA1'
1602 'ZNF8'
1603 'JAM2'
1604 'GINM1'
1605 'ADAMTS2'
1606 'CTR9'
1607 'THBS3'
1608 'TOLLIP'
1609 'ZNF18'
1610 'SIRPB1'
1611 'GBA'
1612 'MCOLN2'
1613 'TMEM123'
1614 'ZSCAN12'
1615 'LYSMD2'
1616 'PLXND1'
1617 'TLCD2'
1618 'HOXD10'
1619 'DNAJC13'
1620 'CENPB'
1621 'LIMD2'
1622 'CAP2'

1623 'ABHD15'
1624 'AXL'
1625 'ME1'
1626 'TMEM159'
1627 'PGBD1'
1628 'FNDC3B'
1629 'TROVE2'
1630 'STAMBPL1'
1631 'PITPNM3'
1632 'IL17RE'
1633 'LRFN5'
1634 'PTGS2'
1635 'KBTBD11'
1636 'TMIE'
1637 'MSANTD2'
1638 'FAHD2B'
1639 'STX7'
1640 'DOCK5'
1641 'PRRT3'
1642 'LOC1079841
1643 'PJA1'
1644 'REEP3'
1645 'SH3GLB2'
1646 'BMP8B'
1647 'UEVLD'
1648 '112268375'
1649 'FAM210A'
1650 'CYTH3'
1651 'RETREG2'
1652 'ZBED3'
1653 'MARCKS'
1654 'RAPGEFL1'
1655 'NFRKB'
1656 'ERCC6L'
1657 '101929747'
1658 'VCPIP1'
1659 'NSFL1C'
1660 'SLC27A3'
1661 'PIK3R6'
1662 'SYTL2'
1663 'FRMPD3'
1664 'CEP295'
1665 'DIS3L'
1666 'PLA2G15'
1667 'ATM'
1668 'TBRG1'
1669 'TOR1B'
1670 'PIP4P1'
1671 'LTB4R2'
1672 'SRSF8'
1673 'GCNT2'
1674 'BRI3'
1675 'STX8'
1676 'BHMT2'
1677 'PLD1'
1678 'SLC16A2'

1679 'ARHGDIB'
1680 'IDH3B'
1681 'LOC1053714
1682 'ANKZF1'
1683 'ROPN1L'
1684 'RWDD4'
1685 'UHRF1BP1'
1686 'ASPHD2'
1687 'NFKBIE'
1688 'SEPT10'
1689 'PLEKHB2'
1690 'PEA15'
1691 'ILK'
1692 'FTL'
1693 'CCDC86'
1694 'CCDC88C'
1695 'CLN8'
1696 'FAM177A1'
1697 'ZNF503'
1698 'IRS1'
1699 'RPSAP58'
1700 'ITGA4'
1701 'DNAJB2'
1702 'GCA'
1703 'ADGRB2'
1704 'RHOU'
1705 'SMPDL3B'
1706 'TBC1D19'
1707 'INTS1'
1708 'ACVR2A'
1709 'PNRC1'
1710 'WBP1L'
1711 'PSMF1'
1712 'INTU'
1713 'XKR6'
1714 'BMP7'
1715 'WNT10A'
1716 'RUFY2'
1717 'UTRN'
1718 'HOXD3'
1719 'BBS5'
1720 'ZNF517'
1721 'ETS1'
1722 'ZFYVE9'
1723 'LNPK'
1724 'VPS9D1'
1725 'PELO'
1726 'SCNN1D'
1727 'HIBCH'
1728 'WNT2B'
1729 'PTS'
1730 'MAP3K2'
1731 'GNPTG'
1732 'DLAT'
1733 'GPR135'
1734 'NPIPB4'

1735 'GAB2'
1736 'PLLP'
1737 'NLRC5'
1738 'TAF1'
1739 'MTRNR2L1'
1740 'SGTB'
1741 'CARD8'
1742 'ZNF440'
1743 'SLC36A1'
1744 'GRAMD4'
1745 'ZNF518A'
1746 'SWAP70'
1747 'SBF2'
1748 'STK17B'
1749 'CNPPD1'
1750 'ACSL6'
1751 'CES4A'
1752 'GSTM2'
1753 'CCDC62'
1754 'ZNF408'
1755 'PIN4'
1756 'SEMA6C'
1757 'ZNF133'
1758 'DIABLO'
1759 'KLHL11'
1760 'STMN3'
1761 'INPP5J'
1762 'CLPTM1L'
1763 'TP53I13'
1764 'SRPRA'
1765 'FAM57A'
1766 'ZNF14'
1767 'NR2F2'
1768 'DDX18'
1769 'PPME1'
1770 'LOC1053740
1771 'PKIG'
1772 'CDC5L'
1773 'IRGQ'
1774 'LOC1053731
1775 'DYRK1B'
1776 'EFNB1'
1777 'ZBTB3'
1778 'DLC1'
1779 'NPIPB11'
1780 'NAGLU'
1781 'IKZF2'
1782 'SLC23A2'
1783 'G6PD'
1784 'RNF25'
1785 'PYROXD2'
1786 'DARS'
1787 'DGKE'
1788 'EPS8L2'
1789 'CCDC148'
1790 'DNAJC27'

1791 'MICA'
1792 'ARHGEF18'
1793 'ZNF230'
1794 'TRERF1'
1795 'PRX'
1796 'HRH1'
1797 'WIPF1'
1798 'FAM129A'
1799 'RMND5B'
1800 'INPP1'
1801 'TSSC4'
1802 'ERCC3'
1803 'SRXN1'
1804 'TMEM249'
1805 'NONO'
1806 'VXN'
1807 'DCPS'
1808 'PQLC3'
1809 'FHIT'
1810 'SMCR8'
1811 'HIPK3'
1812 'THTPA'
1813 'TEAD1'
1814 'GALK2'
1815 'ASB9'
1816 'ARHGEF10'
1817 'C7orf26'
1818 'UBQLNL'
1819 'HIST1H2BO'
1820 'ARHGAP32'
1821 'TM2D2'
1822 'SEL1L3'
1823 'TOM1'
1824 'MED12'
1825 'HYPK'
1826 'TRIM34'
1827 'DENND5A'
1828 'ZNF28'
1829 'TTL'
1830 'FERMT1'
1831 'SLC46A1'
1832 'KIAA1107'
1833 'ZFAND2A'
1834 'PHLDA1'
1835 'BAG3'
1836 'RGMB'
1837 'METRNL'
1838 'MTA3'
1839 'ARSK'
1840 'RNPEPL1'
1841 'EPOP'
1842 'ABCC10'
1843 'MSX1'
1844 'DDIT3'
1845 'VAMP2'
1846 'MTRNR2L6'

1847 'AJUBA'
1848 'TULP4'
1849 'BLVRB'
1850 'MTMR10'
1851 'CORO7-PAM
1852 'HIST1H4H'
1853 'LRMDA'
1854 'CRNKL1'
1855 'GSTT1'
1856 'OLA1'
1857 'LOC1079855
1858 'PTAR1'
1859 'MTCH2'
1860 'NUDT18'
1861 'BCAS3'
1862 'RPP40'
1863 'TRIM68'
1864 'DPAGT1'
1865 'ZNF888'
1866 'HYAL1'
1867 'ZNF274'
1868 'PABPC1L'
1869 'LOC1019298
1870 'YJEFN3'
1871 'CD55'
1872 'CYP20A1'
1873 'JMY'
1874 'HRASLS'
1875 'SAMD9'
1876 'SUGCT'
1877 'FZD4'
1878 'HLA-B'
1879 'TMEM120B'
1880 'ATPAF2'
1881 'ABTB1'
1882 'ALDH7A1'
1883 'NPIPB12'
1884 'ZNF350'
1885 'TTC17'
1886 'TMEM223'
1887 'STK19'
1888 'PGM2L1'
1889 'LRRC28'
1890 'MED17'
1891 'LY6G5B'
1892 'ELK4'
1893 'WNT3'
1894 'ALOX5AP'
1895 'SLC36A4'
1896 'CTSB'
1897 'POLR3F'
1898 'NDUFA10'
1899 'ANKRD49'
1900 'PCNX4'
1901 'DDRGK1'
1902 'RAB29'

1903	'COL9A2'
1904	'OXTR'
1905	'PIK3C2A'
1906	'FUT10'
1907	'LGALS3BP'
1908	'NDUFS1'
1909	'CWC15'
1910	'ZNF70'
1911	'PANX2'
1912	'PACS2'
1913	'MRPS5'
1914	'ABCC5'
1915	'TOGARAM1'
1916	'LOC1053794
1917	'CEMIP2'
1918	'RBM24'
1919	'PHACTR2'
1920	'MARCH8'
1921	'MFF'
1922	'HECW1'
1923	'APLP2'
1924	'AASDHPPT'
1925	'FAM126B'
1926	'SLC25A42'
1927	'CLIP4'
1928	'MAMDC2'
1929	'HLA-C'
1930	'BTBD3'
1931	'MT2A'
1932	'ZC3H12C'
1933	'FCHSD1'
1934	'ABCC3'
1935	'DIP2C'
1936	'SIK3'
1937	'PLD3'
1938	'CERS6'
1939	'USP40'
1940	'100133301'
1941	'SMIM31'
1942	'RASGRP2'
1943	'SPON2'
1944	'LOC1053737
1945	'AVIL'
1946	'FTCD'
1947	'C17orf113'
1948	'GALNT6'
1949	'SERPINA3'
1950	'SPATA32'
1951	'KLF17'
1952	'CPNE4'
1953	'CSF2RB'
1954	'IGSF11'
1955	'SHISA3'
1956	'USP51'
1957	'DNAH6'
1958	'DNAH10'

1959 'DTX3'
1960 'ELAVL3'
1961 'TFEC'
1962 'ADGRL3'
1963 'KIAA1549L'
1964 'SHC2'
1965 'QPCT'
1966 'IFT172'
1967 'GIPR'
1968 'GPX3'
1969 'CD209'
1970 'AOC2'
1971 'ONECUT1'
1972 'HOXC13'
1973 'SNAI3'
1974 'HTR2C'
1975 'LRRC66'
1976 'AQP7'
1977 'KCNJ1'
1978 'KCNJ2'
1979 'KCNJ13'
1980 'KCNN3'
1981 'KCNQ3'
1982 'KRT83'
1983 'ANKRD20A3
1984 'NTRK2'
1985 'MLXIPL'
1986 'CEND1'
1987 'FXYD6'
1988 'POU3F2'
1989 'NECAB2'
1990 'PPL'
1991 'DNAH3'
1992 'HR'
1993 'TSNAXIP1'
1994 'PCDHGC4'
1995 'CPXM1'
1996 'CEMIP'
1997 'WDFY4'
1998 'SEMA4G'
1999 'CACNG7'
2000 'FNDC10'
2001 'CDH22'
2002 'SYT5'
2003 'LAT2'
2004 'ZNF221'
2005 'TMC5'
2006 'PLEKHS1'
2007 'GREB1L'
2008 'PGAP1'
2009 'CAMK2B'
2010 'AMN'
2011 'MADCAM1'
2012 'SCRT1'
2013 'SLC25A18'
2014 'AOC3'

2015 'CHRD'
2016 'ENDOU'
2017 'CACNA1I'
2018 'CACNA1G'
2019 'CD1D'
2020 'ZNF804A'
2021 'OLFM2'
2022 'SYTL5'
2023 'SPOCK2'
2024 'JAKMIP2'
2025 'ICAM1'
2026 'EIF1AD'
2027 'TMEM178B'
2028 'IWS1'
2029 'ARHGAP12'
2030 'EXTL3'
2031 'DDX60L'
2032 'DNMT3B'
2033 'C20orf194'
2034 'ASB6'
2035 'FDX1'
2036 'LDLRAD3'
2037 'RAB3B'
2038 'ABI2'
2039 'MAPK7'
2040 'ZNF234'
2041 'PTCH1'
2042 'TMEM230'
2043 'CHIC1'
2044 'ARHGAP6'
2045 'NANP'
2046 'MTRNR2L8'
2047 'ATRN'
2048 'STK25'
2049 'TMOD2'
2050 'MAVS'
2051 'SCRN1'
2052 'CYB5D1'
2053 'PGM2'
2054 'EFL1'
2055 'RRAS2'
2056 'AASS'
2057 'KLHL15'
2058 'ARCN1'
2059 'CARF'
2060 'ACP6'
2061 'GPC1'
2062 'SSB'
2063 'ZNF805'
2064 'DUSP8'
2065 'SLC35A1'
2066 'ATP10D'
2067 'TRAPPC4'
2068 'APMAP'
2069 'SGPL1'
2070 'PCOLCE2'

2071 'DACT3'
2072 'NBEA'
2073 'IFI16'
2074 'ANKS6'
2075 'SNRPB2'
2076 'CTSO'
2077 'KAT5'
2078 'C15orf65'
2079 'CMTM8'
2080 'GAN'
2081 'TBCE'
2082 'PLEKHA4'
2083 'LYRM9'
2084 'SLC22A11'
2085 'BUD13'
2086 'CIR1'
2087 'MGME1'
2088 'ITPRID2'
2089 'METAP1D'
2090 'TALDO1'
2091 'LDHC'
2092 'SHF'
2093 'ADARB1'
2094 'PTPRA'
2095 'MAIP1'
2096 'MSN'
2097 'FUT4'
2098 'SYTL4'
2099 'RBM7'
2100 'EXD2'
2101 'STAM2'
2102 'TBX19'
2103 'DENND5B'
2104 'LIMS4'
2105 'ADGRG1'
2106 'ATP5MG'
2107 'ZNF543'
2108 'EVI5'
2109 'RPL17-C18o
2110 'NIPAL4'
2111 'TBCK'
2112 'CCDC122'
2113 'PLSCR3'
2114 'MMP16'
2115 'HSPBAP1'
2116 'PMP22'
2117 'KDM4D'
2118 'OGDH'
2119 'FAM169A'
2120 'RIMBP3B'
2121 'CEP89'
2122 'GPD2'
2123 'HLA-A'
2124 'PIKFYVE'
2125 'PAX6'
2126 'PCYOX1'

2127 'DNHD1'
2128 'SLC29A4'
2129 'LHFPL2'
2130 'HOXD11'
2131 'EDIL3'
2132 'SPATA17'
2133 'FKRP'
2134 'ZBTB47'
2135 'RTN3'
2136 'SLC35G1'
2137 'TBC1D30'
2138 'CTSK'
2139 'SLC7A6'
2140 'BHLHB9'
2141 'GABBR1'
2142 'CASP8'
2143 'IDH1'
2144 'HECW2'
2145 'MDGA2'
2146 'MTRNR2L10
2147 'ZBTB49'
2148 'TLR1'
2149 'PRDM8'
2150 'AURKC'
2151 'NATD1'
2152 'ANAPC1'
2153 'SMIM29'
2154 'PANK2'
2155 'ZNF600'
2156 'MRE11'
2157 'CDCP1'
2158 'BTBD9'
2159 'UBE2Q2'
2160 'TMEM179B'
2161 'PSEN1'
2162 'DCUN1D5'
2163 'ZBTB42'
2164 'COMMD1'
2165 'CYB5R1'
2166 'TXNDC9'
2167 'ARPC4-TTLL
2168 'ALCAM'
2169 'EMB'
2170 'ZNF681'
2171 'HOXD1'
2172 'TIMP2'
2173 'LOC1079842
2174 'IL6ST'
2175 'SPICE1'
2176 'NUDT17'
2177 'ZNF121'
2178 'ANKLE1'
2179 'ZKSCAN4'
2180 'KIF4A'
2181 'PPP1R21'
2182 'TMEM170B'

2183 'LGALS8'
2184 'HBEGF'
2185 'MYL12A'
2186 'TAF1B'
2187 'FIGN'
2188 'PCDHGB1'
2189 'BCL9L'
2190 'PPT2'
2191 'TUBB6'
2192 'IGF2R'
2193 'CENPBD1'
2194 'TANK'
2195 'SAMD8'
2196 'TRIM3'
2197 'RAP1GDS1'
2198 'UQCRFS1'
2199 'IL7'
2200 'HDAC5'
2201 'ASNSD1'
2202 'TJP2'
2203 'FOXRED1'
2204 'ARID5B'
2205 'FAM84B'
2206 'RBM45'
2207 'NFE2L3'
2208 'DNAH14'
2209 'SNX5'
2210 'CSPG4'
2211 'ZNF337'
2212 'ZNF527'
2213 'ZBTB34'
2214 'AAMDC'
2215 'DCTD'
2216 'ATXN3'
2217 'XRN2'
2218 'CNTNAP3C'
2219 'CNP'
2220 'KCNJ8'
2221 'GLS'
2222 'PCDHB10'
2223 'TRAF6'
2224 'DND1'
2225 'INSIG2'
2226 '100996741'
2227 'GPR155'
2228 'SAV1'
2229 'NT5C3A'
2230 'KDM5B'
2231 'TDRKH'
2232 'HIST2H4B'
2233 'HIST2H4A'
2234 'THY1'
2235 'GPR153'
2236 'DTWD2'
2237 'ZNRF1'
2238 'MEX3B'

2239 'ZFYVE1'
2240 'MFSD6'
2241 'MOSPD2'
2242 'RP2'
2243 'TNFSF13'
2244 'PTPN21'
2245 'PIR'
2246 'ZNF280C'
2247 'CCNH'
2248 'CCDC17'
2249 'PTRHD1'
2250 'RPS4X'
2251 'B2M'
2252 'EFNA3'
2253 'FAM172A'
2254 'STAT2'
2255 'KSR1'
2256 'NEPRO'
2257 'ZNF773'
2258 'DNAJB4'
2259 'DDAH1'
2260 'TUBG2'
2261 'SEC23B'
2262 'LAMB2'
2263 'F2RL2'
2264 'HS6ST3'
2265 'ZNF30'
2266 'EFHB'
2267 'SLC25A45'
2268 'NDUFS4'
2269 'POLB'
2270 'SLC18B1'
2271 'ZNF155'
2272 'MMADHC'
2273 'VPS13C'
2274 'CCNJL'
2275 'ALKBH8'
2276 'MTLN'
2277 'NBEAL1'
2278 'RGPD4'
2279 'JMJD7-PLA2
2280 'ARSD'
2281 'TTC5'
2282 'CDKAL1'
2283 'RGL1'
2284 'PCLO'
2285 'GPC4'
2286 'HPS1'
2287 'GPT2'
2288 'SCHIP1'
2289 'RAI14'
2290 'DNMBP'
2291 'ACOT2'
2292 'SARS'
2293 'NHEJ1'
2294 'BST1'

2295 'SLC30A1'
2296 'SLC44A3'
2297 'MED19'
2298 'RRBP1'
2299 'GRHL1'
2300 'PBXIP1'
2301 'MTRNR2L9'
2302 'CWC27'
2303 'MPZL2'
2304 'HIST1H3D'
2305 'AARSD1'
2306 'NEIL3'
2307 'ATL3'
2308 'TMEM106A'
2309 'RHOT1'
2310 'NAA40'
2311 'C11orf58'
2312 'SNRPN'
2313 'CDK18'
2314 'CLMP'
2315 'ZNF567'
2316 'ORC3'
2317 'HCFC2'
2318 'SDHAF2'
2319 'NABP1'
2320 'BCLAF3'
2321 'KCNJ14'
2322 'RAB20'
2323 'BLCAP'
2324 'ZNF749'
2325 'CCDC97'
2326 'KLHL28'
2327 'RNF166'
2328 'SYVN1'
2329 'NAPB'
2330 'RPE'
2331 'STK35'
2332 'HOMER3'
2333 'ING5'
2334 'LZTS3'
2335 'DTNA'
2336 'RHOBTB3'
2337 'FAM184A'
2338 'AOX1'
2339 'EXOC6B'
2340 'DOK1'
2341 'TIAF1'
2342 'UCN'
2343 'EID2'
2344 'S1PR2'
2345 'FAM89B'
2346 'GRB10'
2347 'NECTIN1'
2348 'WDR75'
2349 'METTL21A'
2350 'SPG21'

2351 'STK36'
2352 'PDCD2L'
2353 'ERCC1'
2354 'EML2'
2355 'COPS8'
2356 'RIMBP3'
2357 'RDH10'
2358 'CAT'
2359 'ATP6V1D'
2360 'C6orf203'
2361 'MGAT5B'
2362 'SETD9'
2363 'LOC1079861
2364 'ADAMTSL5'
2365 'ZGLP1'
2366 'TTC34'
2367 'TMC4'
2368 'F5'
2369 'ZDHHC23'
2370 'GPLD1'
2371 'IL2RB'
2372 'FBLIM1'
2373 'MAPK13'
2374 'BGN'
2375 'SCG5'
2376 'FAM227A'
2377 'LRFN4'
2378 'ESAM'
2379 'TNFSF15'
2380 'MAPRE2'
2381 'NUFIP2'
2382 'BRMS1L'
2383 'SPPL2A'
2384 'ZC3H15'
2385 'ZNF512B'
2386 'OTUD1'
2387 'BTD'
2388 'LOC1122682
2389 'GBP3'
2390 'KLF7'
2391 'CLPX'
2392 'LOC1079873
2393 'ZSWIM9'
2394 'MTRF1L'
2395 'GPR137'
2396 'SCAPER'
2397 'GTF2E2'
2398 'CLCN4'
2399 'GABPB2'
2400 'GSK3B'
2401 'ZNF490'
2402 'ZNF382'
2403 'TAPT1'
2404 'TOB1'
2405 'CFL2'
2406 'CCDC186'

2407 'ANKRD12'
2408 'SLC31A2'
2409 'HDAC8'
2410 'PAM'
2411 'ZNF765-ZNF
2412 'ZNF625'
2413 'GLRX'
2414 'ANKRD23'
2415 'PRSS23'
2416 'ZNF613'
2417 'PARVA'
2418 'HOXA11'
2419 'HCCS'
2420 'AMPD3'
2421 'NPTXR'
2422 'AP1S2'
2423 'CEP97'
2424 'ROBO1'
2425 'HEXB'
2426 'VAV2'
2427 'SCFD1'
2428 'ACTBL2'
2429 'ZFP91'
2430 'NOL3'
2431 'SLC14A2'
2432 'SNURF'
2433 'ATG16L1'
2434 'MXD4'
2435 'MAP1B'
2436 'C12orf49'
2437 'COL6A2'
2438 'CHAC2'
2439 'ANXA2'
2440 'ZNF484'
2441 'EVI5L'
2442 'SLC38A7'
2443 'C2orf74'
2444 'HEXA'
2445 'MTMR9'
2446 'NEDD8-MDF
2447 'MEGF11'
2448 'SCARF1'
2449 'RBPJ'
2450 'TRAPPC11'
2451 'KLHL36'
2452 'ZNF419'
2453 'HINT3'
2454 'ULK3'
2455 'PCSK4'
2456 'EZH1'
2457 'CSNK2A1'
2458 'PPP2R5B'
2459 'CTSH'
2460 'PDSS2'
2461 'FRMD8'
2462 'PHLDA2'

2463 'CWC22'
2464 'PRR16'
2465 'ADORA1'
2466 'GPRC5A'
2467 'OR7E24'
2468 'SNRPB'
2469 'PTPN4'
2470 'WDR44'
2471 'API5'
2472 'FAM234B'
2473 'ZNF224'
2474 'ZMYM3'
2475 'UPRT'
2476 'AP4S1'
2477 'ACAT1'
2478 'VPS37C'
2479 'PLEKHM1'
2480 'SIN3B'
2481 'FUT11'
2482 'ADHFE1'
2483 'ZNF763'
2484 'ZNF284'
2485 'SLC17A7'
2486 'TTLL7'
2487 'TTYH2'
2488 'REEP6'
2489 'GNL3L'
2490 'MTFMT'
2491 'GTPBP8'
2492 'NIPAL3'
2493 'CYSRT1'
2494 'FOXN2'
2495 'SNX16'
2496 'TIMM10B'
2497 'LIME1'
2498 'ZNF292'
2499 'RNF187'
2500 'OSBPL10'
2501 'SLC23A3'
2502 'PTPRB'
2503 'BNIP3'
2504 'LOC1122679
2505 'TMEM138'
2506 'CALCOCO2'
2507 'KDM3A'
2508 'CPEB2'
2509 'PM20D2'
2510 'SEC31B'
2511 'SNX25'
2512 'SRPRB'
2513 'SAAL1'
2514 'COL4A3BP'
2515 'MID1'
2516 'ERBIN'
2517 'MYL12B'
2518 'OPRL1'

2519 'COL8A1'
2520 'SUPT3H'
2521 'KBTBD3'
2522 'CLPTM1'
2523 'CYP46A1'
2524 'THBS4'
2525 'CASC10'
2526 'NAA20'
2527 'ZBTB43'
2528 'USP9X'
2529 'FAM156A'
2530 'AGO3'
2531 'RAD17'
2532 'ZXDB'
2533 'FBXW10'
2534 'NR4A3'
2535 'PPFIA4'
2536 'LAP3'
2537 'RASA2'
2538 'TSPAN10'
2539 'DMGDH'
2540 'MTRNR2L2'
2541 'BORCS8'
2542 'MSS51'
2543 'LRRC15'
2544 'TCP11L2'
2545 'DERL2'
2546 'NIPSNAP2'
2547 'TMEM63A'
2548 'GCAT'
2549 'SOS2'
2550 'DMPK'
2551 'TTC12'
2552 'UBXN4'
2553 'ZNF565'
2554 'CCRL2'
2555 'ZEB1'
2556 'ACTN1'
2557 'SPIN3'
2558 'SPANXB1'
2559 'FAH'
2560 'RUNDC1'
2561 'TMEM106B'
2562 'ITPA'
2563 'FARP1'
2564 'ANKRD33B'
2565 'FITM2'
2566 'PDHX'
2567 'L3HYPDH'
2568 'STEAP2'
2569 'TRIM35'
2570 'ZFP90'
2571 'ANGPTL2'
2572 'MAGI2'
2573 'MAPKBP1'
2574 'GADD45B'

2575 'LOC391322'
2576 'PGK1'
2577 'HIST1H2AG'
2578 'EXT2'
2579 'BIRC2'
2580 'SMIM10'
2581 'ARHGAP4'
2582 'ONECUT2'
2583 'PRRG1'
2584 'GLA'
2585 'ZNF595'
2586 'PRR5'
2587 'MINDY3'
2588 'ALS2CL'
2589 'MANBA'
2590 'TSPAN14'
2591 'CACNB1'
2592 'OGA'
2593 'ANXA6'
2594 'FYCO1'
2595 'ZNF784'
2596 'MYL5'
2597 'CD83'
2598 'ZNF397'
2599 'LCORL'
2600 'IQSEC1'
2601 'PREP'
2602 'ZNF215'
2603 'GIGYF2'
2604 'TECPR2'
2605 'HSD11B1L'
2606 'INTS5'
2607 'ARVCF'
2608 'DPP7'
2609 'XRCC5'
2610 'SH3BGRL'
2611 'ITM2B'
2612 'FMNL1'
2613 'ZNF383'
2614 'UTP15'
2615 'SERTAD1'
2616 'ZNF17'
2617 'FOXD1'
2618 'YPEL5'
2619 'FAM222B'
2620 'FAM214A'
2621 'SHISA4'
2622 'SCAMP5'
2623 'GEM'
2624 'ULBP3'
2625 'LIMD1'
2626 'LOC1053762
2627 'LOC1122683
2628 'ZNF474'
2629 'CARMIL2'
2630 'ARX'

2631 'NLRC3'
2632 'KIAA0825'
2633 'KLKB1'
2634 'MAOB'
2635 'PDE3A'
2636 'GRAMD1C'
2637 'MREG'
2638 'ERMARD'
2639 'PTGIS'
2640 'BACH2'
2641 'ISL2'
2642 'SEH1L'
2643 'SIPA1'
2644 'TAF13'
2645 'POLM'
2646 'C6orf89'
2647 'KLHL26'
2648 'ADRA1B'
2649 'SLC38A9'
2650 'GOLGA8A'
2651 'POLR2G'
2652 'MARCH7'
2653 'SKIV2L'
2654 'MRPL17'
2655 'GOLGA8H'
2656 'SPATA20'
2657 'CYR61'
2658 'PTRH1'
2659 'FAM162A'
2660 'CASC3'
2661 'AP5M1'
2662 'DHX29'
2663 'TBC1D13'
2664 'MNAT1'
2665 'TJP1'
2666 'ZNF417'
2667 'IL12A'
2668 'SLC39A10'
2669 'HDAC6'
2670 'COQ10B'
2671 'RBBP9'
2672 'TP53INP2'
2673 'CGRRF1'
2674 'TRAPPC13'
2675 'FBXL20'
2676 'RDH13'
2677 'ASF1A'
2678 'EIF3CL'
2679 'RHPN2'
2680 'IMPA2'
2681 'SLC45A4'
2682 'SLC22A15'
2683 'TMEM42'
2684 'CAVIN1'
2685 'PPP2CB'
2686 'VPS16'

2687 'NOTCH2'
2688 'MORF4L1'
2689 'CALCOCO1'
2690 'CSAD'
2691 'ZNF211'
2692 'ACTR3'
2693 'PARP8'
2694 'ANKHD1'
2695 'WDR12'
2696 'SYNRG'
2697 'LASP1'
2698 'NUDT16'
2699 'TOX4'
2700 'BMP8A'
2701 'PCDHB14'
2702 'BCL2L13'
2703 'TOR4A'
2704 'DNAAF4'
2705 'FRMD3'
2706 'CEP85L'
2707 'AKAP6'
2708 'IRAK1BP1'
2709 'SIRPA'
2710 'PLCB4'
2711 'SRR'
2712 'CYB5R4'
2713 'SMAP1'
2714 'NCL'
2715 'PLBD2'
2716 'NPIPB2'
2717 'FCHO1'
2718 'RFESD'
2719 'ARSB'
2720 'PADI3'
2721 'DDX52'
2722 'VPS13A'
2723 'BCAT2'
2724 'EMC7'
2725 'RBM43'
2726 'CTAGE4'
2727 'DUSP5'
2728 'DAB2'
2729 'ZNF75A'
2730 'SETX'
2731 'CCDC138'
2732 'AP2A2'
2733 'GPR162'
2734 'ATP1A2'
2735 'ZNF827'
2736 'COPB1'
2737 'LIN7B'
2738 'PML'
2739 'ADPRM'
2740 'SYNC'
2741 'ZNFX1'
2742 'LCN2'

2743 'ADAMTSL3'
2744 'SMPD1'
2745 'SMIM14'
2746 'ZNF91'
2747 'MYLIP'
2748 'NFIX'
2749 'CAPN10'
2750 'R3HCC1'
2751 'EEFSEC'
2752 'NEMF'
2753 'FAM76B'
2754 'DCBLD1'
2755 'CFAP97'
2756 'CCDC103'
2757 'TMOD3'
2758 'EPB41L5'
2759 'PCM1'
2760 'PACS1'
2761 'JARID2'
2762 'TMEM182'
2763 'RABL2A'
2764 'HOXC10'
2765 'KSR2'
2766 'SEC14L4'
2767 'TMEM191B'
2768 'FAM131B'
2769 'PIGS'
2770 'RAB18'
2771 'PAFAH1B2'
2772 'ARRB2'
2773 'FAM135A'
2774 'PIP5K1C'
2775 'TSGA10'
2776 'UBE2D4'
2777 'THRA'
2778 'DOK3'
2779 'ROCK2'
2780 'SLC9A6'
2781 'RAP1GAP2'
2782 'IRF2BP1'
2783 'DLX4'
2784 'BAG4'
2785 'ZNF304'
2786 'FAM229A'
2787 'FRAT1'
2788 'RFK'
2789 'SMURF2'
2790 'C6orf226'
2791 'EPB41L2'
2792 'FICD'
2793 'C20orf27'
2794 'C1orf56'
2795 'ZNF841'
2796 'AGGF1'
2797 'ACYP2'
2798 'TSPAN6'

2799 'SH3BP2'
2800 'SLC1A1'
2801 'LPAR2'
2802 'RNF19A'
2803 'CGAS'
2804 'PTPN2'
2805 'ACTR5'
2806 'FASTKD5'
2807 'PSAP'
2808 'FAM155A'
2809 'FIBCD1'
2810 'ZNF532'
2811 'SEMA4C'
2812 'DHRS13'
2813 'BTN2A2'
2814 'COQ5'
2815 'ELL2'
2816 'ARMCX5'
2817 'ZNF426'
2818 'CFL1'
2819 'CCDC115'
2820 'LOC105372[illegible]
2821 'SRGAP3'
2822 'GCH1'
2823 'LRRC24'
2824 'HGSNAT'
2825 'SUMO1'
2826 'CHST7'
2827 'CEBPZ'
2828 'IPMK'
2829 'SMN1'
2830 'FKBP1A'
2831 'BCORL1'
2832 'BAZ2B'
2833 'MKKS'
2834 'MED27'
2835 'PCDHB9'
2836 'NOP56'
2837 'GSTM4'
2838 'H1FX'
2839 'LOC100652[illegible]
2840 'LTB4R'
2841 'CYFIP2'
2842 'ERMP1'
2843 'SCML2'
2844 'SEPT2'
2845 'SUZ12'
2846 'ZNF473'
2847 'MCTP1'
2848 'GNAQ'
2849 'TRAK2'
2850 'CINP'
2851 'NOTCH2NL[illegible]
2852 'MAP2K3'
2853 'LPP'
2854 'ZNF652'

2855	'TMEM64'
2856	'ZBTB25'
2857	'C16orf46'
2858	'DDN'
2859	'SSC5D'
2860	'ZNF343'
2861	'RPGR'
2862	'SERGEF'
2863	'PDP1'
2864	'FBXW7'
2865	'LOC1079870
2866	'ZNF654'
2867	'ITGB3'
2868	'CCDC96'
2869	'PIK3CD'
2870	'FBXO30'
2871	'TMEM267'
2872	'ARHGEF2'
2873	'ITGAV'
2874	'CLP1'
2875	'CAST'
2876	'PIGH'
2877	'GAS2L1'
2878	'ATP6V1G1'
2879	'SPTLC3'
2880	'PIK3R3'
2881	'BCAT1'
2882	'ACTN4'
2883	'C11orf71'
2884	'HEATR5A'
2885	'UQCC2'
2886	'STK40'
2887	'DPF1'
2888	'DHFR2'
2889	'IBA57'
2890	'ERMAP'
2891	'CBX7'
2892	'ANKRD46'
2893	'ZNF222'
2894	'ZNF57'
2895	'FASTKD1'
2896	'TNFSF4'
2897	'AGAP9'
2898	'STIP1'
2899	'TRIM38'
2900	'IGF2BP3'
2901	'AREL1'
2902	'FAM129B'
2903	'FBXL7'
2904	'MAP4K4'
2905	'TMX4'
2906	'ZNF79'
2907	'MED29'
2908	'SAMD4A'
2909	'ZNF765'
2910	'LYRM2'

2911	'FEM1B'
2912	'YOD1'
2913	'TP53BP1'
2914	'DOCK6'
2915	'CTSA'
2916	'ITPRIP'
2917	'LMCD1'
2918	'LOC1079874
2919	'ITGA2'
2920	'IL21R'
2921	'VNN1'
2922	'ADAMTS4'
2923	'CST3'
2924	'POLR2L'
2925	'H6PD'
2926	'TIMM17B'
2927	'CADM1'
2928	'UBE3D'
2929	'DTD2'
2930	'CLDN1'
2931	'PLK2'
2932	'EXOC5'
2933	'LOC1053795
2934	'ZBTB20'
2935	'PRMT9'
2936	'ZNF264'
2937	'CHRNB1'
2938	'PIK3R1'
2939	'CYP2D7'
2940	'ZNF223'
2941	'HOXA10'
2942	'CMTM1'
2943	'ZNF594'
2944	'AKIRIN2'
2945	'NNMT'
2946	'MXI1'
2947	'LIMA1'
2948	'MCIDAS'
2949	'ZNF462'
2950	'CRKL'
2951	'CCDC102A'
2952	'TRPS1'
2953	'DHRS12'
2954	'MBNL2'
2955	'TBL1X'
2956	'MRPS27'
2957	'HES4'
2958	'AIF1L'
2959	'GM2A'
2960	'CDA'
2961	'SLC35E2A'
2962	'TRMT5'
2963	'ZIC5'
2964	'KIAA0100'
2965	'STARD3NL'
2966	'NUDCD3'

2967 'CFAP206'
2968 'PIPOX'
2969 'PTPRK'
2970 'ATXN1L'
2971 'TSPAN2'
2972 'TNRC6B'
2973 'BCOR'
2974 'DPY19L3'
2975 'ASPH'
2976 'HELZ'
2977 'TBCB'
2978 'SLFN5'
2979 'RIN2'
2980 'MAP2K5'
2981 'ZNF669'
2982 'KDM6B'
2983 'GTF2A1'
2984 'PSME3'
2985 'RNF145'
2986 'ZNF507'
2987 'NDC80'
2988 'RNF208'
2989 'LRRC37A'
2990 'ABCE1'
2991 'DMXL2'
2992 'LOC1019303
2993 'INPP5E'
2994 'TMEM88'
2995 'KRT18'
2996 'SNX3'
2997 'KAT14'
2998 'TENM4'
2999 'NOP9'
3000 'BTBD19'
3001 'DYNC1H1'
3002 'PPWD1'
3003 'NUDT14'
3004 'LYSMD3'
3005 'ADAMTSL1'
3006 'SNX14'
3007 'TRIM2'
3008 'ADGRA2'
3009 'MST1'
3010 'C8G'
3011 'ARHGEF1'
3012 'BAK1'
3013 'SPTAN1'
3014 'LOC1079846
3015 'PCDHGA6'
3016 'PODNL1'
3017 'CMTR2'
3018 'CNN3'
3019 'PBLD'
3020 'SKA2'
3021 'ZNF845'
3022 'NSF'

3023 'LY96'
3024 'ENO3'
3025 'VWCE'
3026 'MMP19'
3027 'TKFC'
3028 'LRP1'
3029 'MANEA'
3030 'LAMC2'
3031 'TP63'
3032 'TGFB2'
3033 'TFPI'
3034 'CCT6B'
3035 'ZNF227'
3036 'SFXN3'
3037 'KLHL22'
3038 'BRD3OS'
3039 'DHODH'
3040 'MAN2B2'
3041 'ARL17A'
3042 'CLSTN1'
3043 'NAXE'
3044 'ZNF526'
3045 'RIOX1'
3046 'PINX1'
3047 'PDCD1LG2'
3048 'SAR1A'
3049 'PANO1'
3050 'TBC1D25'
3051 'PPARA'
3052 'SMARCA1'
3053 'INKA2'
3054 'KDELR3'
3055 'ZNF175'
3056 'WDR54'
3057 'NUDT11'
3058 'TANC1'
3059 'CD300C'
3060 'COL13A1'
3061 'LRRN4CL'
3062 'ENPP4'
3063 'TMEM121B'
3064 'ZNF547'
3065 'EPHA10'
3066 'KCNN1'
3067 'PRKAB2'
3068 'CATSPERG'
3069 'RASSF10'
3070 'WAS'
3071 'AKAP2'
3072 'ZNF71'
3073 'STK10'
3074 'MTREX'
3075 'NRARP'
3076 'SMAD6'
3077 'TRAPPC6B'
3078 'SLC35A5'

3079 'ZSWIM6'
3080 'TMEM189'
3081 'LBHD1'
3082 'ATF7IP'
3083 'NDST2'
3084 'ZNF550'
3085 'LOC1053733
3086 'DENND2C'
3087 'MAMSTR'
3088 'TLN2'
3089 'ZCCHC3'
3090 'NUTM2E'
3091 'ZNF708'
3092 'CPED1'
3093 'HELB'
3094 'PSMC4'
3095 'GOLGA2'
3096 'KLC1'
3097 'PUDP'
3098 'DOCK4'
3099 'GOLGA8B'
3100 'RTL8B'
3101 'NUTM2A'
3102 'NXNL2'
3103 'APH1B'
3104 'BEX3'
3105 'SPRY3'
3106 'MIF4GD'
3107 'MSH3'
3108 'SYCE2'
3109 'ESR1'
3110 'C11orf74'
3111 'ALDH5A1'
3112 'NDUFB1'
3113 'LOC1079865
3114 'TENT5A'
3115 'PXDN'
3116 'ARMH4'
3117 'CPLANE1'
3118 'DGKQ'
3119 'CDKN2AIP'
3120 'KRTAP2-1'
3121 'AKTIP'
3122 'MRPS21'
3123 'HDHD2'
3124 'FRK'
3125 'EFCAB5'
3126 'NIFK'
3127 'AK3'
3128 'ZNF658'
3129 'LRIF1'
3130 'LOC1079839
3131 'HDAC10'
3132 'ARL6'
3133 'INTS9'
3134 'CLHC1'

3135 'RNGTT'
3136 'HSD17B10'
3137 'TIGD7'
3138 'HOXB3'
3139 'BICRAL'
3140 'HDDC3'
3141 'LRRN4'
3142 'GPSM1'
3143 'MYO18A'
3144 'NFAT5'
3145 'ITGB1'
3146 'CD151'
3147 'LOC1001303
3148 'TLR6'
3149 'AGAP11'
3150 'SLC43A2'
3151 'DNM3'
3152 'RBMS3'
3153 'COQ6'
3154 'HIST2H2AA4
3155 'HIST2H2AA3
3156 'PKD2'
3157 'PRUNE2'
3158 'MCCC2'
3159 'DPY19L2'
3160 'BID'
3161 'GPR157'
3162 'ERLIN2'
3163 'HTT'
3164 'ARAF'
3165 'MGAT2'
3166 'ORMDL3'
3167 'GCLM'
3168 'ENTPD4'
3169 'EDN1'
3170 'FARP2'
3171 'SUPT6H'
3172 'TRIM45'
3173 'SLC41A3'
3174 'ZNF66'
3175 'BAG2'
3176 'CSRP1'
3177 '107985971'
3178 'PDK1'
3179 'B3GNT9'
3180 'USB1'
3181 'C15orf61'
3182 'TTLL5'
3183 'NAT1'
3184 'DTNB'
3185 'SIPA1L3'
3186 'RIT1'
3187 'SULF1'
3188 'SPOCK3'
3189 'CNFN'
3190 'RPL36A-HNI

3191 'CAPNS1'
3192 'NPIPA8'
3193 'ERAP2'
3194 'SELENON'
3195 'SNAPC4'
3196 'CBX6'
3197 'MACROD1'
3198 'RBM18'
3199 'TRIB1'
3200 'ANKMY2'
3201 'NF2'
3202 'PPP1R18'
3203 'GLMN'
3204 'ZNF555'
3205 'ZNF788P'
3206 'IQSEC2'
3207 'TMEM175'
3208 'ZSCAN21'
3209 'HHIPL2'
3210 'SPIN2B'
3211 'ZNF529'
3212 'PRKD3'
3213 'MOCS2'
3214 'CCDC22'
3215 'BRF1'
3216 'CTNNBIP1'
3217 'TTC13'
3218 'MAP2K1'
3219 'EIF2AK4'
3220 'HDLBP'
3221 'CTSZ'
3222 'SIDT2'
3223 'CRLS1'
3224 'DPH3'
3225 'SERF1B'
3226 'ZNF562'
3227 'CEBPB'
3228 'DYNLT3'
3229 'DUSP14'
3230 'COX6B1'
3231 'ZDHHC2'
3232 'NIPAL1'
3233 'SHH'
3234 'TRAPPC2B'
3235 'PDLIM1'
3236 'SPOUT1'
3237 'ITSN1'
3238 'SH3BP5L'
3239 'KCTD11'
3240 'ZBTB1'
3241 'ZNF34'
3242 'ZNF516'
3243 'ODF2L'
3244 'CYLD'
3245 'AP2M1'
3246 'SYCP2L'

3247 'RRAS'
3248 'SEL1L'
3249 'EIF3K'
3250 'PKIA'
3251 'CCL26'
3252 'FCRLB'
3253 'SLX4IP'
3254 'VPS37D'
3255 'FHL3'
3256 'WDR7'
3257 'DCDC1'
3258 'TGM1'
3259 'ELL'
3260 'CTH'
3261 'EXOSC6'
3262 'KRTAP2-3'
3263 'ZNF611'
3264 'SEC22B'
3265 'STARD13'
3266 'ACSF2'
3267 'ZC3H14'
3268 'FAM228B'
3269 'RTL10'
3270 'CPEB4'
3271 'ZNF675'
3272 'PFKFB3'
3273 'ZNF778'
3274 'ACAD11'
3275 'CCDC92B'
3276 'ZNF253'
3277 'RIC3'
3278 'NUTM2D'
3279 'C6orf120'
3280 'GADD45A'
3281 'MICALL2'
3282 'GLIPR1'
3283 'ATP6AP2'
3284 'TMSB4X'
3285 'TMEM131L'
3286 'ZFP62'
3287 'SAP25'
3288 'ELMO3'
3289 'ATF3'
3290 'TRIM39-RPF
3291 'NBPF1'
3292 'ZNF510'
3293 'WDR89'
3294 'ABCA13'
3295 'HK1'
3296 'ARPC5L'
3297 'PDK2'
3298 'ZNF860'
3299 'HOXD9'
3300 'UNC13B'
3301 'AKAP8L'
3302 'ROCK1'

3303 'CHD7'
3304 'ASAP1'
3305 'TMEM59'
3306 'LOC1122683
3307 'SENP8'
3308 'OTUD5'
3309 'FRMD6'
3310 'TMSB15B'
3311 'GAPVD1'
3312 'CITED2'
3313 'CDRT4'
3314 'LDOC1'
3315 'ZNF431'
3316 'PLEKHH3'
3317 'NUBPL'
3318 'MINDY2'
3319 'GTF2H2C_2
3320 'GLRB'
3321 'APPBP2'
3322 'OR2A14'
3323 'SCN1B'
3324 'C3orf20'
3325 'MXRA7'
3326 'VSIG1'
3327 'SLC16A1'
3328 'NPDC1'
3329 'MCRIP1'
3330 'NEK9'
3331 'RALGDS'
3332 'PDZK1IP1'
3333 'PIGW'
3334 'KAT6A'
3335 'BCAR1'
3336 'PDCD5'
3337 'ABL2'
3338 'PTGER2'
3339 'UBIAD1'
3340 'PINLYP'
3341 'GFOD1'
3342 'CUL9'
3343 'KCTD17'
3344 'SIKE1'
3345 'ZNF737'
3346 'ANKRD29'
3347 'FGF7'
3348 'ANO8'
3349 'RTBDN'
3350 'HOXA9'
3351 'KMT2B'
3352 'ATG2B'
3353 'XYLT2'
3354 'SHC1'
3355 'GOPC'
3356 'SOX4'
3357 'ZNF644'
3358 'CASTOR1'

3359 'TMEM198'
3360 'KLHL4'
3361 'SH2B2'
3362 'CEP170'
3363 'RSPRY1'
3364 'SYNPO2'
3365 'SYNGAP1'
3366 'TBC1D22B'
3367 'KIF13B'
3368 'VIPAS39'
3369 'TOB2'
3370 'SIPA1L2'
3371 'KIF26B'
3372 'DAXX'
3373 'MOSPD1'
3374 'ALAD'
3375 'PDHB'
3376 'VDAC3'
3377 'INPP5B'
3378 'PPM1J'
3379 'SOCS5'
3380 'DNASE1L1'
3381 'MYO5A'
3382 'NAIP'
3383 'PEX26'
3384 'RTL8C'
3385 'LIFR'
3386 'TMEM79'
3387 'CBX2'
3388 'CCDC9B'
3389 'CCNO'
3390 'PARP15'
3391 'SDC4'
3392 'ADCK1'
3393 'IGSF8'
3394 'ZDHHC11B'
3395 'HK2'
3396 'SH3BP5'
3397 'SRP54'
3398 'GPD1L'
3399 'LACC1'
3400 'SH3RF2'
3401 'ENTPD7'
3402 'SPARC'
3403 'ADAMTS16'
3404 'MAPK1IP1L'
3405 'ACSS2'
3406 'MTX3'
3407 'ZSCAN30'
3408 'TM9SF1'
3409 'MAP3K10'
3410 'GMFB'
3411 'ADAT2'
3412 'MIER1'
3413 'GPR137C'
3414 'USP35'

3415 'PRR14L'
3416 'OAF'
3417 'ZNF420'
3418 'MAN2A2'
3419 'RHOBTB2'
3420 'ACAA2'
3421 'AHCYL2'
3422 'EXTL2'
3423 'NPHP3'
3424 'KCND1'
3425 'C1R'
3426 'GGT7'
3427 'LOC1027242
3428 'CKB'
3429 'TIMP1'
3430 'ZNF23'
3431 'PNKP'
3432 'RNFT1'
3433 'RAB4B'
3434 'PBDC1'
3435 'NMNAT1'
3436 'FSTL3'
3437 'SGK1'
3438 'MACF1'
3439 'PPP2R5C'
3440 'LMBRD2'
3441 'ZC3H10'
3442 'C10orf88'
3443 'TINF2'
3444 'DEPDC7'
3445 'RGS19'
3446 'EMC9'
3447 'WSB1'
3448 'CFAP47'
3449 'FAM222A'
3450 'TEP1'
3451 'GLIS1'
3452 'CCNL2'
3453 'SLC12A6'
3454 'MARK4'
3455 'HEXIM1'
3456 'GKAP1'
3457 'BCDIN3D'
3458 '100996414'
3459 'ZNF618'
3460 'PPP3CB'
3461 'OXCT2'
3462 'ARHGAP5'
3463 'SMAD2'
3464 'RPL36AL'
3465 'BASP1'
3466 'RAB43'
3467 'SKAP2'
3468 'CBLB'
3469 'ABHD14A'
3470 'C9orf16'

3471 'TRMT1'
3472 'CCDC50'
3473 'TBC1D3L'
3474 'DET1'
3475 'ZFP30'
3476 'OR2A1'
3477 'ZNF589'
3478 'ZNF587'
3479 'MED13L'
3480 'CHIC2'
3481 'PPP1R3E'
3482 'PLAU'
3483 'SGSM2'
3484 'SEMA3C'
3485 'PROSER3'
3486 'POLI'
3487 'FAM171A2'
3488 'LPIN3'
3489 'PTRH2'
3490 'RIPOR1'
3491 'ZNF41'
3492 'ZNF81'
3493 'AAK1'
3494 'MLH3'
3495 'HOXB2'
3496 'SLC39A9'
3497 'HOXB5'
3498 'PI4KB'
3499 'C19orf54'
3500 'MXD1'
3501 'CPE'
3502 'PHF12'
3503 'TRAPPC2'
3504 'SLC41A1'
3505 'FNTA'
3506 'SOGA3'
3507 'GTF2H2'
3508 'FAM114A1'
3509 'R3HDM2'
3510 'TMED10'

Supplementary Table 3: Common genes upregulated in A498 SUNR and AXIR

1 'MUC5B'
2 'TNS4'
3 'RPS4Y1'
4 'PCSK9'
5 'ANXA8'
6 'KRT81'
7 'SNRPN'
8 'SNURF'
9 'HID1'
10 'SUSD2'
11 'C16orf45'
12 'RIN3'
13 'TIMP4'
14 'NTRK3'
15 'QPCT'
16 'TRIM29'
17 'FAM83A'
18 'RBFOX3'
19 'DOK7'
20 'MANSC1'
21 'FCGRT'
22 'MAP1LC3A'
23 'CENPV'
24 'CHGB'
25 'PCSK1N'
26 'CRADD'
27 'IGFBP2'
28 'SORT1'
29 'CD38'
30 'CNNM1'
31 'SLC45A1'
32 'GABRA3'
33 'GREM2'
34 'AMOTL1'
35 'RAB34'
36 'EHF'
37 'P4HA3'
38 'TMC5'
39 'HR'
40 'KRT7'
41 'UNC5D'
42 'CST1'
43 'CAVIN2'
44 'SPINT2'
45 'TMEM98'
46 'MYEF2'
47 'ZNF655'
48 'PDE10A'
49 'MAPK4'
50 'LINGO1'
51 'RBM20'
52 'C9orf152'
53 'IL11'
54 'ARX'

55	'LOC1019275
56	'GALNT13'
57	'CPS1'
58	'GRAMD1B'
59	'CERS1'
60	'FOXA3'
61	'JPH3'
62	'SCEL'
63	'TOX3'
64	'UNC13A'
65	'C6orf223'
66	'ANKRD30A'
67	'ASS1'
68	'MARCH4'
69	'ADRA1D'
70	'MPV17L'
71	'TC2N'
72	'NOSTRIN'
73	'GRID1'
74	'DPYSL5'
75	'INA'
76	'RPH3A'
77	'KLRC2'
78	'ALDH2'
79	'TSPAN8'
80	'ERICH5'
81	'GDF1'
82	'SPTB'
83	'RHOV'
84	'GRIN3B'
85	'PAPPA'
86	'TMEM216'
87	'WIPF1'
88	'PDE3A'
89	'GGT5'
90	'C5AR2'
91	'LMX1B'
92	'SLC6A17'
93	'LOC1053776
94	'BVES'
95	'NPY4R'
96	'WISP2'
97	'ZNF620'
98	'C1QTNF1'
99	'LAMC2'
100	'FAM155A'
101	'CFAP300'
102	'HOXB13'
103	'EREG'
104	'PLA2G16'
105	'BMPR1B'
106	'MANEAL'
107	'TUBAL3'
108	'FES'
109	'TMEM190'
110	'NR4A3'

111 'FXYD6'
112 'TCF4'
113 'KCNH2'
114 'RLN1'
115 'FKBP1B'
116 'SPDEF'
117 'NDUFA4L2'
118 'ZIC5'
119 'GALNT5'
120 'EPHA10'
121 'SIM2'
122 'PLD5'
123 'WDR72'
124 'FST'
125 'C15orf38-AP3
126 'TMEM61'
127 'REEP2'
128 'CDH23'
129 'KCNB1'
130 'VTN'
131 'PCDHA11'
132 'SLC51B'
133 'PEX11G'
134 'PDE7B'
135 'INPP1'
136 'ZNF280A'
137 'CPNE4'
138 'LOC1053776
139 'SERPINB9'
140 'AIFM3'
141 'FGB'
142 'NPNT'
143 'PPL'
144 'TMEM59L'
145 'CFAP65'
146 'SCRN1'
147 'C4BPB'
148 'XDH'
149 'ARHGAP20'
150 'DMBT1'
151 'IL17RB'
152 'SHH'
153 'PTPRB'
154 'COL4A6'
155 'LUM'
156 'OLIG1'
157 'MUC2'
158 'JAM3'
159 'RORC'
160 'TAGLN'
161 'SMIM31'
162 'VIPR1'
163 'PDE8B'
164 'TNFAIP8L2-S
165 'SLC52A3'
166 'SALL4'

167 'PDE1C'
168 'ZFP28'
169 'HAP1'
170 'RAET1E'
171 'ENO3'
172 'ERVMER34-'
173 'TOGARAM2'
174 'LOC1053743
175 'EDN1'
176 'SOWAHB'
177 'MYO15B'
178 'ULBP1'
179 'LGSN'
180 'ZNF385C'
181 'ST8SIA4'
182 'EVC2'
183 'GABRB3'
184 'PSG4'
185 'ISPD'
186 'MMP28'
187 'FAM174B'
188 'CSF2RA'
189 'BRINP1'
190 'IGSF1'
191 'SERPINI1'
192 'MN1'
193 'MSMP'
194 'LOC1122679
195 'CD1D'
196 'DHRS2'
197 'LOC1053785
198 'ZNF285'
199 'TBX1'
200 'ZNF614'
201 'NALCN'
202 'UPK1A'
203 'KRT4'
204 'GCOM1'
205 'WNT10B'
206 'NPR3'
207 'PEX6'
208 'CIB2'
209 'PADI2'
210 'ADAP1'
211 'ABHD14A'
212 'LOC1027247
213 'NOTCH3'
214 'TM4SF4'
215 'RGS11'
216 'IL2RG'
217 'MTSS1'
218 'MSLN'
219 'RAET1G'
220 'KCNJ2'
221 'CYP2S1'
222 'OTP'

223 'FLRT3'
224 'KLHL3'
225 'ABCC8'
226 'SYN1'
227 'PHYHIPL'
228 'ST14'
229 'THSD4'
230 'NFASC'
231 'PTK7'
232 'TCF21'
233 'SLC9A3R2'
234 'SLC16A6'
235 'CSGALNACT
236 'ZNF185'
237 'SH3RF3'
238 'GNRH2'
239 'NPY4R2'
240 'WNT4'
241 'PALM3'
242 'SLX4IP'
243 'ENTPD2'
244 'TM7SF2'
245 'DIO2'
246 'SGPP2'
247 'LOC728392'
248 'ITGA10'
249 'SELENOP'
250 'FRMD3'
251 'BMPER'
252 'ZNF806'
253 'TGM2'
254 'LOC1027241
255 'TMEM156'
256 'DDIT4L'
257 'ADCY5'
258 'LSMEM2'
259 'PGM5'
260 'PAQR5'
261 'CNTNAP3B'
262 'APOD'
263 'SLC16A5'
264 'ABCA3'
265 'OSBP2'
266 'RHBDL3'
267 'HIST1H2AI'
268 'GALNT17'
269 'TJP3'
270 'IFITM2'
271 'BMP4'
272 'DLL4'
273 'SEPT4'
274 'NOXO1'
275 'AFAP1'
276 '112267922'
277 'CST2'
278 'ADRB2'

279 'ANKRD20A4'
280 'PSG5'
281 'BIN2'
282 'GPR37'
283 'ARHGEF9'
284 'LARGE2'
285 'ANKRD1'
286 'PHACTR2'
287 'FAM167B'
288 'SCD5'
289 'ALPP'
290 'PECAM1'
291 'DNAH3'
292 'GALNT16'
293 'NEIL1'
294 'OR51E2'
295 'NRXN2'
296 'CXCL1'
297 'GOLT1A'
298 'FBLIM1'
299 'GPCPD1'
300 'PLAU'
301 'HIST2H4B'
302 'HIST2H4A'
303 'KCNN1'
304 'ARHGEF38'
305 'LPAR5'
306 'BDH1'
307 'CPT1C'
308 'COL17A1'
309 'PCDHB6'
310 'HIST1H3H'
311 'TSPAN18'
312 'C1GALT1C1L
313 'AQP3'
314 'SYNPO2'
315 'CCL20'
316 'PRSS23'
317 'FAM83E'
318 'CD22'
319 'TMEM105'
320 'STEAP2'
321 'CELF2'
322 'IGSF9B'
323 'ESRP1'
324 'ADGRG6'
325 'LTB'
326 'KLK10'
327 'CFD'
328 'HLA-DMB'
329 'CCDC106'
330 'BMP8B'
331 'ACOX2'
332 'RASGRP4'
333 'TCP10L'
334 'PCDHGA2'

335 'BMP7'
336 'CEACAM1'
337 'SERPINA3'
338 'CLIP3'
339 'KHK'
340 'PPM1E'
341 'ZNF853'
342 'CCDC181'
343 'HIST1H2BO'
344 'EPPK1'
345 'ARSG'
346 'PCDHGA7'
347 'SYP'
348 'MADCAM1'
349 'PRH1-TAS2F
350 'C2orf15'
351 'INHBE'
352 'TMEM91'
353 'TMSB4X'
354 'GRIK2'
355 'JUP'
356 'HYAL1'
357 'NES'
358 'CHD5'
359 'GFI1'
360 'ZNF214'
361 'DENND1C'
362 'CADM4'
363 'STEAP1'
364 'COL16A1'
365 'FXYD3'
366 'LOC150051'
367 'RND1'
368 'MPL'
369 'KLHDC8A'
370 'ADAP2'
371 'DGKA'
372 'ARMH1'
373 'CXADR'
374 'SYPL2'
375 'ROR2'
376 'WAS'
377 'HIST1H2AK'
378 'BDKRB2'
379 'SYT12'
380 'FAM189A2'
381 'MYPN'
382 'LETM2'
383 'FSCN2'
384 'MYORG'
385 'TRIM67'
386 'EXOC3L2'
387 'CD55'
388 'MAFK'
389 'SARM1'
390 'ADARB1'

391 'NT5E'
392 'IGSF11'
393 'SIK1B'
394 'LOC1079846
395 'TM4SF1'
396 'KIAA1324L'
397 'NKPD1'
398 'SLC6A20'
399 'FOXD4L5'
400 'PSAPL1'
401 'NR4A1'
402 'PGBD1'
403 'MFAP5'
404 'SNAP25'
405 'EPB41L4A'
406 'CTIF'
407 'CPEB4'
408 'BIK'
409 'TP53I11'
410 'FCGBP'
411 'ZFHX2'
412 'HBEGF'
413 'GGT1'
414 'HIST1H2AC'
415 'CD302'
416 'DNASE1L2'
417 'SLC45A4'
418 'SLIT2'
419 'SYCP2'
420 'NAPSA'
421 'TMTC2'
422 'CELSR3'
423 'NYAP1'
424 '105373132'
425 'PRKCG'
426 'ABHD1'
427 'EDIL3'
428 'H1FX'
429 'GJD3'
430 'PCDHB11'
431 'TNNT2'
432 'ODF3B'
433 'LOC388436'
434 'LOC79999'
435 'RNFT2'
436 'BTBD3'
437 'ANGEL1'
438 'EFL1'
439 'LCP1'
440 'N4BP3'
441 'TP63'
442 'EFR3B'
443 'SLC23A2'
444 'PI3'
445 'S100A16'
446 'RINL'

447 'PLD3'
448 'LAG3'
449 'NR4A2'
450 'PARD6A'
451 'CNTNAP3C'
452 'TTC9'
453 'SLC12A2'
454 'PLEKHH1'
455 'SLC4A11'
456 'CYP1A1'
457 'MLC1'
458 'SCG2'
459 'SERPINB7'
460 'MROH8'
461 'WNT9A'
462 'PALMD'
463 'DUSP1'
464 '105379575'
465 'FAM241A'
466 'MYH14'
467 'C1orf56'
468 'ANXA13'
469 'ESAM'
470 'MYO7A'
471 'CRIP1'
472 'PIGZ'
473 'HDDC3'
474 'ERBB3'
475 'SIRPB1'
476 'ENO2'
477 'PPP1R3E'
478 'UPK3B'
479 'AMPH'
480 'SEMA4A'
481 'SPIB'
482 'FZD9'
483 'CASP9'
484 'LMTK3'
485 'AP4S1'
486 'IGFBP5'
487 'ARHGAP25'
488 'TTC6'
489 'ANTXR1'
490 'FCER1G'
491 'DDTL'
492 'BAIAP3'
493 'VWA7'
494 'PDE8A'
495 'LRP11'
496 'LIPH'
497 'SYT5'
498 'TIGD3'
499 'STRA6'
500 'RAPGEF4'
501 'RENBP'
502 'SPTSSA'

503 'ARFGEF3'
504 'SOX2'
505 'PLXNA2'
506 'GLDC'
507 'PORCN'
508 'C11orf52'
509 'CLCN5'
510 'ETHE1'
511 'MAN1C1'
512 'MST1R'
513 'IGF2'
514 'TUBB3'
515 'LY6G5C'
516 'VAV1'
517 'PCDHAC1'
518 'PTP4A1'
519 'SH3BP1'
520 'CHRNB4'
521 'MARCH9'
522 'POLR2L'
523 'RASGRP1'
524 'ZNF80'
525 'DCHS1'
526 'TPM2'
527 'ASAH1'
528 'TMC7'
529 'RAB15'
530 'RASGRP2'
531 'LOC1079865
532 'SPIN3'
533 'XK'
534 'C12orf76'
535 'INHA'
536 'AHNAK2'
537 'C11orf24'
538 'OAS1'
539 'SPOCK3'
540 'GLI1'
541 'MCOLN3'
542 'OR7E24'
543 'SBK2'
544 'ARMCX5-GP
545 'CNTD2'
546 'DHCR7'
547 'JCHAIN'
548 'ACP5'
549 'SPINT1'
550 'SH3TC2'
551 'GFOD1'
552 'IL1B'
553 'TMEM151A'
554 'TLE2'
555 'SMIM6'
556 'LIMCH1'
557 'LSR'
558 'SPATA7'

559 'HIST2H2BE'
560 'FURIN'
561 'BMI1'
562 'TLE6'
563 'FNTB'
564 'LOC1122682
565 'PTPRU'
566 'MEGF8'
567 'ITPRIP'
568 'LOC1019277
569 'SAT1'
570 'KCTD6'
571 'PODXL'
572 'PTPRA'
573 'TMEM254'
574 'PTMS'
575 'PRAF2'
576 'CD226'
577 'SUN3'
578 'KRT13'
579 'SLAMF8'
580 'PTGDS'
581 'RGS7'
582 'NKD1'
583 'ZSWIM5'
584 'PTPN21'
585 'FN3K'
586 'TMEM230'
587 'GPHN'
588 'FOXD4'
589 'PRAG1'
590 'ADSSL1'
591 'MCF2L'
592 'AK3'
593 'RNF19A'
594 'HGSNAT'
595 'LOC1053731
596 'LACC1'
597 'NECTIN3'
598 'FGFBP1'
599 'CD99L2'
600 'NBDY'
601 'MED13L'
602 'AMPD3'
603 'SPIN2B'
604 'EPAS1'
605 'TECPR2'
606 'CASKIN1'
607 'KLF2'
608 'SLC17A7'
609 'RFESD'
610 'SLC25A45'
611 'SLC22A4'
612 'ERMP1'
613 'C11orf74'
614 'PCYOX1L'

615 'POLN'
616 'MISP3'
617 'SYT1'
618 'PIK3IP1'
619 'TRAPPC2'
620 'MREG'
621 'IGFL2'
622 'ZNF362'
623 'MDP1'
624 'SMKR1'
625 'LOC1019285
626 'FAM198B'
627 'GPRC5A'
628 'NRGN'
629 'GP6'
630 'WDR97'
631 'CD46'
632 'LOC1019272
633 'SLC46A3'
634 'FLNB'
635 'CBLN3'
636 'ZNF654'
637 'SNORC'
638 'ZNF575'
639 'SLC27A5'
640 'PLP2'
641 'ANKRD9'
642 'ATP6V0E2'
643 'CNTN5'
644 'ARHGAP26'
645 'GVQW3'
646 'TP53I3'
647 'NEGR1'
648 'HIST1H2BD'
649 'OPN3'
650 'TMEM147'
651 'SYT11'
652 'FAM71F2'
653 'AUTS2'
654 'COMMD3-BM
655 'RCAN3'
656 'SMN1'
657 'GPR132'
658 'TNFSF15'
659 'TMEM132A'
660 'FAM72D'
661 'APBA1'
662 'ARIH2OS'
663 'PTPRR'
664 'LOC1027242
665 'RASD1'
666 'POLR2G'
667 'INSR'
668 'R3HDM2'
669 'FYB1'
670 'GALNT4'

671 'DAG1'
672 'GPR176'
673 'CBLB'
674 'RAB3D'
675 'P4HTM'
676 'NKD2'
677 'RALGAPA1'
678 'ALDH6A1'
679 'THTPA'
680 'SLC1A4'
681 'CSPG5'
682 'LCN12'
683 'HIST1H2AG'
684 'TMEM179B'
685 'SCN1B'
686 'GADD45B'
687 'LOC1053733
688 'GFRA2'
689 'KIAA1211L'
690 'MAGEA2'
691 'ANKRD20A3'
692 'NIPSNAP3B'
693 'PSG1'
694 'RASGRF1'
695 'RHCE'
696 'MFSD6'
697 'LOC1027247
698 'ATP6V0A4'
699 'NBPF1'
700 'ERG28'
701 'HCN2'
702 'POC1B'
703 'OLFM1'
704 'LTB4R'
705 'FOXO1'
706 'WDR54'
707 'AQP1'
708 'SCML1'
709 'TBCB'
710 'CD79A'
711 'CTSK'
712 'ST3GAL1'
713 'TSC22D1'
714 'HIST2H2AA4
715 'HIST2H2AA3
716 'KLF13'
717 'DOCK9'
718 'C6orf226'
719 'BCL11A'
720 'BTC'
721 'CRTC3'
722 'C4BPA'
723 'TPCN1'
724 'JAG2'
725 'PCED1A'
726 'HINT3'

727 'CLSTN1'
728 'ACER3'
729 'BDKRB1'
730 'NTN4'
731 'ELAC1'
732 'SORL1'
733 'CASK'
734 'ARL2'
735 'FOXA1'
736 'BSN'
737 'SNPH'
738 'CPLANE2'
739 'SGPP1'
740 'PRTG'
741 'BMP8A'
742 'ST3GAL5'
743 'CES3'
744 'EDA'
745 'NUTM2D'
746 'ATF5'
747 'HES4'
748 'LOC1079841
749 'SOBP'
750 'SENP8'
751 'KIAA0319'
752 'MAP3K8'
753 'KIF26A'
754 'MORN4'
755 'PAQR6'
756 'C15orf48'
757 'DHRSX'
758 'PDGFRL'
759 'C11orf80'
760 'LGR4'
761 'EPHA5'
762 'ZBED2'
763 'BIRC7'
764 'CARMIL3'
765 'SCAMP5'
766 'CDS2'
767 'ACADSB'
768 'RETREG1'
769 'TGFBR3'
770 'FANCB'
771 'PPP1R13B'
772 'FAM162A'
773 'DND1'
774 'HACD2'
775 'C15orf65'
776 'PRXL2A'
777 'CCDC92'
778 'MBTPS2'
779 'ZNF862'
780 'PPFIBP2'
781 'DGLUCY'
782 'C20orf96'

783 'SERINC2'
784 'RALGAPA2'
785 'ZNF772'
786 'RNF43'
787 'WSCD1'
788 'DBNDD1'
789 'ACBD7'
790 'LLGL2'
791 'C2CD2L'
792 'ZSCAN26'
793 'NR2E3'
794 'LSP1'
795 'TNFSF14'
796 'ZNF219'
797 'SLC25A29'
798 'RAB4B'
799 'MOSPD2'
800 'COX16'
801 'NUBPL'
802 'STAMBPL1'
803 'C10orf90'
804 'SGSM1'
805 'NECTIN4'
806 'SLC4A8'
807 'GALNT10'
808 'SPANXB1'
809 'LTBP2'
810 'ANG'
811 'GPRIN3'
812 'MAGEE1'
813 'ZNF720'
814 'LRFN3'
815 'BORCS5'
816 'DNMBP'
817 'TSPAN4'
818 'MED29'
819 'MIF4GD'
820 'TIMM17B'
821 'CAPNS1'
822 'DBNDD2'
823 'TENT5A'
824 'ULK3'
825 'ZNF337'
826 'HIST3H2A'
827 'CUX1'
828 'PXYLP1'
829 'TMEM17'
830 'GCNT3'
831 'GSK3A'
832 'ZNF846'
833 'RABAC1'
834 'SCN3B'
835 'GOLGA8H'
836 'COBL'
837 'PSORS1C1'
838 'USP11'

839 'KLC2'
840 'SLC17A5'
841 'ZNF473'
842 'FHIT'
843 'COL4A5'
844 'G6PD'
845 'LDOC1'
846 'ZDHHC11'
847 'MVD'
848 'SLC2A3'
849 'AMDHD1'
850 'TIGAR'
851 'INF2'
852 'TRIM52'
853 'IDI1'
854 'MAN2A2'
855 'SIAE'
856 'TMX4'
857 'C3'
858 'RNF141'
859 'ZBTB38'
860 'SAYSD1'
861 'TMEM187'
862 'C19orf33'
863 'MAST4'
864 'POLD4'
865 'SPRYD3'
866 'TSPAN3'
867 'RFK'
868 'DRP2'
869 'KCNJ15'
870 'FAM177B'
871 'DACT1'
872 'PROX1'
873 'CLSTN2'
874 'GLT8D2'
875 'SPARCL1'
876 'ZMAT1'
877 'ZNF799'
878 'CD7'
879 'SV2A'
880 'PEBP1'
881 'ADCK1'
882 'BAIAP2L2'
883 'HIST1H2BK'
884 'PRDM11'
885 'GMFB'
886 'TM9SF1'
887 'PLCXD2'
888 'GBP5'
889 'SLC9A3'
890 'ABCC10'
891 'LOC1027241
892 'MYO5A'
893 'DHFR2'
894 'BANF1'

895 'PRDM6'
896 'APMAP'
897 'PIGA'
898 'SPRY2'
899 'CAMTA2'
900 'TDRP'
901 'ZC3H12A'
902 'MRNIP'
903 'EPHX2'
904 'PROSER3'
905 'COQ5'
906 'CFAP58'
907 'ACSL6'
908 'GPR37L1'
909 'RAB30'
910 'DENND1B'
911 'MEIS3'
912 'SLIT3'
913 'TBX2'
914 'ODC1'
915 'FITM2'
916 'KIF13B'
917 'CYB5B'
918 'LOC1019280
919 'LRRC28'
920 'CGRRF1'
921 'PTN'
922 'BCAT2'
923 'FAM89B'
924 'NOMO1'
925 'GOLGA8R'
926 'FDFT1'
927 'PPP1R3C'
928 'ANK1'
929 'B4GALT1'
930 'KCNC3'
931 'ARSB'
932 'SLC9A7'
933 'CALM1'
934 'TMEM231'
935 'RRAGB'
936 'SLC16A2'
937 'DIRC2'
938 'INCA1'
939 'NAA80'
940 'SH3TC1'
941 'STARD10'
942 'ICMT'
943 'CARMIL2'
944 'GRK6'
945 'LTB4R2'
946 'ERF'
947 'LOC1053707
948 'PACS2'
949 'DPH3'
950 'IQSEC2'

951 'GOLGA8N'
952 'CAMK1D'
953 'CLTB'
954 'TMED10'
955 'LRP5'
956 'HNRNPLL'
957 'ACYP1'
958 'ZADH2'
959 'SDSL'
960 'ITGA7'
961 'NBEAL2'
962 'NECTIN1'
963 'TRPM4'
964 'RDM1'
965 'MRPS21'
966 'OLFM2'
967 'COQ6'
968 'HHLA2'
969 'OXCT2'
970 'RRNAD1'
971 'C14orf132'
972 'LMF1'
973 'APBB3'
974 'NADSYN1'
975 'TK2'
976 'HSD17B8'
977 'STYX'
978 'CMC1'
979 'TMOD2'
980 'SYVN1'
981 'WDR7'
982 'TMEM134'
983 'RRAS'
984 'IRS1'
985 'TKFC'
986 'SLC22A5'
987 'ERMARD'
988 'PIGH'
989 'CYP4F12'
990 'KSR1'
991 'ALS2CL'
992 'ACP6'
993 'B4GAT1'
994 'SMIM19'
995 'BAD'
996 'CALM3'
997 'PSEN1'
998 'FAM149B1'
999 'NEK9'
1000 'FUCA1'
1001 'RBM14-RBM[illegible]
1002 'LOC1079874[illegible]
1003 'FOXL1'
1004 'SDCBP2'
1005 'HAVCR2'
1006 'PITPNM1'

1007 'USP27X'
1008 'ATG2B'
1009 'MEGF9'
1010 'APOPT1'
1011 'TRAPPC9'
1012 'NTN5'
1013 'C6orf89'
1014 'XYLB'
1015 'NUTM2E'
1016 'DCST2'
1017 'ARAF'
1018 'ATP6AP2'
1019 'NANOS1'
1020 'CYS1'
1021 'HIST1H4H'
1022 'GCNT4'
1023 'MAMLD1'
1024 'SERGEF'
1025 'TPP1'
1026 'GPR137'
1027 'VWDE'
1028 'FLYWCH2'
1029 'SEMA3F'
1030 'RMND5B'
1031 'C18orf32'
1032 'ACAD8'
1033 'SEMA3C'
1034 'AK9'
1035 'BRF1'
1036 'ITGA2'
1037 'DMXL2'
1038 'COMMD7'
1039 'SNX24'
1040 'NCR3LG1'
1041 'IRF2BPL'
1042 'SLC22A31'
1043 'ARHGDIB'
1044 'EID3'
1045 'SPTBN4'
1046 'SFMBT2'
1047 'LOC1010601
1048 'APLP1'
1049 'ADAMTSL4'
1050 'GM2A'
1051 'SCN9A'
1052 'ARPP19'
1053 'EMC7'
1054 'PCM1'
1055 'NOP9'
1056 'ACVR2B'
1057 'CEP89'
1058 'TP53INP2'
1059 'VPS37C'
1060 'POMT2'
1061 'PCYOX1'
1062 'HDHD2'

1063 'ERLIN2'
1064 'SGSM2'
1065 'STN1'
1066 'FAM111A'
1067 'ERGIC1'
1068 'TSR2'
1069 'SMIM29'
1070 'LANCL1'
1071 'ROBO1'
1072 'NIPA1'
1073 'AMIGO3'
1074 'CCDC25'
1075 '101929747'
1076 'TMEM178A'
1077 'GOLGA8M'
1078 'TIAM1'
1079 'GPRASP1'
1080 'C19orf54'
1081 'CTNNBIP1'
1082 'BAMBI'
1083 'FAM110A'
1084 'MTMR9'
1085 'FNTA'
1086 'ALG8'
1087 'FAM102B'
1088 'RAI2'
1089 'SERTAD1'
1090 'BCL9L'
1091 'CYFIP2'
1092 'TGM1'
1093 'PLSCR4'
1094 'SEL1L'
1095 'HCCS'
1096 'LPCAT4'
1097 'GSTO2'
1098 'TBC1D25'
1099 'RNGTT'
1100 'ARHGAP5'
1101 'HDAC5'
1102 'CGGBP1'
1103 'ALOXE3'
1104 'MMP24OS'
1105 'VIPAS39'
1106 'PCBD2'
1107 'KLHL28'
1108 'TMEM205'
1109 'ST7'
1110 'COA4'
1111 'RP2'
1112 'CMC4'
1113 'ZFYVE1'
1114 'ERN1'
1115 'PACS1'
1116 'FLVCR2'
1117 'ERMAP'
1118 'GRIK5'

1119	'SELENON'
1120	'CTGF'
1121	'TMC6'
1122	'CLPTM1'
1123	'PPDPF'
1124	'ZDHHC2'
1125	'SHBG'
1126	'SOS2'
1127	'ITGB2'
1128	'ANKRD46'
1129	'CAPRIN2'
1130	'APOOL'
1131	'TCTA'
1132	'MAPK1IP1L'
1133	'PIGM'
1134	'KLC1'
1135	'KBTBD11'
1136	'SLC9A6'
1137	'PIGV'
1138	'UGGT2'
1139	'TXNDC15'
1140	'PHYH'
1141	'TRIM35'
1142	'RHEBL1'
1143	'BICRAL'
1144	'CHST7'
1145	'RHOF'
1146	'OVGP1'
1147	'VAMP2'
1148	'LYRM9'
1149	'EXT2'
1150	'TAB3'
1151	'PRKAB1'
1152	'TUBB4A'
1153	'GOLGA8O'
1154	'PARD3B'
1155	'NPAS2'
1156	'DUSP8'
1157	'TMEM251'
1158	'EXD2'
1159	'MTRNR2L8'
1160	'NFAT5'
1161	'TPRG1L'
1162	'ZNF574'
1163	'NPIPA7'
1164	'CCDC85A'
1165	'CALHM5'
1166	'PITPNC1'
1167	'C21orf91'
1168	'LGR6'
1169	'TFAP2C'
1170	'PLPBP'
1171	'SYNGR2'
1172	'SEMA4F'
1173	'AHCYL2'
1174	'C10orf88'

1175 'TIMP1'
1176 'TRIM3'
1177 'CLCN4'
1178 'C1orf115'
1179 'DHRS7'
1180 'RASA2'
1181 'DSP'
1182 'SEC22C'
1183 'PLEKHN1'
1184 'SH3BGRL'
1185 'RNF166'
1186 'FAM3A'
1187 'MMGT1'
1188 'KCNK6'
1189 'RAB27B'
1190 'NDFIP1'
1191 'SOCS5'
1192 'TIMM10B'
1193 'C12orf49'
1194 'SNX14'
1195 'ZNF343'
1196 'ANAPC11'
1197 'CYSTM1'
1198 'CMTM1'
1199 'C11orf54'
1200 'NRSN2'
1201 'PAFAH2'
1202 'OXTR'
1203 'C11orf71'
1204 'MALL'
1205 'PARVG'
1206 'ZNF517'
1207 'PAK4'
1208 'RAB6A'
1209 'SLC39A9'
1210 'TAZ'
1211 'RPS6KB2'
1212 'IGIP'
1213 'RASSF8'
1214 'LAMA5'
1215 'LEPROTL1'
1216 'CPA4'
1217 'TEAD1'
1218 'PPM1H'
1219 'PGAP3'
1220 'CST3'
1221 'EEFSEC'
1222 'LOC1079839
1223 'ATL3'
1224 'WDR11'
1225 'TTC5'
1226 'EPHB3'
1227 'AKTIP'
1228 'PKN1'
1229 'BSG'
1230 'TMED3'

1231 'EXOC5'
1232 'CHD7'
1233 'CRELD1'
1234 'SRR'
1235 'NDST2'
1236 'ZNF546'
1237 'MVK'
1238 'GPAM'
1239 'ZBTB42'
1240 'GABBR1'
1241 'TMEM191B'
1242 'COMMD10'
1243 'HSF2BP'
1244 'MEF2D'
1245 'ZNF449'
1246 'KMT2B'
1247 'KCNH3'
1248 'SLC25A41'
1249 'NHSL2'
1250 'KCNQ2'
1251 'FAM57B'
1252 'TMEM42'
1253 'ERO1A'
1254 'DPAGT1'
1255 'RPS29'
1256 'B4GALT6'
1257 'DNAJC16'
1258 'TMEM106B'
1259 'YIPF6'
1260 'MICU3'
1261 'PEX11B'
1262 'MINDY3'
1263 'TDRKH'
1264 'MAP2K5'
1265 'CH507-42P1[illegible]
1266 'ADM5'
1267 'ITGAM'
1268 'KATNAL2'
1269 'HMGCL'
1270 'MAGIX'
1271 'C1RL'
1272 'C2orf81'
1273 'SYNJ2BP'
1274 'DYNLT3'
1275 'ARV1'
1276 'GANAB'
1277 'FKBP1A'
1278 'RTTN'
1279 'TMEM109'
1280 'ETV4'
1281 'H6PD'
1282 'MICA'
1283 'ITIH4'
1284 'ITGB1'
1285 'NMNAT1'
1286 'C20orf27'

1287 'AKT1'
1288 'CLMN'
1289 'ITGA6'
1290 'CFAP53'
1291 'TMEM175'
1292 'NT5C2'
1293 'CAST'
1294 'SLC35E2B'
1295 'LIFR'
1296 'ACP2'
1297 'TIAF1'
1298 'RUNDC3B'
1299 'REEP3'
1300 'BCDIN3D'
1301 'FEM1B'
1302 'HP1BP3'
1303 'ABCA7'
1304 'EIF1AD'
1305 'UBIAD1'
1306 'LRIF1'
1307 'UBE2D4'
1308 'SUSD6'
1309 'LOC1019286
1310 'GOLGA8J'
1311 'B3GNT9'
1312 'C12orf4'
1313 'C12orf60'
1314 'UHRF1BP1'
1315 'CELF6'
1316 'ZDHHC24'
1317 'PDSS2'
1318 'MTRNR2L10'
1319 'RTL8C'
1320 'OGDH'
1321 'GDPD5'
1322 'AGTPBP1'
1323 'VWA5B2'
1324 'NPC2'
1325 'TUBB2A'
1326 'DENND6A'
1327 'PSD4'
1328 'ZFHX3'
1329 'CD81'
1330 'LPP'
1331 'KIF5C'
1332 'AJM1'
1333 'RAB1B'
1334 'ARRB1'
1335 'ZNF567'
1336 'NEDD8'
1337 'INTS5'
1338 'LGMN'
1339 'ZSCAN21'
1340 'LPGAT1'
1341 'CD151'
1342 'ATP6V1D'

1343 'SLC35A5'
1344 'ZNF526'
1345 'NPDC1'
1346 'AKT2'
1347 'VPS36'
1348 'CRIPT'
1349 'SLC9B2'
1350 'MUT'
1351 'ALDH4A1'
1352 'HTRA1'
1353 'SOCS2'
1354 'GALM'
1355 'UBE3D'
1356 'BTNL9'
1357 'MAVS'
1358 'ENPP4'
1359 'KIFC2'
1360 'PRSS27'
1361 'RUFY2'
1362 'MST1'
1363 'HIPK3'
1364 'GLRB'
1365 'NOVA1'
1366 'TRAPPC6B'
1367 'ZNF589'
1368 'IL15'
1369 'PLA2G4C'
1370 'LIMD1'
1371 'GPR157'
1372 'ZNF419'
1373 'KAT14'
1374 'ZXDB'
1375 'BATF2'
1376 'SCARB1'
1377 'ZC3H10'
1378 'TOX4'
1379 'KLHL33'
1380 'SEC31B'
1381 'RAD51B'
1382 'FAM135A'
1383 'PDE9A'
1384 'MARCH2'
1385 'TINF2'
1386 'UPRT'
1387 'ZNF81'
1388 'NBPF15'
1389 'TCP11L2'
1390 'OGA'
1391 'CAPZB'
1392 'ADAM8'
1393 'UBE3B'
1394 'TCP11L1'
1395 'ATP2A2'
1396 'PNRC2'
1397 'KLHL22'
1398 'INTS9'

1399	'LOC1009967
1400	'UNC13B'
1401	'RPS10-NUDT
1402	'OLIG2'
1403	'LOC1079870
1404	'CHI3L1'
1405	'CPXM2'
1406	'TMC8'
1407	'ELAVL3'
1408	'NACAD'
1409	'ALOX15B'
1410	'ALPI'
1411	'GPR162'
1412	'GPM6B'
1413	'INHBC'
1414	'ZNF788P'
1415	'PAIP2B'
1416	'SLC25A53'
1417	'LPL'
1418	'VPS13C'
1419	'TMEM255A'
1420	'COL20A1'
1421	'CACNG7'
1422	'TMEM267'
1423	'ZNF69'
1424	'MICALCL'
1425	'CLCA2'
1426	'ABHD2'
1427	'LTBP3'
1428	'WDR25'
1429	'ZNF48'
1430	'ACTA2'
1431	'NPIPB9'
1432	'GLTP'
1433	'KRTCAP2'
1434	'CYB561A3'
1435	'RNF214'
1436	'TAMM41'
1437	'TUBB2B'
1438	'CDK6'
1439	'MLH3'
1440	'TEP1'
1441	'AAAS'
1442	'BCLAF3'
1443	'ALDH7A1'
1444	'MAP3K10'
1445	'SRGN'
1446	'CARF'
1447	'GALNT18'
1448	'HAUS5'
1449	'ELK1'
1450	'SLC26A2'
1451	'KCNN4'
1452	'CYR61'
1453	'ZDHHC23'
1454	'TMEM253'

1455 'THAP10'
1456 'GINM1'
1457 'ACYP2'
1458 'TOGARAM1'
1459 'KIAA0391'
1460 'PTDSS2'
1461 'YPEL1'
1462 'LRRC56'
1463 'SHLD1'
1464 'TMX2'
1465 'HTD2'
1466 'TTC34'
1467 'KCND1'
1468 'SUPT20HL2'
1469 'HSPB6'
1470 'CABLES2'
1471 'IL17RE'
1472 'KANK3'
1473 'RTN3'
1474 'RHPN1'
1475 'UBD'
1476 'AAMDC'
1477 'FLNA'
1478 'TTC17'
1479 'TSPAN6'
1480 'SDHAF2'
1481 'MTRNR2L2'
1482 'KLHL30'
1483 'TSC22D3'
1484 'THBD'
1485 'MSMO1'
1486 'ZNF34'
1487 'LOC1079860
1488 'MC1R'
1489 'STK35'
1490 'HCFC2'
1491 'IKZF2'
1492 'HFE'
1493 'ZNF30'
1494 'ITGB5'
1495 'LIME1'
1496 'DOCK5'
1497 'IL6ST'
1498 'ABCA5'
1499 'SMG9'
1500 'STK26'
1501 'PANO1'
1502 'PGPEP1'
1503 'TMEM138'
1504 'TUFT1'
1505 'KAT6A'
1506 'MBLAC2'
1507 'ONECUT1'
1508 'PANK4'
1509 'ZNF224'
1510 'RPS27L'

1511 'CLCF1'
1512 'CIT'
1513 'LIN7B'
1514 'LOC1079874
1515 'PCLO'
1516 'SMIM10L2A'
1517 'SLC22A23'
1518 'PPP1R14C'
1519 'FXYD5'
1520 'CD177'
1521 'TTC13'
1522 'CNIH2'
1523 'BLCAP'
1524 'SLC29A1'
1525 'REEP6'
1526 'RTL5'
1527 'ACACB'
1528 'IL18BP'
1529 'OAS3'
1530 'CDC42EP3'
1531 'SERPINB8'
1532 'CD320'
1533 'PHF21B'
1534 'GDPGP1'
1535 'NFKBIE'
1536 'FBXL20'
1537 'SLC36A1'
1538 'SRGAP3'
1539 'ABCC3'
1540 'FOXA2'
1541 'CLIC2'
1542 'MCMDC2'
1543 'MAGI3'
1544 'SLC8A2'
1545 'SH3PXD2A'
1546 'CCPG1'
1547 'MGST3'
1548 'NAT1'
1549 'MAP2K1'
1550 'SOX4'
1551 'LOC390937'
1552 'TSGA10'
1553 'C1GALT1C1'
1554 'ANKRD37'
1555 'APH1B'
1556 'TPPP'
1557 'SIPA1L3'
1558 'RASL10A'
1559 'PLPP6'
1560 'HOXC13'
1561 'P3H4'
1562 'NPIPB13'
1563 'RAD9A'
1564 'FKBP9'
1565 'DOC2A'
1566 'KRTAP2-3'

1567 'SQLE'
1568 'MYL12A'
1569 'HYAL3'
1570 'LOC1079857
1571 'AIP'
1572 'GDI1'
1573 'PPP1R9A'
1574 'ARHGEF18'
1575 'ZFAND4'
1576 'FRMD6'
1577 'ZFYVE26'
1578 'PDZD11'
1579 'PLCL2'
1580 'DBP'
1581 'LMBRD2'
1582 'ZNF274'
1583 'LOC1009966
1584 'NAALADL2'
1585 'XKR6'
1586 'PSD'
1587 'NCF1'
1588 'SSPN'
1589 'PLEKHG3'
1590 'CEP126'
1591 'CYSRT1'
1592 'GDAP1'
1593 'CALCOCO1'
1594 'FUT4'
1595 'ELL2'
1596 'NFKBIA'
1597 'C5AR1'
1598 'EFEMP2'
1599 'ACSS2'
1600 'BTBD9'
1601 'CNNM2'
1602 'RABGGTA'
1603 'KLHL17'
1604 'SHISA9'
1605 'ZBTB3'
1606 'SH2D6'
1607 'IQCD'
1608 'ISG20'
1609 'FICD'
1610 'DNAJB2'
1611 'PHF7'
1612 'BTBD11'
1613 'DNAJC27'
1614 'SERF1B'
1615 'ACTR3B'
1616 'EMC1'
1617 'HIST1H1C'
1618 'RASSF1'
1619 'PRX'
1620 'LOC401478'
1621 'ARHGEF10'
1622 'CFAP44'

1623 'EIF4G3'
1624 'CCSER2'
1625 'TMEM143'
1626 'MOCS2'
1627 'FAM200A'
1628 'NAGK'
1629 'ITPRID2'
1630 'ARSA'
1631 'SELENOW'
1632 'POLI'
1633 'MTRNR2L9'
1634 'SLC37A2'
1635 'AMT'
1636 'MAN2B2'
1637 'C4orf46'
1638 'IER2'
1639 'ARHGAP45'
1640 'NUDT17'
1641 'SSBP2'
1642 'PCNX2'
1643 'SIDT2'
1644 'GSK3B'
1645 'ARSK'
1646 'LOC1079842
1647 'SIX4'
1648 'LY6E'
1649 'GAA'
1650 'RNF150'
1651 'NSFL1C'
1652 'PTP4A3'
1653 'GAN'
1654 'GPR89B'
1655 'NECTIN2'
1656 'BBS5'
1657 'NLRX1'
1658 'ZKSCAN4'
1659 'RRAGD'
1660 'ZNF527'
1661 'TAP1'
1662 'RAD17'
1663 'ARL6IP5'
1664 'LOC1053769
1665 'SCD'
1666 'ABHD15'
1667 'C11orf45'
1668 'EPHA2'
1669 'FAM156A'
1670 'TRAF6'
1671 'ZNF181'
1672 'C20orf194'
1673 'INPP5D'
1674 'SLC20A2'
1675 'COMMD6'
1676 'IFNGR1'
1677 'FKBP7'
1678 'GRK5'

1679 'SAA2'
1680 'PYROXD2'
1681 'OSTM1'
1682 'CELF5'
1683 'STX12'
1684 'GPD1L'
1685 'NHLRC3'
1686 'ENTPD5'
1687 'VSIR'
1688 'HSPG2'
1689 'MAFA'
1690 'SLC30A4'
1691 'C6orf203'
1692 'CDSN'
1693 'PIWIL4'
1694 'FGF1'
1695 'SULT1C4'
1696 'ABCB5'
1697 'ITGB7'
1698 'ZNF749'
1699 'CAMK2B'
1700 'FRMPD3'
1701 'KBTBD8'
1702 'GABBR2'
1703 'ANKRA2'
1704 'KCNMB4'
1705 'MANEA'
1706 'CDK5R2'
1707 'PLBD2'
1708 'FAM214A'
1709 'TLN2'
1710 'SYNGAP1'
1711 'SLC35E3'
1712 'ANKRD49'
1713 'ANO8'
1714 'LOC1005075
1715 'ZNF397'
1716 'NXPH4'
1717 'ZFP36'
1718 'CPLANE1'
1719 'NAAA'
1720 'NUDT4B'
1721 'TMEM106C'
1722 'DHX29'
1723 'NDUFA7'
1724 'CATSPERG'
1725 'GOLGA8B'
1726 'ADAM22'
1727 'MTMR11'
1728 'TRPV1'
1729 'PLEKHB1'
1730 'RDH13'
1731 'IQSEC1'
1732 'LOC1079861
1733 'IER3'
1734 'BAAT'

1735 'PYGM'
1736 'SBF2'
1737 'CUL9'
1738 'FOXC1'
1739 'RNF32'
1740 'OGT'
1741 'TMEM40'
1742 'C3orf20'
1743 'FUT10'
1744 'ZFP90'
1745 'MCOLN2'
1746 'MTRNR2L3'
1747 'STARD4'
1748 'CERS5'
1749 'FOXN2'
1750 'TBCEL'
1751 'TFPI2'
1752 'AGAP11'
1753 'GPR156'
1754 'AMN'
1755 'ARHGEF28'
1756 'TBC1D2B'
1757 'TOB1'
1758 'LDLRAP1'
1759 'TMEM50A'
1760 'HIST1H3E'
1761 'HGFAC'
1762 'TUBA4A'
1763 'VEGFB'
1764 'SUPT3H'
1765 'WBP1L'
1766 'HSD11B1L'
1767 'PRKAB2'
1768 'NEIL2'
1769 'ATP6V1FNB'
1770 'NR3C2'
1771 'MMP15'
1772 'TMEM150A'
1773 'TP53I13'
1774 'POLB'
1775 'IRGQ'
1776 'SAP25'
1777 'AVIL'
1778 'MST1L'
1779 'RASGRP3'
1780 'NCAM2'
1781 'PRKCE'
1782 'MAFB'
1783 'CNPPD1'
1784 'ILK'
1785 'OPTN'
1786 'HEXB'
1787 'TMEM136'
1788 'CPT1B'
1789 'NME4'
1790 'PWP2'

1791 'CEP164'
1792 'ZNF211'
1793 'NEMP1'
1794 'MTRNR2L1'
1795 'CLU'
1796 'DUSP6'
1797 'MMP25'
1798 'PQLC3'
1799 'NOTCH2NLE
1800 'INKA2'
1801 'CDKN1A'
1802 'CCDC17'
1803 'ACTN1'
1804 'TRAF5'
1805 'FAM241B'
1806 'ZNF596'
1807 'AMHR2'
1808 'KCNQ1'
1809 'CHRD'
1810 'TAF12'
1811 'MSANTD4'
1812 'NBPF20'
1813 'RNF26'
1814 'CKB'
1815 'RANBP6'
1816 'ZKSCAN1'
1817 'C19orf57'
1818 'PRRG1'
1819 'TNFSF12'
1820 'LETMD1'
1821 'CDCA7L'
1822 'ABHD4'
1823 'TRNP1'
1824 'DPYSL2'
1825 '107985971'
1826 'LRRC24'
1827 'AKAP6'
1828 'CDC42'
1829 'SMAD3'
1830 'FZD8'
1831 'EHD1'
1832 'ARID1A'
1833 'ULBP2'
1834 'TNFSF13B'
1835 'ADAM28'
1836 'ABCA4'
1837 'SYCE2'
1838 'LAMA4'
1839 'RAG1'
1840 'TTC14'
1841 'SLC38A9'
1842 'CLDN9'
1843 'PIDD1'
1844 'DDR1'
1845 'NCSTN'
1846 'TRIM68'

1847 'TNFRSF10A'
1848 'ZNF18'
1849 'TSPAN14'
1850 'NPIPB12'
1851 'PARP15'
1852 'CTSZ'
1853 'GOLGA8A'
1854 'KSR2'
1855 'LOC391322'
1856 'DPF1'
1857 'BORCS8'
1858 'STK19'
1859 'LBHD1'
1860 'LY6G5B'
1861 'NHS'
1862 'ENC1'
1863 'CORO2B'
1864 'HHIPL2'
1865 'FBXO36'
1866 'C3orf18'
1867 'TMEM189-UE
1868 'SULT1C2'
1869 'GPSM3'
1870 'PRELP'
1871 'KCNC4'
1872 'KLHDC7A'
1873 'NUTM2B'
1874 'NBPF10'
1875 'IQCN'
1876 'CADM1'
1877 'IDNK'
1878 'ETFRF1'
1879 'BCAS3'
1880 'DUSP5'
1881 'PERP'
1882 'NAA40'
1883 'WNK4'
1884 'STXBP6'
1885 'DDX60L'
1886 'ARL6'
1887 'SPATA12'
1888 'RIOK3'
1889 'PCNX4'
1890 'ARCN1'
1891 'BCORL1'
1892 'IFT122'
1893 'CCDC15'
1894 'KCTD11'
1895 'PC'
1896 'TMEM265'
1897 'EML2'
1898 'ZNF615'
1899 'TLL1'
1900 'SUGCT'
1901 'PRR36'
1902 'CCDC62'

1903 'EZH1'
1904 'ELOA'
1905 'PRICKLE2'
1906 'DOCK4'
1907 'TTC12'
1908 'LOC1079861
1909 'SC5D'
1910 'COL11A2'
1911 'KCNJ14'
1912 'MTRNR2L6'
1913 'FAM89A'
1914 'ZNF789'
1915 'NOXA1'
1916 'LPAR2'
1917 'TMED7-TICA
1918 'TMIE'
1919 'CD109'
1920 'EXOC6B'
1921 'B3GNT7'
1922 'MTX3'
1923 'ANKRD36B'
1924 'GGT7'
1925 'KRBA2'
1926 'TUBG2'
1927 'SAMD8'
1928 'PTPRF'
1929 'CRTC1'
1930 'LOC1079846
1931 'EXTL3'
1932 'PAFAH1B2'
1933 'UTS2B'
1934 'LAD1'
1935 'SPIRE2'
1936 'NUTM2A'
1937 'LIN7C'
1938 'APOE'
1939 'CROT'
1940 'PMEPA1'
1941 'FYCO1'
1942 'STOML1'
1943 'LYPD3'
1944 'KNDC1'
1945 'MLPH'
1946 'ATP10A'
1947 'CLSTN3'
1948 'PPP2R5B'
1949 'CCBE1'
1950 'HMGCS1'
1951 'RSRP1'
1952 'PPM1N'
1953 'SYNC'
1954 'MT2A'
1955 'GYS1'
1956 'AGO3'
1957 'CCDC69'
1958 'ODAPH'

1959 'TMEM74'
1960 'ZFYVE9'
1961 'S1PR3'
1962 'FAM160B1'
1963 'CAMKMT'
1964 'PKD1L2'
1965 'COLGALT2'
1966 'HIST1H2BC'
1967 'ZBTB49'
1968 'PALM2-AKAF
1969 'OTUD1'
1970 'SPACA9'
1971 'NBPF14'
1972 'SLPI'
1973 'INAFM2'
1974 'WDR44'
1975 'HERC3'
1976 'MAP3K12'
1977 'VKORC1'
1978 'MAPK8IP2'
1979 'ATG16L2'
1980 'BIRC2'
1981 'SLC43A2'
1982 'ARL15'
1983 'TBC1D3H'
1984 'ZNF561'
1985 'TOLLIP'
1986 'CTSB'
1987 'EIF3CL'
1988 'TSPAN1'
1989 'ZSWIM6'
1990 'TP53INP1'
1991 'IRAK2'
1992 'TMEM35B'
1993 'EML6'
1994 'LOC1079842
1995 'LRRN4CL'
1996 'GRK4'
1997 'ZGLP1'
1998 'NEU1'
1999 'SH2B2'
2000 'MAP2K3'
2001 'CMPK2'
2002 'PER2'
2003 'NKIRAS2'
2004 'SFR1'
2005 'CAP2'
2006 'TMEM182'
2007 'TMEM236'
2008 'C1orf226'
2009 'HINFP'
2010 'BNIP3L'
2011 'WNT3'
2012 'KPNA5'
2013 'FAR1'
2014 'ARID4A'

2015 'TRAM2'
2016 'ZNF792'
2017 'PLCG2'
2018 'TMEM59'
2019 'FAM131B'
2020 'MICAL2'
2021 'SMURF2'
2022 'PPOX'
2023 'TCTN1'
2024 'TRAM1'
2025 'GATA3'
2026 'QSOX1'
2027 'SNX19'
2028 'RELT'
2029 'PIGS'
2030 'PBLD'
2031 'NUDCD3'
2032 'ATM'
2033 'ARC'
2034 'MOSMO'
2035 'TSNARE1'
2036 'ITIH2'
2037 'C15orf62'
2038 'RIMBP3C'
2039 'IQCH'
2040 'NRBP2'
2041 'NPIPB11'
2042 'MCTP2'
2043 'SLC41A2'
2044 'ATP8B1'
2045 'EDA2R'
2046 'TM7SF3'
2047 'ZNF493'
2048 'CDKAL1'
2049 'TGFB1I1'
2050 'SLC39A13'
2051 'EFNA2'
2052 'TCEA2'
2053 'NPIPA8'
2054 'DOCK3'
2055 'YAP1'
2056 'PDK4'
2057 'UBAP1L'
2058 'FLAD1'
2059 'LCA5'
2060 'CITED1'
2061 'CCNO'
2062 'GOLGA6L3'
2063 'GALNT2'
2064 'PHLDB3'
2065 'DYRK1B'
2066 'SMPD1'
2067 'BTD'
2068 'AKAP13'
2069 'PAX9'
2070 'NPIPB4'

2071 'KLF7'
2072 'RUNDC1'
2073 'PPARGC1B'
2074 'RNF208'
2075 'ISYNA1'
2076 'FAM222A'
2077 'KCTD17'
2078 'ATP8B2'
2079 'PLAUR'
2080 'SLC30A1'
2081 'NUDT16'
2082 'KBTBD3'
2083 'S100A11'
2084 'CHRNA10'
2085 'RHPN2'
2086 'MARCKS'
2087 'NCALD'
2088 'MYO5B'
2089 'SLC16A4'
2090 'ARID3A'
2091 'HPS5'
2092 'CEMIP2'
2093 'LONRF2'
2094 'NPIPA2'
2095 'PRICKLE4'
2096 'SP6'
2097 'SESN1'
2098 'DNASE1L1'
2099 'LOC1079872
2100 'PRPF40B'
2101 'TTC32'
2102 'SMIM14'
2103 'MANBA'
2104 'SCX'
2105 'SLC12A7'
2106 'FAM228B'
2107 'PLOD1'
2108 'CD274'
2109 'FNDC3B'
2110 'HOXA1'
2111 'ACER2'
2112 'APLP2'
2113 'UBE4A'
2114 'TRIM21'
2115 'ZNF512B'

Supplementary Table 4: Common genes upregulated in 786-O SUNR and AXIR and A498 SUNR and AXIR

1	'LIMCH1'
2	'PRXL2A'
3	'TNFSF12'
4	'AUTS2'
5	'RRAGD'
6	'TP53I11'
7	'SUN3'
8	'IGFBP2'
9	'TMEM40'
10	'ERICH5'
11	'C4BPB'
12	'TMEM98'
13	'AQP1'
14	'PHLDB3'
15	'APBA1'
16	'TUBB4A'
17	'CELF2'
18	'TAGLN'
19	'ERVMER34-1'
20	'ADAM28'
21	'ATP8B2'
22	'LRFN3'
23	'RENBP'
24	'TSPAN18'
25	'PCSK9'
26	'MLPH'
27	'FAM89A'
28	'GATA3'
29	'FCGRT'
30	'SORL1'
31	'KCNK6'
32	'KLHL30'
33	'LPL'
34	'NPR3'
35	'C11orf52'
36	'PSG5'
37	'HIST1H3E'
38	'PIK3IP1'
39	'NOXA1'
40	'PLXNA2'
41	'C1orf115'
42	'AMDHD1'
43	'PHF21B'
44	'MYH14'
45	'MEIS3'
46	'IGFBP5'
47	'XK'
48	'PCDHB11'
49	'TFAP2C'
50	'LY6G5C'
51	'PSG1'
52	'SFMBT2'
53	'C11orf45'
54	'GRIN3B'

55	'TMEM59L'
56	'TNNT2'
57	'NPNT'
58	'FN3K'
59	'OAS1'
60	'NME4'
61	'IL2RG'
62	'ATP8B1'
63	'CPA4'
64	'PSG4'
65	'PLEKHB1'
66	'MARCH4'
67	'PDGFRL'
68	'PAX9'
69	'ZNF362'
70	'DGKA'
71	'CYP1A1'
72	'TRAF5'
73	'KRT81'
74	'PEX11G'
75	'MYO7A'
76	'MCF2L'
77	'TMEM236'
78	'ALPI'
79	'CSF2RA'
80	'CCDC85A'
81	'PTP4A3'
82	'BIRC7'
83	'SCD5'
84	'ADRB2'
85	'C5AR1'
86	'CYS1'
87	'HIST1H2AK'
88	'HID1'
89	'GALNT18'
90	'IGFL2'
91	'SLC4A8'
92	'GJD3'
93	'RASGRP3'
94	'FAM189A2'
95	'NEIL1'
96	'MN1'
97	'CXADR'
98	'ALPP'
99	'RAET1G'
100	'ASS1'
101	'LOC101928589'
102	'C15orf62'
103	'GRK5'
104	'CCDC181'
105	'SEMA4A'
106	'GSTO2'
107	'PPFIBP2'
108	'GCNT4'
109	'HIST1H2AI'
110	'BATF2'

111 'DACT1'
112 'NCALD'
113 'MCOLN3'
114 'ACP5'
115 'ZNF285'
116 'CTGF'
117 'MSLN'
118 'TNFSF13B'
119 'PADI2'
120 'C1orf226'
121 'DHRS2'
122 'SPDEF'
123 'WNT4'
124 'RNF150'
125 'NRXN2'
126 'SORT1'
127 'PLA2G16'
128 'NCAM2'
129 'LCP1'
130 'LOC107984282'
131 'SLC9A3R2'
132 'SHISA9'
133 'TMC6'
134 'LYPD3'
135 'CFAP300'
136 'DBNDD1'
137 'TMEM178A'
138 'CST2'
139 'INSR'
140 'ZSWIM5'
141 'MAFA'
142 'LOC390937'
143 'ADAP1'
144 'DPYSL2'
145 'MMP25'
146 'EPB41L4A'
147 'PTK7'
148 'DBNDD2'
149 'LIPH'
150 'CEACAM1'
151 'CD177'
152 'LOC107987464'
153 'IGF2'
154 'SEPT4'
155 'GLT8D2'
156 'VSIR'
157 'SARM1'
158 'RASGRF1'
159 'KRT7'
160 'LOC101928095'
161 'YPEL1'
162 'SLC4A11'
163 'SYCP2'
164 'FGF1'
165 'HIST1H3H'
166 'LY6E'

167 'PLA2G4C'
168 'SHBG'
169 'CAVIN2'
170 'REEP2'
171 'LRRC56'
172 'NEGR1'
173 'SLC20A2'
174 'CYSTM1'
175 'GOLGA8M'
176 'CSPG5'
177 'FES'
178 'ARC'
179 'RTL5'
180 'CSGALNACT1'
181 'ETHE1'
182 'ISG20'
183 'SERPINB9'
184 'CCDC69'
185 'PLSCR4'
186 'PXYLP1'
187 'ANTXR1'
188 'COLGALT2'
189 'TMEM255A'
190 'SPTB'
191 'TUBAL3'
192 'GDAP1'
193 'TIMP4'
194 'LSR'
195 'B3GNT7'
196 'RASL10A'
197 'CERS1'
198 'BMPER'
199 'MLC1'
200 'GDF1'
201 'PCSK1N'
202 'GNRH2'
203 'IL17RB'
204 'PRKCG'
205 'VTN'
206 'CBLN3'
207 'ANKRD1'
208 'AMOTL1'
209 'PALM2-AKAP2'
210 'NXPH4'
211 'ZNF219'
212 'CADM4'
213 'GFI1'
214 'CORO2B'
215 'SERPINI1'
216 'CDK5R2'
217 'TP53I3'
218 'KPNA5'
219 'PARD3B'
220 'PRSS27'
221 'PRDM6'
222 'LOC101060179'

223 'LOC107984648'
224 'UPK1A'
225 'DCST2'
226 'OSBP2'
227 'GRIK5'
228 'SH3RF3'
229 'C9orf152'
230 'EPPK1'
231 'PHYHIPL'
232 'CLIP3'
233 'FLVCR2'
234 'C12orf60'
235 'AMPH'
236 'SC5D'
237 'PLCG2'
238 'DOK7'
239 'ARMH1'
240 'ZFHX2'
241 'IQCN'
242 'FLRT3'
243 'KIAA0319'
244 'LOC112268238'
245 'LOC107986163'
246 'RAPGEF4'
247 'ADCY5'
248 'NTN5'
249 'RINL'
250 'RHBDL3'
251 'EDA'
252 'GRID1'
253 'MST1R'
254 'CELF6'
255 'FAM83A'
256 'RGS11'
257 'AHNAK2'
258 'SYT1'
259 'CTIF'
260 'ULBP1'
261 'TLE2'
262 'AFAP1'
263 'ZFAND4'
264 'NKPD1'
265 'HGFAC'
266 'PAIP2B'
267 'CD22'
268 'SLC46A3'
269 'RRAGB'
270 'PODXL'
271 'C20orf96'
272 'PLEKHH1'
273 'MAFK'
274 'GOLGA8R'
275 'TMEM136'
276 'PLCXD2'
277 'RBM20'
278 'GRK4'

279 'CLU'
280 'WDR97'
281 'MISP3'
282 'THBD'
283 'ABHD4'
284 'PAPPA'
285 'CPT1B'
286 'PSORS1C1'
287 'PTPRU'
288 'NPY4R2'
289 '112267922'
290 'SGSM1'
291 'NAALADL2'
292 'SH2D6'
293 'POLN'
294 'SNORC'
295 'NOTCH3'
296 'SH3TC1'
297 'FOXD4L5'
298 'ST8SIA4'
299 'MMP28'
300 'DENND1C'
301 'FAM167B'
302 'IDNK'
303 'GOLGA8J'
304 'ACAD8'
305 'SLIT2'
306 'LOC401478'
307 'TJP3'
308 'ARFGEF3'
309 'PRTG'
310 'HIST1H1C'
311 'NEU1'
312 'HIST1H2AC'
313 'ISYNA1'
314 'EHD1'
315 'TRPM4'
316 'SNPH'
317 'ZNF846'
318 'PARD6A'
319 'TTC14'
320 'FAM71F2'
321 'MYO5B'
322 'GDPD5'
323 'PHYH'
324 'MST1L'
325 'C11orf54'
326 'TTC6'
327 'MROH8'
328 'RETREG1'
329 'GOLGA6L3'
330 'OGT'
331 'PPARGC1B'
332 'NLRX1'
333 'CDS2'
334 'ADSSL1'

335 'PAQR6'
336 'MARCH2'
337 'GPR176'
338 'LTBP2'
339 'HPS5'
340 'TPP1'
341 'NANOS1'
342 'OSTM1'
343 'FKBP9'
344 'TRIM21'
345 'ACP2'
346 'TK2'
347 'SLC17A5'
348 'LAD1'
349 'FAM102B'
350 'FBXO36'
351 'NPC2'
352 'EFEMP2'
353 'RAB30'
354 'NR3C2'
355 'HINFP'
356 'ARHGEF28'
357 'PDZD11'
358 'LCN12'
359 'CCPG1'
360 'LOC100996720'
361 'MMP15'
362 'CNTNAP3B'
363 'ZDHHC11'
364 'LGR4'
365 'RHPN1'
366 'TUBB2A'
367 'ZFYVE26'
368 'FOXA1'
369 'ITGA6'
370 'LOC101927503'
371 'OLIG2'
372 'FST'
373 'CD226'
374 'PKD1L2'
375 'NOSTRIN'
376 'RASGRP4'
377 'KRBA2'
378 'ZNF385C'
379 'TIGD3'
380 'ABCA4'
381 'GGT5'
382 'PDE7B'
383 'SYPL2'
384 'IL1B'
385 'FAM177B'
386 'LSP1'
387 'SOBP'
388 'NIPSNAP3B'
389 'SUSD2'
390 'KLK10'

391 'C14orf132'
392 'PYGM'
393 'SELENOP'
394 'SBK2'
395 'NCF1'
396 'XDH'
397 'FCGBP'
398 'HNRNPLL'
399 'GABBR2'
400 'GPRASP1'
401 'C19orf33'
402 'RALGAPA2'
403 'TUBB2B'
404 'PPP1R3C'
405 'HIST1H2BK'
406 'GOLGA8N'
407 'NKIRAS2'
408 'RIN3'
409 'TAP1'
410 'INCA1'
411 'ASAH1'
412 'UPK3B'
413 'MTRNR2L3'
414 'PRICKLE4'
415 'NPIPB9'
416 'SNX19'
417 'GCNT3'
418 'HIST1H2BD'
419 'YAP1'
420 'ZNF181'
421 'FOXC1'
422 'CITED1'
423 'MYEF2'
424 'NYAP1'
425 'ZNF792'
426 'YIPF6'
427 'PIWIL4'
428 'GABRB3'
429 'ST3GAL1'
430 'C2orf15'
431 'SLC9A7'
432 'SDCBP2'
433 'ERBB3'
434 'TBCEL'
435 'ABCA5'
436 'USP11'
437 'TGFBR3'
438 'SNX24'
439 'APOOL'
440 'NUTM2B'
441 'FAM110A'
442 'IFNGR1'
443 'IFT122'
444 'FAM241B'
445 'LOC105376906'
446 'ARRB1'

447 'TRPV1'
448 'MSANTD4'
449 'CIB2'
450 'RAG1'
451 'CDCA7L'
452 'COL11A2'
453 'APOE'
454 'DCHS1'
455 'RIOK3'
456 'NR4A2'
457 'TCP11L1'
458 'PIDD1'
459 'BAMBI'
460 'ADAMTSL4'
461 'SIAE'
462 'TGFB1I1'
463 'RNF214'
464 'ST14'
465 'CAMKMT'
466 'PCED1A'
467 'PGPEP1'
468 'TPRG1L'
469 'UBE4A'
470 'CDSN'
471 'CHD5'
472 'TRAM2'
473 'CCSER2'
474 'SSPN'
475 'NECTIN2'
476 'LIN7C'
477 'BNIP3L'
478 'LOC107987269'
479 'CHRNA10'
480 'MGST3'
481 'TPPP'
482 'SULT1C4'
483 'CEP126'
484 'NPIPB13'
485 'ANKRD36B'
486 'PRKCE'
487 'OPTN'
488 'NR4A1'
489 'CEP164'
490 'NAGK'
491 'PPM1N'
492 'TUFT1'
493 'C2CD2L'
494 'FSCN2'
495 'CRTC1'
496 'ZSCAN26'
497 'RALGAPA1'
498 'GINM1'
499 'TOLLIP'
500 'ZNF18'
501 'SIRPB1'
502 'MCOLN2'

503 'CAP2'
504 'ABHD15'
505 'PGBD1'
506 'FNDC3B'
507 'STAMBPL1'
508 'IL17RE'
509 'KBTBD11'
510 'TMIE'
511 'DOCK5'
512 'LOC107984115'
513 'REEP3'
514 'BMP8B'
515 'MARCKS'
516 '101929747'
517 'NSFL1C'
518 'FRMPD3'
519 'ATM'
520 'LTB4R2'
521 'SLC16A2'
522 'ARHGDIB'
523 'UHRF1BP1'
524 'NFKBIE'
525 'ILK'
526 'IRS1'
527 'DNAJB2'
528 'WBP1L'
529 'XKR6'
530 'BMP7'
531 'RUFY2'
532 'BBS5'
533 'ZNF517'
534 'ZFYVE9'
535 'NPIPB4'
536 'MTRNR2L1'
537 'SLC36A1'
538 'SBF2'
539 'CNPPD1'
540 'ACSL6'
541 'CCDC62'
542 'TP53I13'
543 'IRGQ'
544 'LOC105373102'
545 'DYRK1B'
546 'ZBTB3'
547 'NPIPB11'
548 'IKZF2'
549 'SLC23A2'
550 'G6PD'
551 'PYROXD2'
552 'DNAJC27'
553 'MICA'
554 'ARHGEF18'
555 'PRX'
556 'WIPF1'
557 'RMND5B'
558 'INPP1'

559 'PQLC3'
560 'FHIT'
561 'HIPK3'
562 'THTPA'
563 'TEAD1'
564 'ARHGEF10'
565 'HIST1H2BO'
566 'ARSK'
567 'ABCC10'
568 'VAMP2'
569 'MTRNR2L6'
570 'HIST1H4H'
571 'BCAS3'
572 'TRIM68'
573 'DPAGT1'
574 'HYAL1'
575 'ZNF274'
576 'CD55'
577 'SUGCT'
578 'ALDH7A1'
579 'NPIPB12'
580 'TTC17'
581 'STK19'
582 'LRRC28'
583 'LY6G5B'
584 'WNT3'
585 'CTSB'
586 'ANKRD49'
587 'PCNX4'
588 'OXTR'
589 'FUT10'
590 'PACS2'
591 'TOGARAM1'
592 'CEMIP2'
593 'PHACTR2'
594 'APLP2'
595 'BTBD3'
596 'MT2A'
597 'ABCC3'
598 'PLD3'
599 'SMIM31'
600 'RASGRP2'
601 'AVIL'
602 'SERPINA3'
603 'CPNE4'
604 'IGSF11'
605 'ELAVL3'
606 'QPCT'
607 'ONECUT1'
608 'HOXC13'
609 'KCNJ2'
610 'ANKRD20A3'
611 'FXYD6'
612 'PPL'
613 'DNAH3'
614 'HR'

615 'CACNG7'
616 'SYT5'
617 'TMC5'
618 'CAMK2B'
619 'AMN'
620 'MADCAM1'
621 'CHRD'
622 'CD1D'
623 'OLFM2'
624 'EIF1AD'
625 'EXTL3'
626 'DDX60L'
627 'C20orf194'
628 'TMEM230'
629 'MTRNR2L8'
630 'TMOD2'
631 'MAVS'
632 'SCRN1'
633 'EFL1'
634 'ARCN1'
635 'CARF'
636 'ACP6'
637 'DUSP8'
638 'APMAP'
639 'C15orf65'
640 'GAN'
641 'LYRM9'
642 'ITPRID2'
643 'ADARB1'
644 'PTPRA'
645 'FUT4'
646 'EXD2'
647 'OGDH'
648 'CEP89'
649 'PCYOX1'
650 'EDIL3'
651 'RTN3'
652 'CTSK'
653 'GABBR1'
654 'MTRNR2L10'
655 'ZBTB49'
656 'SMIM29'
657 'BTBD9'
658 'TMEM179B'
659 'PSEN1'
660 'ZBTB42'
661 'LOC107984203'
662 'IL6ST'
663 'NUDT17'
664 'ZKSCAN4'
665 'HBEGF'
666 'MYL12A'
667 'BCL9L'
668 'SAMD8'
669 'TRIM3'
670 'HDAC5'

671 'ZNF337'
672 'ZNF527'
673 'AAMDC'
674 'CNTNAP3C'
675 'TRAF6'
676 'DND1'
677 'TDRKH'
678 'HIST2H4B'
679 'HIST2H4A'
680 'ZFYVE1'
681 'MFSD6'
682 'MOSPD2'
683 'RP2'
684 'PTPN21'
685 'CCDC17'
686 'KSR1'
687 'TUBG2'
688 'ZNF30'
689 'SLC25A45'
690 'POLB'
691 'VPS13C'
692 'TTC5'
693 'CDKAL1'
694 'PCLO'
695 'DNMBP'
696 'SLC30A1'
697 'MTRNR2L9'
698 'ATL3'
699 'NAA40'
700 'SNRPN'
701 'ZNF567'
702 'HCFC2'
703 'SDHAF2'
704 'BCLAF3'
705 'KCNJ14'
706 'BLCAP'
707 'ZNF749'
708 'KLHL28'
709 'RNF166'
710 'SYVN1'
711 'STK35'
712 'EXOC6B'
713 'TIAF1'
714 'FAM89B'
715 'NECTIN1'
716 'EML2'
717 'ATP6V1D'
718 'C6orf203'
719 'LOC107986113'
720 'ZGLP1'
721 'TTC34'
722 'ZDHHC23'
723 'FBLIM1'
724 'ESAM'
725 'TNFSF15'
726 'ZNF512B'

727 'OTUD1'
728 'BTD'
729 'KLF7'
730 'GPR137'
731 'CLCN4'
732 'GSK3B'
733 'TOB1'
734 'PRSS23'
735 'HCCS'
736 'AMPD3'
737 'ROBO1'
738 'HEXB'
739 'SNURF'
740 'C12orf49'
741 'MTMR9'
742 'ZNF419'
743 'HINT3'
744 'ULK3'
745 'EZH1'
746 'PPP2R5B'
747 'PDSS2'
748 'GPRC5A'
749 'OR7E24'
750 'WDR44'
751 'ZNF224'
752 'UPRT'
753 'AP4S1'
754 'VPS37C'
755 'SLC17A7'
756 'REEP6'
757 'CYSRT1'
758 'FOXN2'
759 'TIMM10B'
760 'LIME1'
761 'PTPRB'
762 'TMEM138'
763 'SEC31B'
764 'SUPT3H'
765 'KBTBD3'
766 'CLPTM1'
767 'FAM156A'
768 'AGO3'
769 'RAD17'
770 'ZXDB'
771 'NR4A3'
772 'RASA2'
773 'MTRNR2L2'
774 'BORCS8'
775 'TCP11L2'
776 'SOS2'
777 'TTC12'
778 'ACTN1'
779 'SPIN3'
780 'SPANXB1'
781 'RUNDC1'
782 'TMEM106B'

783	'FITM2'
784	'STEAP2'
785	'TRIM35'
786	'ZFP90'
787	'GADD45B'
788	'LOC391322'
789	'HIST1H2AG'
790	'EXT2'
791	'BIRC2'
792	'PRRG1'
793	'MINDY3'
794	'ALS2CL'
795	'MANBA'
796	'TSPAN14'
797	'OGA'
798	'FYCO1'
799	'ZNF397'
800	'IQSEC1'
801	'TECPR2'
802	'HSD11B1L'
803	'INTS5'
804	'SH3BGRL'
805	'SERTAD1'
806	'FAM214A'
807	'SCAMP5'
808	'LIMD1'
809	'CARMIL2'
810	'ARX'
811	'PDE3A'
812	'MREG'
813	'ERMARD'
814	'C6orf89'
815	'SLC38A9'
816	'GOLGA8A'
817	'POLR2G'
818	'GOLGA8H'
819	'CYR61'
820	'FAM162A'
821	'DHX29'
822	'TP53INP2'
823	'CGRRF1'
824	'FBXL20'
825	'RDH13'
826	'EIF3CL'
827	'RHPN2'
828	'SLC45A4'
829	'TMEM42'
830	'CALCOCO1'
831	'ZNF211'
832	'NUDT16'
833	'TOX4'
834	'BMP8A'
835	'FRMD3'
836	'AKAP6'
837	'SRR'
838	'PLBD2'

839 'RFESD'
840 'ARSB'
841 'BCAT2'
842 'EMC7'
843 'DUSP5'
844 'GPR162'
845 'LIN7B'
846 'SYNC'
847 'SMPD1'
848 'SMIM14'
849 'EEFSEC'
850 'PCM1'
851 'PACS1'
852 'TMEM182'
853 'KSR2'
854 'TMEM191B'
855 'FAM131B'
856 'PIGS'
857 'PAFAH1B2'
858 'FAM135A'
859 'TSGA10'
860 'UBE2D4'
861 'SLC9A6'
862 'RFK'
863 'SMURF2'
864 'C6orf226'
865 'FICD'
866 'C20orf27'
867 'C1orf56'
868 'ACYP2'
869 'TSPAN6'
870 'LPAR2'
871 'RNF19A'
872 'FAM155A'
873 'COQ5'
874 'ELL2'
875 'SRGAP3'
876 'LRRC24'
877 'HGSNAT'
878 'CHST7'
879 'SMN1'
880 'FKBP1A'
881 'BCORL1'
882 'H1FX'
883 'LTB4R'
884 'CYFIP2'
885 'ERMP1'
886 'ZNF473'
887 'MAP2K3'
888 'LPP'
889 'ZNF343'
890 'SERGEF'
891 'ZNF654'
892 'TMEM267'
893 'CAST'
894 'PIGH'

895 'C11orf71'
896 'DPF1'
897 'DHFR2'
898 'ERMAP'
899 'ANKRD46'
900 'TMX4'
901 'MED29'
902 'FEM1B'
903 'ITPRIP'
904 'LOC107987457'
905 'ITGA2'
906 'CST3'
907 'POLR2L'
908 'H6PD'
909 'TIMM17B'
910 'CADM1'
911 'UBE3D'
912 'EXOC5'
913 'CMTM1'
914 'HES4'
915 'GM2A'
916 'ZIC5'
917 'NUDCD3'
918 'TBCB'
919 'MAP2K5'
920 'RNF208'
921 'DMXL2'
922 'KAT14'
923 'NOP9'
924 'SNX14'
925 'MST1'
926 'LOC107984638'
927 'PBLD'
928 'ENO3'
929 'TKFC'
930 'MANEA'
931 'LAMC2'
932 'TP63'
933 'KLHL22'
934 'MAN2B2'
935 'CLSTN1'
936 'ZNF526'
937 'PANO1'
938 'TBC1D25'
939 'INKA2'
940 'WDR54'
941 'LRRN4CL'
942 'ENPP4'
943 'EPHA10'
944 'KCNN1'
945 'PRKAB2'
946 'CATSPERG'
947 'WAS'
948 'TRAPPC6B'
949 'SLC35A5'
950 'ZSWIM6'

951 'LBHD1'
952 'NDST2'
953 'TLN2'
954 'NUTM2E'
955 'KLC1'
956 'DOCK4'
957 'GOLGA8B'
958 'NUTM2A'
959 'APH1B'
960 'MIF4GD'
961 'SYCE2'
962 'C11orf74'
963 'LOC107986596'
964 'TENT5A'
965 'CPLANE1'
966 'AKTIP'
967 'MRPS21'
968 'HDHD2'
969 'AK3'
970 'LRIF1'
971 'LOC107983998'
972 'ARL6'
973 'INTS9'
974 'RNGTT'
975 'BICRAL'
976 'HDDC3'
977 'NFAT5'
978 'ITGB1'
979 'CD151'
980 'AGAP11'
981 'SLC43A2'
982 'COQ6'
983 'HIST2H2AA4'
984 'HIST2H2AA3'
985 'GPR157'
986 'ERLIN2'
987 'ARAF'
988 'EDN1'
989 '107985971'
990 'B3GNT9'
991 'NAT1'
992 'SIPA1L3'
993 'SPOCK3'
994 'CAPNS1'
995 'NPIPA8'
996 'SELENON'
997 'ZNF788P'
998 'IQSEC2'
999 'TMEM175'
1000 'ZSCAN21'
1001 'HHIPL2'
1002 'SPIN2B'
1003 'MOCS2'
1004 'BRF1'
1005 'CTNNBIP1'
1006 'TTC13'

1007 'MAP2K1'
1008 'CTSZ'
1009 'SIDT2'
1010 'DPH3'
1011 'SERF1B'
1012 'DYNLT3'
1013 'ZDHHC2'
1014 'SHH'
1015 'KCTD11'
1016 'ZNF34'
1017 'RRAS'
1018 'SEL1L'
1019 'SLX4IP'
1020 'WDR7'
1021 'TGM1'
1022 'KRTAP2-3'
1023 'FAM228B'
1024 'CPEB4'
1025 'NUTM2D'
1026 'ATP6AP2'
1027 'TMSB4X'
1028 'SAP25'
1029 'NBPF1'
1030 'UNC13B'
1031 'CHD7'
1032 'TMEM59'
1033 'SENP8'
1034 'FRMD6'
1035 'LDOC1'
1036 'NUBPL'
1037 'GLRB'
1038 'SCN1B'
1039 'C3orf20'
1040 'NPDC1'
1041 'NEK9'
1042 'KAT6A'
1043 'UBIAD1'
1044 'GFOD1'
1045 'CUL9'
1046 'KCTD17'
1047 'ANO8'
1048 'KMT2B'
1049 'ATG2B'
1050 'SOX4'
1051 'SH2B2'
1052 'SYNPO2'
1053 'SYNGAP1'
1054 'KIF13B'
1055 'VIPAS39'
1056 'SOCS5'
1057 'DNASE1L1'
1058 'MYO5A'
1059 'RTL8C'
1060 'LIFR'
1061 'CCNO'
1062 'PARP15'

1063 'ADCK1'
1064 'GPD1L'
1065 'LACC1'
1066 'MAPK1IP1L'
1067 'ACSS2'
1068 'MTX3'
1069 'TM9SF1'
1070 'MAP3K10'
1071 'GMFB'
1072 'MAN2A2'
1073 'AHCYL2'
1074 'KCND1'
1075 'GGT7'
1076 'LOC102724250'
1077 'CKB'
1078 'TIMP1'
1079 'RAB4B'
1080 'NMNAT1'
1081 'LMBRD2'
1082 'ZC3H10'
1083 'C10orf88'
1084 'TINF2'
1085 'FAM222A'
1086 'TEP1'
1087 'BCDIN3D'
1088 'OXCT2'
1089 'ARHGAP5'
1090 'CBLB'
1091 'ABHD14A'
1092 'ZNF589'
1093 'MED13L'
1094 'PPP1R3E'
1095 'PLAU'
1096 'SGSM2'
1097 'SEMA3C'
1098 'PROSER3'
1099 'POLI'
1100 'ZNF81'
1101 'MLH3'
1102 'SLC39A9'
1103 'C19orf54'
1104 'TRAPPC2'
1105 'FNTA'
1106 'R3HDM2'
1107 'TMED10'
